# Exploring Antibiotic-Potentiating Effects of Tobramycin–Deferiprone Conjugates in *Pseudomonas aeruginosa*

**DOI:** 10.3390/antibiotics12081261

**Published:** 2023-07-31

**Authors:** Karan Gandhi, Shiv Dhiman, Rajat Arora, Danzel Marie Ramirez, Danyel Ramirez, Gilbert Arthur, Frank Schweizer

**Affiliations:** 1 Department of Chemistry, Faculty of Science, University of Manitoba, Winnipeg, MB R3T 2N2, Canada; gandhikaran2@gmail.com (K.G.); shiv.dhiman@umanitoba.ca (S.D.); arorar5@myumanitoba.ca (R.A.); ramirezd@myumanitoba.ca (D.M.R.); ramiredm@myumanitoba.ca (D.R.); 2 Department of Biochemistry and Medical Genetics, University of Manitoba, Winnipeg, MB R3E 0J9, Canada; gilbert.arthur@umanitoba.ca; 3 Department of Medical Microbiology and Infectious Diseases, University of Manitoba, Winnipeg, MB R3R 0J9, Canada

**Keywords:** antibacterial activity, antibiotic potentiation, tobramycin-deferiprone conjugates, *Pseudomonas aeruginosa*

## Abstract

Metal ions, including Fe^3+^, affect the target site binding of some antibiotics and control the porin- and siderophore-mediated uptake of antibiotics. Amphiphilic tobramycins are an emerging class of antibiotic potentiators capable of synergizing with multiple classes of antibiotics against Gram-negative bacteria, including *Pseudomonas aeruginosa*. To study how the antibiotic-potentiating effect of amphiphilic tobramycins is affected by the presence of intermolecular iron chelators, we conjugated the FDA-approved iron chelator deferiprone (DEF) to tobramycin (TOB). Three TOB-DEF conjugates differing in the length of the carbon tether were prepared and tested for antibacterial activity and synergistic relationships with a panel of antibiotics against clinical isolates of *P. aeruginosa*. While all TOB-DEF conjugates were inactive against *P. aeruginosa*, the TOB-DEF conjugates strongly synergized with outer-membrane-impermeable antibiotics, such as novobiocin and rifampicin. Among the three TOB-DEF conjugates, **1c** containing a C_12_ tether showed a remarkable and selective potentiating effect to improve the susceptibility of multidrug-resistant *P. aeruginosa* isolates to tetracyclines when compared with other antibiotics. However, the antibacterial activity and antibiotic-potentiating effect of the optimized conjugate was not enhanced under iron-depleted conditions, indicating that the function of the antibiotic potentiator is not affected by the Fe^3+^ concentration.

## 1. Introduction

**Figure 1:**
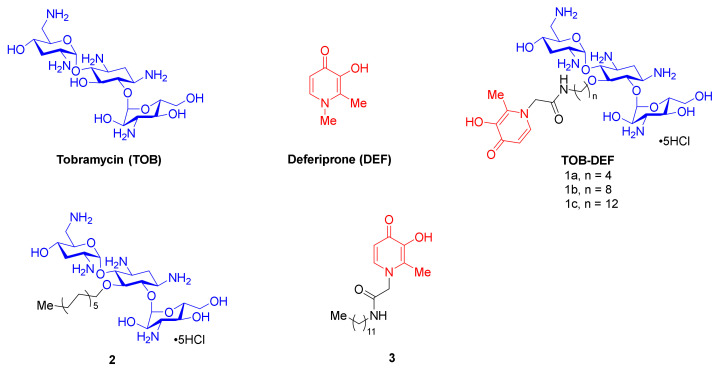
Structure of TOB-DEF conjugates **1a**–**c** and control compounds **2** and **3**.

With the rising number of multidrug-resistant (MDR) Gram-negative infections, the Centers for Disease Control and Prevention (CDC), in 2019, categorized *Pseudomonas aeruginosa (P. aeruginosa)* as a “serious threat” [1,2,3,4]. The clinical presentations caused by *P. aeruginosa* involve severe conditions, such as ventilator-associated pneumonia (VAP), urinary tract infections (UTI), and intra-abdominal infections [3,5,6]. Cystic fibrosis (CF) patients are prone to pulmonary infections by the non-mucoid strain of *P. aeruginosa*, which eventually develops into the mucoid phenotype [7,8,9]. In such cases, increased resistance is observed, which can lead to chronic infection with very few therapeutic options [10]. Current treatment options to combat MDR *P. aeruginosa* infections are limited to *β*-lactams, aminoglycosides, and polymyxins [11]. However, resistance to these antibiotics occurs frequently as a result of various resistance mechanisms, including the expression of antibiotic-inactivating enzymes, efflux pumps, modifications of the outer membrane (OM) [12], as well as the extraordinarily high impermeability of the OM. In addition, reduced expression of the OprD porin channel reduces susceptibility to *β*-lactam antibiotics, such as imipenem (IMI) [12]. To bypass the robust OM of Gram-negative bacteria (GNB), *β*-lactam-based cephalosporins conjugated to siderophores, including the recently approved cefiderocol, have been developed [13]. Siderophores are Fe^3+^-chelating molecules (e.g., pyoverdine and pyochelin) that *P. aeruginosa* releases for essential iron uptake. Siderophores are typically unaffected by the OM barrier and efflux [14,15]. Cefiderocol, used against GNB infections, such as complicated UTI and VAP, is a hybrid antibiotic of the cephalosporin ceftazidime linked to catechol 2-chloro-3,4-dihdroxybenzoic acid by a two-carbon-chain linker [13,16]. The hybrid structure of cefiderocol results in an increased intracellular concentration in the periplasm of certain GNB and superior stability against serine- and metallo-*β*-lactamases [16]. The catechol moiety in cefiderocol is responsible for extracellular Fe^3+^ complexation, mimicking the siderophore released by *P. aeruginosa* [16]. In the periplasmic space, cefiderocol inhibits penicillin-binding proteins involved in the crosslinking of the peptidoglycan chains [17]. The unique uptake mechanism of cefiderocol combined with increased stability toward *β*-lactamases enhances the activity of cefiderocol when compared with ceftazidime and ceftazidime/avibactam against certain MDR, such as *P. aeruginosa* [18]. To overcome the limited therapeutic options to treat MDR *P. aeruginosa* infections, strategies to potentiate other classes of antibiotics, devoid of potent antipseudomonal activity, are of interest [19,20,21,22]. One of these classes is the tetracyclines. Tetracyclines are a class of broad-spectrum antibiotics that interfere with protein translation and synthesis, and are used widely for respiratory infections caused by *Mycoplasma pneumoniae*, *Chlamydia pneumoniae*, and *Chlamydia psittaci* [19]. Apart from these species, tetracyclines cover several other Gram-positive and Gram-negative pathogens [23]. Tetracyclines enter the periplasm through OM porin channels, such as OmpF and OmpC [24]. However, susceptibility to tetracyclines can be decreased due to porin mutation, loss of OmpF porin channels, and reduced OM permeability [25,26,27]. In the cytoplasm, the tetracycline–Mg^2+^ complex forms a bridge to bind with the 30S bacterial ribosomal unit, thus eliciting a biological response [28]. Recent studies have highlighted that tetracycline–Mg^2+^ complexation can be hindered by the presence of iron (Fe^3+^) [29]. Several tobramycin (TOB)-based conjugates have been previously studied as tetracycline potentiators to enhance the antipseudomonal activity of tetracyclines. For instance, when the efflux pump inhibitor 1-(1-naphthylmethyl)-piperazine (NMP) was conjugated to the 5-position of TOB, sensitivity to minocycline was improved significantly [30]. Mode of action studies indicated that the TOB-NMP conjugate enhances the OM permeability of tetracyclines in *P. aeruginosa.* In contrast, structure–activity relationship studies have indicated that the amphiphilic nature of the conjugate was critical for the observed antibiotic-potentiating effect [31,32,33]. In the present work, we designed amphiphilic hybrid molecules in which the FDA-approved iron (Fe^3+^) chelator deferiprone (DEF) [34] is conjugated to TOB in the form of TOB-tether-DEF. Three TOB-DEF conjugates (**1a**–**c**, Figure 1) containing a variable hydrophobic linker were prepared to study how iron chelation and tether length affect antibiotic potentiation in amphiphilic TOB-DEF conjugates. In addition, we also prepared control compounds **2** (TOB-tether) and **3** (DEF-tether), which are partial fragments of the most effective potentiator TOB-tether-DEF conjugate **1c**, to study structure–activity relationships in more detail.

## 2. Results

### 2.1. Synthesis of TOB-DEF Conjugates ***1a**–**c*** and Control Compounds ***2*** and ***3***

**Scheme 1:**
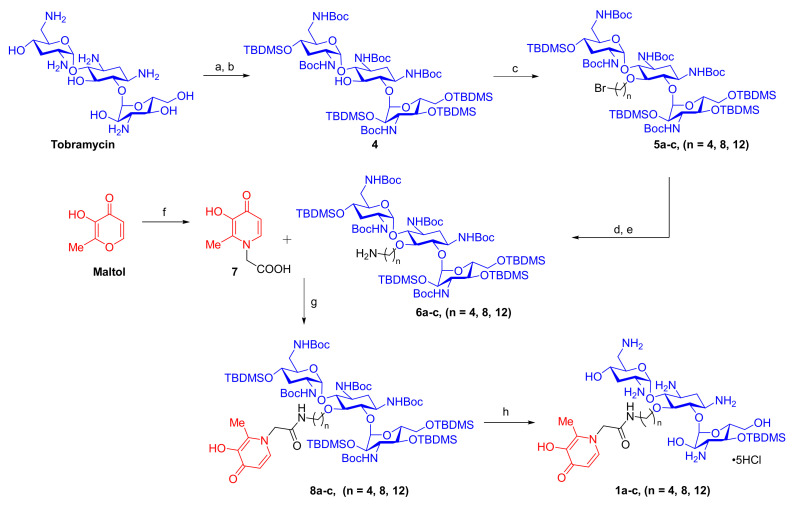
Synthesis of compounds **1a**–**c**. Reagents and conditions: (a) (Boc)_2_O, Et_3_N, and MeOH/H_2_O (2:1), RT to 55 °C, 16 h (91%); (b) TBDMS-Cl, 1-methylimidazole, and DMF, RT, 4 days (80%); (c) 1,*n*-dibromoalkane, KOH, TBAHS, and toluene, RT, 20 h (70–78%); (d) NaN_3_ and DMF, 75 °C, 6 h (>90%); (e) Pd(OH)_2_/C and H_2_, RT, 5 h (55–60%); (f) glycine, H_2_O, and NaOH (pH = 9), reflux, 20 h (62%); (g) HATU, Et_3_N, and CH_2_Cl_2_, RT, 1 h (50–60%); (h) 3M HCl in MeOH, RT, 2 h (55–68%).

**Scheme 2:**
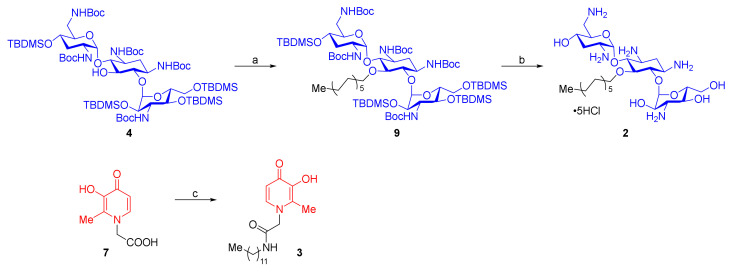
Synthesis of control compounds **2** and **3**. Reagents and conditions: (a) 1-iodododecane, KOH, TBAHS, and toluene, RT, 16 h (22%); (b) HCl/MeOH (3:2), RT, 2 h (97%); (c) HATU, Et_3_N, dodecylamine, and CH_2_Cl_2_, RT, 1 h (75%).

TOB-DEF conjugates **1a**–**c** were synthesized as outlined in Scheme 1. Initially, the amino functions of TOB were blocked with *tert*-butoxycarbonyl (Boc)-protecting groups and all hydroxyl groups, except for those at the C-5 position, were protected as silyl ethers by *tert*-butyldimethylsilyl chloride (TBDMS-Cl) to produce protected TOB analog **4**, as previously described [32]. The alkylation of alcohol **4** with various 1,*n*-dibromoalkanes produced terminal bromo-appended protected TOB **5a**–**c** that differed in the lengths of the aliphatic carbon chains with a 70–78% yield. The corresponding azides obtained in quantitative yields by the nucleophilic displacement of the bromide function using sodium azide in *N,N* dimethylformamide (DMF) at elevated temperatures were then reduced using Pd(OH)_2_ on activated carbon to produce the corresponding amines **6a**–**c** with a 55–60% yield [31]. Next, we focused on the preparation of the DEF moiety using commercially available maltol as a starting material. Maltol was subjected to an aminolysis reaction with glycine under basic conditions to produce DEF-modified analog **7** bearing carboxylic acid functionality with a 62% yield following a published procedure [35]. The coupling of TOB-tethered amines **6a**–**c** with DEF-1-acetic acid **7** was achieved using hexafluorophosphate azabenzotriazole tetramethyl uronium (HATU) as an amide coupling reagent and triethylamine (Et_3_N) as a base to produce protected TOB-DEF conjugates **8a**–**c** with a 50–60% yield. The global deprotection of Boc and TBDMS groups was achieved using HCl in methanol to produce desired TOB-DEF conjugates **1a**–**c** with a 55–68% yield (Scheme 1). We also prepared control compound **2**, a TOB-linker analog of **1c** devoid of the DEF moiety, and control compound **3**, a DEF-linker analog of **1c** devoid of the TOB moiety. For the synthesis of control compound **2**, compound **4** was alkylated with 1-iodododecane in the presence of potassium hydroxide (KOH) and tetrabutylammonium hydrogen sulfate (TBAHS) in toluene to yield **9**, and was then deprotected using methanolic HCl to obtain compound **2** with a 97% yield. Furthermore, control compound **3** was synthesized by adopting an amide coupling reaction of intermediate **7** with dodecylamine in the presence of HATU and Et_3_N (Scheme 2).

### 2.2. Antibacterial Activity of TOB-DEF Conjugates ***1a**–**c*** and Compounds ***2**–**3***

The antibacterial activity of conjugates **1a**–**c** and control compounds **2**–**3** was assessed using the broth microdilution method and tested against a panel of three GNB reference strains, including *P. aeruginosa* PAO1, *Escherichia coli* ATCC 25922, and *Acinetobacter baumannii* ATCC 17978. All compounds under study had poor antibacterial activity with minimum inhibitory concentrations (MICs) of >128 µg/mL (Table S1), characteristic of previously synthesized TOB-based antibiotic potentiators [30,31,32,33].

### 2.3. TOB-DEF Conjugates ***1a**–**c*** Potentiate Multiple Classes of Antibiotics against P. aeruginosa PAO1

**Figure 2:**
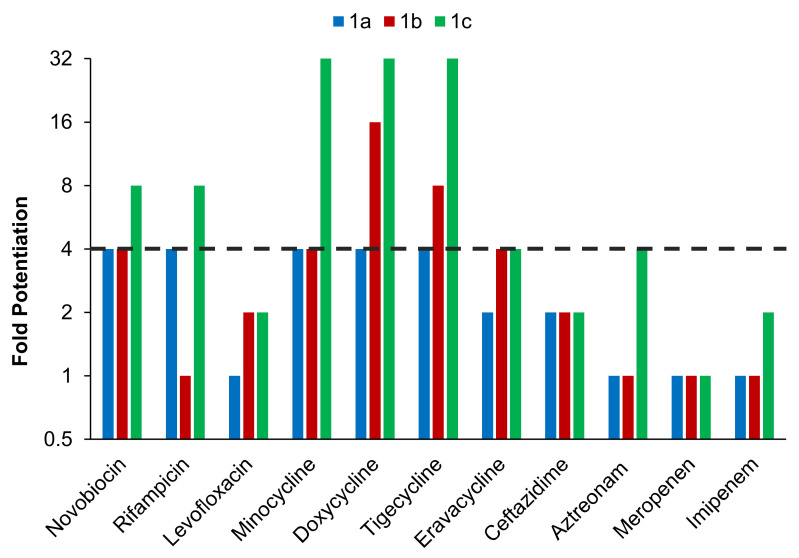
Fold potentiation of select antibiotics against wild-type *P. aeruginosa* PAO1 in the presence of 8.5 μM of TOB-DEF conjugates **1a**–**c**; a ≥4-fold potentiation indicates synergism.

Following the assessment of antibacterial activity, checkerboard assays [36] were performed to evaluate the antibiotic-potentiating effect of conjugates **1a**–**c**. The synergy of the conjugates with a diverse panel of 11 antibiotics, including OM-impermeable and OM-permeable antibiotics, was initially tested against *P. aeruginosa* PAO1 (Figure 2). *P. aeruginosa* PAO1 was selected as previous TOB-based conjugates exhibited the greatest potentiating effect against this organism [30,31,32,33]. The high molecular weight (MW >600 Da) antibiotics novobiocin (NOV) and rifampicin (RIF) were selected because of the poor OM permeability of these antibiotics against *P. aeruginosa* [30,31,32,33]. Similarly, tetracyclines were selected, as these molecules typically possess poor antibacterial activity against *P. aeruginosa* as a result of low permeability [30,31,32,33]. On the other hand, *β*-lactams like ceftazidime (CAZ) and meropenem (MER) are inactivated by *β*-lactamases and have reduced periplasmic uptake from the loss of porin channels [13,14]. Synergistic interactions corresponded to a ≥4-fold reduction in the MICs of both agents. Conjugates **1a**–**c** synergized with OM-impermeable NOV and RIF, with the exception of conjugate **1b**, which did not synergize with RIF against *P. aeruginosa* PAO1. None of the conjugates were able to potentiate levofloxacin (LEV) and the *β*-lactams MER, IMI, and CAZ, with the exception of conjugate **1c**, which reduced the MIC of aztreonam (ATM) 4-fold. Interestingly, all conjugates were able to synergize with the tetracyclines minocycline (MIN), doxycycline (DOX), tigecycline (TIG), and eravacycline (ERV), except for conjugate **1a,** which was unable to synergize with ERV. It was noted that conjugate **1c** was superior in bringing down the MICs of NOV, RIF, MIN, DOX, TIG, and ATM when compared with the shorter conjugates **1a**–**b** (Figure 2). The interaction of conjugates **1a**–**c** with the selected antibiotics was also evaluated by calculating the fractional inhibitory concentration index (FICI) (Table S2). FICI values of >0.5 but ≤4, ≤0.5, and >4 indicate no interaction, synergism, and antagonism, respectively [36]. As the combination of conjugate **1c** with various antibiotics resulted in lower FICI values, conjugate **1c** showed higher synergy with the antibiotics than conjugates **1a** and **1b**. In addition, the potentiation effect of the most potent TOB-DEF conjugate **1c** with NOV, RIF, MIN, DOX, CAZ, and ATM in PAO1 was also examined in comparison with the gold-standard OM permeabilizer polymyxin B nonapeptide (PMBN). Our studies revealed that PMBN displayed 4- to 8-fold higher potentiation of MIN, DOX, CAZ, and ATM than compound **1c**. Moreover, PMBN was more effective than compound **1c** in the potentiation of NOV (256-fold) and RIF (32-fold) (Table S2).

### 2.4. Conjugate ***1c*** Synergizes with a Panel of Antibiotics against MDR Isolates of P. aeruginosa

**Figure 3:**
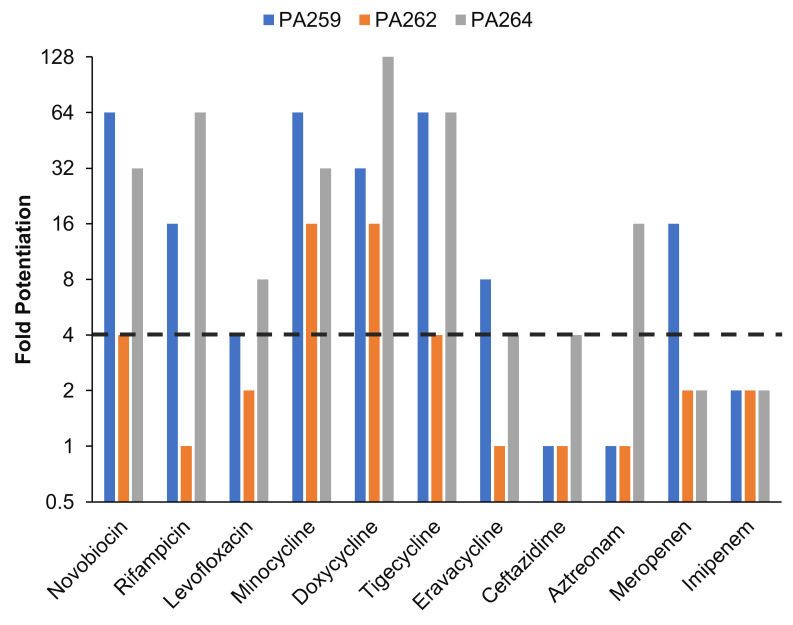
Fold potentiation of select antibiotics against MDR clinical isolates of *P. aeruginosa* in the presence of 8.5 μM of compound **1c**; a ≥4-fold potentiation indicates synergism.

Following the results against the reference strain *P. aeruginosa* PAO1, the most effective potentiator analog **1c** was selected for further study with clinical isolates of *P. aeruginosa* (Figure 3). Three *P. aeruginosa* MDR strains, PA259, PA262, and PA264, resistant to fluoroquinolones, *β*-lactams. and tetracyclines (Table S3), were used, along with the same panel of antibiotics. At first, the MIC of TOB-DEF conjugate **1c** was determined to be >128 µg/mL against all three selected isolates (Table S1). When limiting the concentration of conjugate **1c** to 8.5 μM (8 μg/mL), we observed strain-dependent antibiotic potentiation. For instance, conjugate **1c** reduced the MIC of NOV 4- to 64-fold and RIF 16- to 64-fold (except PA262), indicating that compound **1c** enhanced the permeability of RIF and NOV. Interestingly, conjugate **1c** retained excellent tetracycline potentiation. For instance, conjugate **1c** consistently reduced the MIC 16- to 64-fold for MIN, 16- to 128-fold for DOX, and 4- to 64-fold for TIG against the three MDR strains. In contrast, the MIC of ERV was only lowered 4- to 8-fold in PA259 and PA264, but not in PA262. While conjugate **1c** attained interpretative susceptibility breakpoints of MIN (≤4 µg/mL, *Acinetobacter* spp.), DOX (≤4 µg/mL, *Acinetobacter* spp.), and TIG (≤1 µg/mL, *Staphylococcus* spp.) against PA259 and PA264, the interpretative susceptibility breakpoint (≤0.5 µg/mL, *Enterobacter* spp.) of ERV was not achieved against the three MDR strains (Figure 3 and Table S4) [37]. Collectively, these results suggest that conjugate **1c** is a powerful potentiator of tetracyclines against *P. aeruginosa*.

In addition, we also studied tetracycline potentiation in a non-mucoid CF isolate PA095, which was resistant to MIN, DOX, TIG, and ERV (Table S3). While conjugate **1c** exhibited very poor antibacterial activity (>128 µg/mL) against PA095 (Table S1), the compound effectively synergized with tetracyclines. For instance, conjugate **1c** (8.5 μM) reduced the MIC of MIN, DOX, TIG, and ERV by 8–128-fold (Table S4). The observed synergistic interaction resulted in absolute MIC values of MIN (0.5 µg/mL), DOX (0.125 µg/mL), TIG (2 µg/mL), and ERV (0.5 µg/mL), indicating that the interpretative tetracycline susceptibility breakpoints can be reached in this isolate (Table S4).

### 2.5. Conjugate ***1c*** Exhibited Superior Potentiation of Tetracyclines When Compared with Control Compounds ***2*** and ***3***

**Figure 4:**
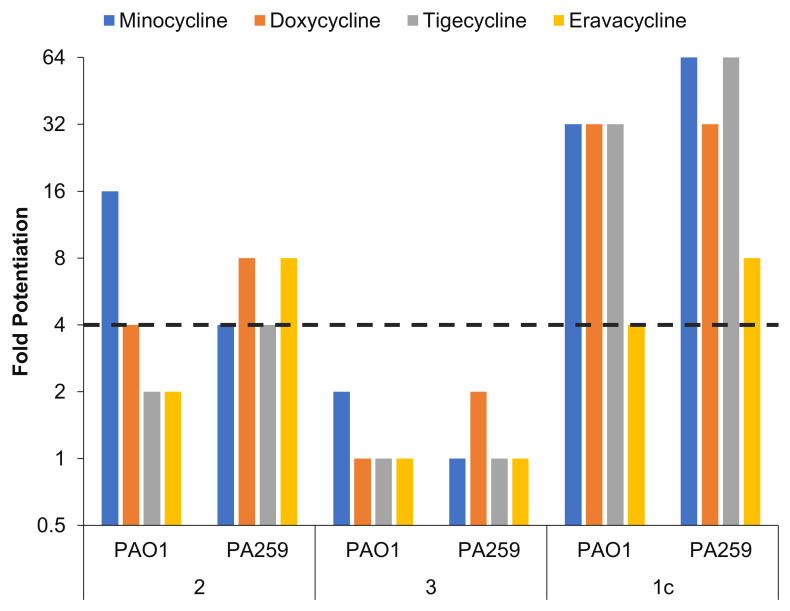
Comparison of the fold potentiation of tetracyclines against wild-type *P. aeruginosa* PAO1 and clinical isolate *P. aeruginosa* PA259 in the presence of 8.5 μM of compound **1c**, **2,** or **3**; a ≥4-fold potentiation indicates synergism.

To understand the structural implications of linking DEF to TOB, the potentiating effects of control compound **2** (conjugate **1c** without DEF) and compound **3** (conjugate **1c** without TOB) were tested in combination with tetracyclines against *P. aeruginosa* PAO1 and MDR *P. aeruginosa* PA259. In *P. aeruginosa* PAO1, compound **2** potentiated MIN and DOX 16-fold and 4-fold, respectively, while additive interactions were observed with compound **3**. Moreover, both control compounds **2** and **3** failed to synergize with TIG and ERV against PAO1. Similarly, control compound **2** potentiated all four tetracyclines in MDR PA259, but no synergy was observed with compound **3**. Overall, hybrid **1c** induced greatly enhanced tetracycline activity in comparison with control compound **2** in PAO1, indicating that both DEF and TOB were required for optimal potentiation (Figure 4 and Tables S5 and S6).

### 2.6. Tetracycline Potentiation of Conjugate ***1c*** Is Reduced under Iron-Depleted (ID) Conditions

The antibiotic-potentiating effect of conjugate **1c** was also assessed in iron-depleted cation-adjusted Mueller Hinton broth (ID-CAMHB) to probe if the molecule exhibited a mechanism similar to that of the siderophore-mediated uptake of cefiderocol [16]. Under iron-depleted (ID) conditions, the susceptibility of *P. aeruginosa* PAO1 to cefiderocol improved 8-fold, indicating that ID conditions facilitate the siderophore-dependent uptake of cefiderocol. However, no decrease in the MIC of cefiderocol was observed in ID-CAMHB with MDR *P. aeruginosa* strains PA259, PA262, and PA264 (Table S7). Interestingly, the exposure of conjugate **1c** in combination with tetracyclines (MIN, DOX, TIG, and ERV) to ID-CAMHB against the three MDR *P. aeruginosa* strains resulted in lower potentiation in the majority of cases when compared with CAMHB. This indicates that ID conditions do not enhance the tetracycline-potentiating effect of conjugate **1c** (Table 1).

### 2.7. Compound ***1c*** Disrupts the Outer Membrane of P. aeruginosa Isolates

Our preliminary checkerboard screening with TOB-DEF (**1c**) and hydrophobic antibiotics, such as NOV and RIF, against wild-type and MDR-*P. aeruginosa* isolates revealed a significant synergistic effect with NOV and RIF (Figure 2 and Figure 3). These findings suggested that compound **1c** functioned as an OM permeabilizer. To further ascertain the effect of compound **1c** on the disruption of OM integrity, we performed the checkerboard assay with compound **1c** and tetracyclines in the presence of Mg^2+^-enriched CAMHB media against wild-type PAO1 and MDR PA259. It is believed that bivalent cations reduce the negative charges of adjacent LPS molecules in the OM, thereby stabilizing the crosslinking of LPS [36,37,38]. These studies revealed that high Mg^2+^ (20 mM) concentrations abolished the MIN- and DOX-potentiating effects of **1c** in comparison with standard CAMHB media (Table 2). It is also worth noting that, in Mg^2+^-supplemented CAMHB, the MICs of MIN and DOX were also elevated 8- to 16-fold against PAO1 and PA259. Collectively, the data suggest that conjugate **1c** potentiated tetracyclines in *P. aeruginosa* by enhancing their OM uptake. These results are consistent with the tetracycline-potentiating effect of related TOB-based OM permeabilizers [30,31,32,33,34,39].

### 2.8. Cytotoxicity Study of Conjugates ***1a**–**c***

One of the concerns of membrane-active agents is the risk of inducing nonselective cytotoxicity [30,31,32]. Therefore, conjugates **1a**–**c**, positive control doxorubicin, and negative control polymyxin B were tested for cytotoxicity against the HEK293 and HepG2 cell lines. Our results indicate that longer tethers enhanced the cytotoxicity of the conjugates. The best potentiator **1c**, at its active concentration of 8.5 μM, reduced the cell viability of HEK293 and HepG2 cells to 75% and 70%, respectively, relative to those of the controls with vehicles. Moreover, increased concentrations of **1c** resulted in further reduced cell viabilities in both cell lines, which were absent in polymyxin B (Figure S1).

## 3. Discussion

Bifunctional amphiphilic TOB conjugates in which the TOB moiety is linked to antibiotics (ciprofloxacin, moxifloxacin, and RIF), efflux pump inhibitors (NMP), and metal chelators (cyclam) via a hydrophobic spacer are effective antibiotic potentiators that enhance the OM permeability of multiple classes of antibiotics [30,31,32,33]. TOB-based bifunctional conjugates are believed to destabilize the OM by displacing bivalent cations (Mg^2+^ or Ca^2+^), which are stabilizing counterions for the phosphate groups of lipid A and the phosphorylated core sugars that prevent repulsion between and among individual LPS molecules. This leads to a localized disruption of LPS in the OM, allowing non-porin-mediated passage of antibiotics into the periplasm [40,41,42,43]. The structure–activity relationships determined for bifunctional amphiphilic TOB conjugates have revealed that the nature of the spacer and its amphiphilicity are critical for optimal antibiotic potentiation [30,31,32,33]. In this study, we extended the design of bifunctional amphiphilic TOB conjugates to TOB-DEF conjugates. DEF is an FDA-approved Fe^3+^-chelator that could serve as a siderophore mimic to shuttle Fe^3+^ ions through the OM of GNB, including *P. aeruginosa*. We were interested in studying how the Fe^3+^ chelating properties affect the antibiotic-potentiating effect of amphiphilic TOB. Three TOB-DEF conjugates **1a**–**c** differing in the length of the hydrophobic spacer were prepared and lacked standalone antibacterial activity (MIC >128 μg/mL) against standard GNB reference strains, which was consistent with previous findings [30,31,32,33]. A lack of standalone antibacterial activity is desired for an antibiotic potentiator to reduce the risk of rapid resistance development [30,31,32,33]. Subsequently, conjugates **1a**–**c** were screened in combination with a panel of 11 antibiotics against the reference *P. aeruginosa* PAO1 strain. The screening indicated that TOB-DEF conjugate **1c** with the most extended spacer (C_12_) displayed the highest antibiotic-potentiating effect at a fixed concentration of 8.5 μM. Conjugate **1c** was able to synergize with OM-impermeable RIF and NOV (8-fold), consistent with a destabilizing effect of **1c** on the OM. In addition, conjugate **1c** showed high selectivity for potentiating tetracyclines against *P. aeruginosa* PAO1. For instance, we observed reductions in the MICs of DOX (64-fold) and TIG (32-fold). In contrast, conjugate **1c** did not potentiate (≤2-fold) *β*-lactams, such as CAZ, MER, and IMI, while a 4-fold potentiation was observed with ATM. The encouraging potentiating effects of **1c** in *P. aeruginosa* PAO1 prompted us to extend the study to MDR *P. aeruginosa* isolates. When compared with *P. aeruginosa* PAO1, equal, but mostly greater, antibiotic potentiation was observed in the MDR PA259 and PA264 strains, indicating that MDR *P. aeruginosa* strains were more susceptible to the antibiotic potentiation of **1c**. However, a reduced antibiotic-potentiating effect was noted in MDR PA262. When compared with all three MDR *P. aeruginosa* strains, conjugate **1c** consistently showed the greatest synergy with tetracyclines MIN, DOX, and TIG. To further understand the structural requirements of conjugate **1c** to induce selective tetracycline potentiation in *P. aeruginosa*, we explored the tetracycline-potentiating effect of control compounds **2** and **3**. These studies confirm that optimal tetracycline potentiation requires the presence of TOB, DEF, and a C_12_ spacer. We also explored the synergistic relationship of conjugate **1c** with tetracyclines in *P. aeruginosa* strains using CAMHB and ID-CAMHB to study how low Fe^3+^ concentrations affect antibiotic potentiation. These studies show that, under ID conditions, slightly reduced tetracycline potentiation and slightly reduced antibacterial activity of **1c** were observed in most MDR *P. aeruginosa* strains. Therefore, ID conditions did not improve the tetracycline potentiation of conjugate **1c**, which was unexpected and remains poorly understood. Furthermore, in order to understand the mode of action of why conjugate **1c** potentiates tetracycline antibiotics, we studied the tetracycline-potentiating effects in the presence of elevated Mg^2+^ concentrations. Traditional OM permeabilizers like PMBN compete with bivalent metal ions for the LPS binding site in the OM which typically results in greatly reduced antibacterial activity or antibiotic potentiation [40,41,42,43]. Increasing the concentration of [Mg^2+^] to 20 mM resulted in a complete loss of tetracycline potentiation, which confirms that conjugate **1c** targeted the OM of *P. aeruginosa*. Unfortunately, optimized conjugate **1c** with an amphiphilic C_12_ tether displayed greatly increased cytotoxicity against two cell lines at relevant concentrations (8.5 μM) when compared with the less amphiphilic conjugates **1a** and **1b**. Therefore, further structural optimization is required to reduce the cytotoxicity of hit compound **1c**.

## 4. Materials and Methods

### 4.1. Materials

All the reagents and solvents were purchased from Sigma Aldrich, AK Scientific Inc., and Tokyo Chemical Industry (TCI). All the intermediates and final molecules were characterized using Bruker 300, 400, and 500 MHz nuclear magnetic resonance (NMR) spectroscopy with chemical shifts (*δ*) being reported in parts per million (ppm). The different types of NMR experiments used in this study include: ^1^H, ^13^C, COSY, HSQC, HMBC, and NOESY, which were recorded in CDCl_3_ (^1^H, *δ* = 7.26; ^13^C, *δ* = 77.16), MeOD (^1^H, *δ* = 3.31; ^13^C, *δ* = 49.0), and D_2_O (^1^H, *δ* = 4.79) as internal standards. Electrospray ionization mass spectrometer (ESI-MS) and matrix-assisted laser desorption ionization mass spectrometer (MALDI-MS) spectra were obtained using a Bruker Daltonics Ultraflex MALDI–time-of-flight (TOF)/TOF mass spectrometer. All the intermediates and final compounds were purified using flash chromatography with silica gel P60 (40–63 μm) 60 Å silica gel and SiliaBond C18 (40–63 μm) 60 Å silica gel purchased from Silicycle, respectively. The purification of certain intermediates was carried out using Biotage Selekt, an automated-flash chromatography system. For purity analyses of final compounds **1a**–**c**, high-performance liquid chromatography (HPLC) was performed using a Thermo Scientific Vanquish Ultra-HPLC (Waltham, MA, USA) system connected to a Synergi 2.5 μM Polar-RP 100 Å LC column (50 mm × 2 mm, Phenomenex, Torrance, CA, USA). For inert-condition reactions, a nitrogen atmosphere was used. Compounds **4**, **5a**–**c**, **6a**–**c**, and **7** were synthesized following previously reported procedures [26].

### 4.2. Preparation of Compounds ***1a**–**c***, and ***2**–**9***

#### 4.2.1. Synthesis of 1,3,2′,6′,3″-Penta-*N*-(Tert-Butoxycarbonyl)-4′,2″,4″,6″-Tetra-O-TBDMS-tobramycin (**4**)

To a solution of tobramycin (5.0 g, 10.691 mmol) in a mixture of water (70 mL) and MeOH (140 mL), Boc-anhydride (23.34 g, 107 mmol) was added. The mixture was then charged with Et_3_N (32.821 g, 235 mmol) and refluxed at 55 °C for 16 h. The reaction mixture was concentrated under reduced pressure to obtain the crude Boc-protected tobramycin as a white solid (9.42 g, 91%). This intermediate was used without purification for the next step. TBDMS-Cl (14.01 g, 92.97 mmol) was added to a solution of Boc-protected tobramycin (9.0 g, 9.29 mmol) in dry DMF. This mixture was then charged with 1-methylimidazole (11.43 mL, 139.461 mmol) and stirred for 4 days at RT. Afterward, the DMF was removed under reduced pressure. The resulting residue was dissolved with ethyl acetate (200 mL) and washed with ice-cold water (100 mL × 3). The combined organic layers were then washed with saturated brine, dried over anhydrous Na_2_SO_4,_ filtered, and concentrated under reduced pressure. The crude product was purified using flash chromatography (15% *v/v* ethyl acetate in hexanes) to obtain compound **4** as a white solid (10.64 g, 80.36%). ^1^H NMR (500 MHz, CDCl_3_) *δ*: 5.40 (s, 1H), 5.26 (s, 1H), 4.99–4.90 (m, 2H), 4.51 (s, 1H), 4.32 (s, 1H), 3.88–3.83 (m, 2H), 3.70–3.14 (m, 13H), 2.74–2. 72 (m, 1H), 2.04–2.02 (m, 1H), 1.45–1.42 (m, 45H), 0.92–0.87 (m, 36H), 0.13–0.05 (m, 24H). MALDI TOF-MS *m/e* calcd for C_67_H_133_N_5_O_19_Si_4_Na^+^, 1446.86; measured *m/e*, 1446.80.

#### 4.2.2. General Procedure A: Preparation of 5-O-(*N*-Bromoalkane)-1,3,2′,6′,3″-Penta-*N*-(Tert-Butoxycarbonyl)-4′,2″,4″,6″-Tetra-O-TBDMS-Tobramycin (**5a**–**c**)

To a solution of compound **4** (1 equiv.) in toluene (3 mL), 1,*n*-dibromoalkane (3.0 equiv.), KOH (3.0 equiv.), and TBAHS (0.1 equiv.) were added. The reaction mixture was stirred at RT for 20 h. After completion, the toluene was evaporated under reduced pressure. Water (50 mL) was added to the crude residue and extracted with ethyl acetate (50 mL x3). The combined organic extracts were then washed with saturated brine, dried over anhydrous Na_2_SO_4_, and concentrated under reduced pressure to obtain the crude compound. Compounds **5a, 5b,** and **5c** (70–78%) were obtained as white solids after purification using flash chromatography (12% *v/v* ethyl acetate in hexanes).

#### 4.2.3. 5-O-(4-Bromobutyl)-1,3,2′,6′,3″-Penta-*N*-(Tert-Butoxycarbonyl)-4′,2″,4″,6″-Tetra-O-TBDMS-Tobramycin (**5a**)

The crude product synthesized according to general procedure A using compound **4** (1.00 g, 0.701 mmol), 1,4-dibromobutane (0.25 mL, 2.105 mmol), TBAHS (0.023 g, 0.0701 mmol), KOH (0.117 g, 2.105 mmol), and toluene (7 mL) was purified by flash chromatography (10% ethyl acetate/hexanes) to yield **5a** (0.821 g, 75%) as a white solid. ^1^H NMR (500 MHz, CDCl_3_) *δ*: 5.19 (s, 1H, H-1′), 5.13 (s, 1H, H-1″), 5.04–4.97 (m, 1H), 4.76 (s, 1H), 4.51 (s, 1H), 4.14–4.08 (m, 2H), 3.84–3.76 (m, 2H), 3.71–3.69 (m, 1H), 3.62–3.53 (m, 5H), 3.45–3.36 (m, 5H), 3.25–3.19 (m, 3H), 2.49–2.46 (m, 1H), 2.06–1.99 (m, 2H), 1.93–1.87 (m, 2H), 1.66–1.61 (m, 2H), 1.53–1.42 (m, 45H, Boc-*t*Bu), 0.95–0.86 (m, 36H, TBDMS-*t*Bu), 0.16–0.01 (m, 24H, TBDMS-SiMe_2_). MALDI TOF-MS *m/e* calcd for C_71_H_140_BrN_5_O_19_Si_4_Na^+^, 1583.2; measured *m/e*, 1582.90 [M + Na]^+^.

#### 4.2.4. 5-O-(4-Bromooctane)-1,3,2′,6′,3″-Penta-*N*-(Tert-Butoxycarbonyl)-4′,2″,4″,6″-Tetra-O-TBDMS-Tobramycin (**5b**)

The crude product synthesized according to general procedure A using compound **4** (1.5 g, 1.052 mmol), 1,8-dibromooctane (0.6 mL, 3.157 mmol), TBAHS (0.035 g, 0.105 mmol), KOH (0.176 g, 3.157 mmol), and toluene (7 mL) was purified by flash chromatography (10% ethyl acetate/hexanes) to yield **5b** (1.32 g, 78%) as a white solid. ^1^H NMR (500 MHz, CDCl_3_) *δ*: 5.22 (s, 1H, H-1′), 5.14 (s, 1H, H-1″), 5.03–5.01 (m, 1H), 4.76 (s, 1H,), 4.50 (s, 1H), 4.24 (s, 1H), 4.17–4.15 (m, 1H), 4.08–4.07 (m, 1H), 3.82–3.68 (m, 4H), 3.62–3.53 (m, 4H), 3.43–3.34 (m, 4H), 3.27–3.20 (m, 3H), 2.48–2.45 (m, 1H), 2.04–1.98 (m, 1H), 1.87–1.81 (m, 2H), 1.47–1.41 (m, 49H, Boc-*t*Bu), 1.31–1.27 (m, 6H, CH_2_ linker), 0.94–0.86 (m, 36H, TBDMS-*t*Bu), 0.15–0.02 (m, 24H, TBDMS-SiMe_2_). MALDI TOF-MS *m/e* calcd for C_75_H_148_BrN_5_O_19_Si_4_Na^+^, 1639.26; measured *m/e*, 1639.05 [M + Na]^+^.

#### 4.2.5. 5-O-(4-Bromododecane)-1,3,2′,6′,3″-Penta-*N*-(Tert-Butoxycarbonyl)-4′,2″,4″,6″-Tetra-O-TBDMS-Tobramycin (**5c**)

The crude product synthesized according to general procedure A using compound **4** (1.00 g, 0.701 mmol), 1,12-dibromododecane (0.690 g, 2.103 mmol), TBAHS (0.023 g, 0.070 mmol), KOH (0.118 g, 2.103 mmol), and toluene (7 mL) was purified by flash chromatography (10% ethyl acetate/hexanes) to yield **5c** (0.820 g, 70%) as a white solid. ^1^H NMR (500 MHz, CDCl_3_) *δ*: 5.21 (s, 1H, H-1′), 5.15 (s, 1H, H-1″), 5.06–5.04 (m, 1H), 5.04 (s, 1H), 4.77 (s, 1H), 4.50 (s, 1H), 4.26 (s, 1H), 4.17–4.16 (m, 1H), 4.08–4.06 (m, 1H), 3.78–3.64 (m, 4H), 3.65–3.61 (m, 1H), 3.55–3.51 (m, 2H), 3.41–3.33 (m, 4H), 3.27–3.17 (m, 3H), 2.48–2.45 (m, 1H), 2.02–1.98 (m, 1H), 1.88–1.82 (m, 2H), 1.59–1.53 (m, 1H), 1.45–1.41 (m, 47H, Boc-*t*Bu), 1.31–1.23 (m, 18H, CH_2_ linker), 0.94–0.86 (m, 36H, TBDMS-*t*Bu), 0.15–0.02 (m, 24H, TBDMS-SiMe_2_). MALDI TOF-MS *m/e* calcd for C_79_H_156_BrN_5_O_19_Si_4_Na^+^, 1692.95; measured *m/e*, 1692.98 [M + Na]^+^.

#### 4.2.6. Procedure B: Preparation of 5-O-(*N*-Amino-Alkylated)-1,3,2′,6′,3″-Penta-*N*-(Tert-Butoxycarbonyl)-4′,2″,4″,6″-Tetra-O-TBDMS-Tobramycin (**6a**–**c**)

To a solution of compound **5a**–**c** in dry DMF (10 mL), sodium azide (NaN_3_, 20 equiv.) was added at RT under an inert atmosphere. The reaction mixture was then heated up to 75 °C for 6 h. Afterward, the reaction mixture was concentrated under reduced pressure. The resulting residue was diluted with ethyl acetate (100 mL), washed with ice-cold water (50 mL × 3) followed by saturated brine, and dried over anhydrous Na_2_SO_4_. The resulting solution was concentrated under reduced pressure to yield the corresponding azides in a quantitative yield, which were carried forward to the next step without further purification. The crude azido compound (1.0 equiv.) was dissolved in MeOH (10 mL) and reacted with a catalytic amount of Pd(OH)_2_/C (0.1 equiv.) under H_2_ gas at RT. The reaction mixture was stirred for 5 h, followed by filtration using a celite bed, and the filtrate was then concentrated under reduced pressure. The resulting crude compound was purified by flash chromatography (10% *v/v* MeOH in dichloromethane (DCM)), which yielded **6a**–**c** (55–60%) as white solids.

#### 4.2.7. 5-O-(4-Aminobutyl)-1,3,2′,6′,3″-Penta-*N*-(Tert-Butoxycarbonyl)-4′,2″,4″,6″-Tetra-O-TBDMS-Tobramycin (**6a**)

The crude product synthesized according to general procedure B using compound **5a** (0.820 g, 0.525 mmol), NaN_3_ (0.683 g, 10.51 mmol), and Pd(OH)_2_/C (0.007 g, 0.053 mmol) was purified using flash chromatography (10% MeOH/DCM) to obtain **6a** (0.483 g, 60%). ^1^H NMR (500 MHz, CDCl_3_) *δ*: 5.24 (s, 1H, H-1′), 5.16 (s, 1H, H-1″), 5.05 (s, 1H), 4.83 (s, 1H), 4.52 (s, 1H), 4.12 (bs, 3H), 3.81–3.70 (m, 4H), 3.62–3.30 (m, 10H), 3.24–3.18 (m, 2H,), 2.66 (s, 2H), 2.52–2.46 (m, 1H), 2.01–1.99 (m, 1H), 1.57–1.52 (m, 4H), 1.45–1.42 (m, 45H), 0.95–0.87 (m, 36H, TBDMS-*t*Bu), 0.15–0.04 (m, 24H, TBDMS-SiMe_2_). MALDI TOF-MS *m/e* calcd for C_79_H_142_N_6_O_19_Si_4_Na^+^, 1517.93; measured *m/e*, 1518.87 [M + Na]^+^.

#### 4.2.8. 5-O-(8-Aminooctyl)-1,3,2′,6′,3″-Penta-*N*-(Tert-Butoxycarbonyl)-4′,2″,4″,6″-Tetra-O-TBDMS-Tobramycin (**6b**)

The crude product synthesized according to general procedure B using compound **5b** (0.500 g, 0.309 mmol), NaN_3_ (0.402 g, 6.1 mmol), and Pd(OH)_2_/C (0.005 g, 0.031 mmol) was purified using flash chromatography (10% MeOH/DCM) to obtain **6b** (0.285 g, 58%). ^1^H NMR (500 MHz, CDCl_3_) *δ*: 5.17 (s, 1H, H-1′), 5.09 (s, 1H, H-1″), 5.04–4.99 (m, 1H), 4.73 (s, 1H), 4.51 (s, 1H), 4.20–4.02 (m, 5H), 3.78–3.64 (m, 4H), 3.59–3.36 (m, 6H), 3.23–3.11 (m, 4H), 2.72 (s, 2H), 2.51–2.40 (m, 1H), 1.97–1.88 (m, 1H), 1.51–1.36 (m, 49H), 1.26–1.12 (m, 8H), 0.90–0.81 (m, 36H), 0.10–−0.03 (m, 24H).MALDI TOF-MS *m/e* calcd for C_75_H_150_N_6_O_19_Si_4_Na^+^, 1573.99; measured *m/e*, 1574.94 [M + Na]^+^.

#### 4.2.9. 5-O-(12-Aminododecyl)-1,3,2′,6′,3″-Penta-*N*-(Tert-Butoxycarbonyl)-4′,2″,4″,6″-Tetra-O-TBDMS-Tobramycin (**6c**)

The crude product synthesized according to general procedure B using compound **5c** (0.780 g, 0.466 mmol), NaN_3_ (0.606 g, 9.33), and Pd(OH)_2_/C (0.007 g, 0.047 mmol) was purified using flash chromatography (10% MeOH/DCM) to obtain **6c** (0.418 g, 55%). ^1^H NMR (400 MHz, CDCl_3_) *δ*: 5.22 (s, 1H, H-1′), 5.16 (s, 1H, H-1″), 5.08–5.03 (m, 1H), 4.80 (s, 1H), 4.55 (s, 1H), 4.28–3.95 (m, 5H), 3.82–3.69 (m, 4H), 3.63–3.35 (m, 8H), 3.28–3.21 (m, 3H), 2.79 (t, *J* = 7.5 Hz, 2H), 2.49–2.46 (m, 1H), 2.03–2.00 (m, 1H), 1.59–1.53 (m, 3H), 1.46–1.42 (m, 45H), 1.31–1.24 (m, 16H), 0.96–0.87 (m, 36H), 0.16–-0.03 (m, 24H). MALDI TOF-MS *m/e* calcd for C_79_H_158_N_6_O_19_Si_4_Na^+^, 1630.06; measured *m/e*, 1630.10 [M + Na]^+^.

#### 4.2.10. Synthesis of 2-(3-Hydroxy-4-Oxo-1,4-Dihydropyridin-1-Yl) Acetic Acid (**7**)

An oven-dried, clean, round-bottomed flask (RBF) was charged with commercially available maltol (1.20 g, 9.523 mmol) and hot water (80 mL), followed by glycine (0.357 g, 4.761 mmol). The mixture was stirred at 85 °C and the pH was adjusted to 9 using 6 M NaOH. The reaction mixture was refluxed for 20 h. Afterward, the mixture was cooled and approximately 30 mL of water was removed under reduced pressure. The pH of the remaining mixture was adjusted to 3 using 6 M HCl to afford a light-brown precipitate, which was obtained by filtration. The precipitate was then recrystallized using water and dried overnight to afford light-brown crystals of **7** (1.18 g, 62%). ^1^H NMR (500 MHz, D_2_O) *δ*: 7.95 (d, *J* = 7.0 Hz, 1H), 7.06 (d, *J* = 7.0 Hz, 1H), 4.92 (s, 2H), 2.48 (s, 3H). ^13^C NMR (125 MHz, D_2_O) *δ* 172.27, 161.07, 142.89, 141.60, 139.51, 111.23, 59.04, 12.20. ESI-MS *m/e* calcd for C_8_H_9_NO_4_Na^+^, 206.04; measured *m/e*, 206.04 [M + Na]^+^.

#### 4.2.11. General Procedure C: Amide Coupling Reaction for the Preparation of Compounds (**8a**–**c**)

To a solution of compound **7** in dry DCM (5 mL), coupling reagent HATU (1.5 equiv.) followed by Et_3_N (1.5 equiv.) were added at RT under an inert atmosphere. The reaction mixture was allowed to stir for 5–10 min; then, compound **6a, 6b**, or **6c** was added portionwise. The reaction was then stirred at RT for 1 h. Afterward, the reaction mixture was diluted with DCM (50 mL) and washed with water (30 mL), a saturated aqueous solution of sodium bicarbonate (20 mL), and then with brine solution (30 mL). The DCM layer was collected, dried over Na_2_SO_4_, and concentrated under reduced pressure. The crude compound was purified by flash chromatography using 5% *v/v* MeOH in DCM to afford desired products **8a**–**c** (50–60%).

#### 4.2.12. 5-O-(Butyl-2-(3-Hydroxy-2-Methyl-4-Oxopyridin-1(4*H*)-Yl)Acetamide)-1,3,2′,6′,3″-Penta-*N*-(Tert-Butoxycarbonyl)-4′,2″,4″,6″-Tetra-O-TBDMS-Tobramycin (**8a**)

Synthesis was conducted by following general procedure C using compounds **6a** (0.070 g, 0.046 mmol) and **7** (0.020 g, 0.093 mmol), Et_3_N (0.013 mL, 0.0935 mmol), HATU (0.035 g, 0.093 mmol), and DCM (5 mL). The crude product was purified by flash chromatography (6% *v/v* MeOH/DCM) to produce pure **8a** (0.042 g, 60%) as a light-brown solid. ^1^H NMR (500 MHz, CDCl_3_) *δ*: 7.28 (d, *J* = 7.2 Hz, 1H, H-5*_def_*), 6.40 (d, *J* = 7.2 Hz, 1H, H-6*_def_*), 5.25 (s, 1H, H-1′), 5.15 (s, 1H, H-1″), 5.05–5.04 (m, 1H), 4.73–4.63 (m, 1H), 4.51 (s, 2H, H-2′*_def_*), 4.15–4.09 (m, 1H), 3.80–3.20 (m, 17H), 3.12–3.04 (m, 1H), 2.41 (s, 1H), 2.33 (s, 3H, CH_3*def*_), 1.95–1.92 (m, 1H), 1.57–1.52 (m, 5H), 1.45–1.39 (m, 45H, Boc-*t*Bu), 0.93–0.86 (m, 36H, TBDMS-*t*Bu), 0.13–0.04 (m, 24H, TBDMS-SiMe_2_). ^13^C NMR (125 MHz, CDCl_3_) *δ*: 170.02, 165.65, 155.42, 154.85, 138.18, 128.09, 111.35, 96.06, 79.92, 79.49, 79.34, 72.48, 68.00, 63.15, 57.08, 56.15, 50.68, 48.40, 41.67, 39.85, 35.99, 28.62, 28.49, 28.48, 28.44, 27.43, 26.09, 26.03, 25.98, 25.92, 25.78, 18.47, 18.21, 18.04, 17.93, 12.09, 0.99, −3.79, −4.20, −4.84, −4.99, −5.15. MALDI TOF-MS *m/e* calcd for C_79_H_149_N_7_O_22_Si_4_Na^+^, 1682.97; measured *m/e*, 1682.94 [M + Na]^+^.

#### 4.2.13. 5-O-(Octyl-2-(3-Hydroxy-2-Methyl-4-Oxopyridin-1(4*H*)-Yl)Acetamide)-1,3,2′,6′,3″-Penta-*N*-(Tert-Butoxycarbonyl)-4′,2″,4″,6″-Tetra-O-TBDMS-Tobramycin (**8b**)

Synthesis was conducted by following general procedure C using compounds **6b** (0.380 g, 0.244 mmol) and **7** (0.107 g, 0.489 mmol), Et_3_N (0.07 mL, 0.489 mmol), HATU (0.186 g, 0.489 mmol), and DCM (5 mL). The crude product was purified by flash chromatography (6% *v/v* MeOH/DCM) to afford **8b** (0.209 g, 55%) as a light-brown solid. ^1^H NMR (500 MHz, MeOD) *δ*: 7.53 (d, *J* = 7.2 Hz, 1H, H-5*_def_*), 6.37 (d, *J* = 7.1 Hz, 1H, H-6*_def_*), 5.44–5.40 (m, 2H, H-1′, H-1″), 4.73 (s, 2H, H-2′*_def_*), 4.20–4.17 (m, 1H), 3.97–3.95 (m, 1H), 3.89 (s, 1H), 3.75–3.51 (m, 10H), 3.46–3.42 (m, 1H), 3.37–3.32 (m, 3H), 3.23–3.19 (m, 2H,), 2.31 (s, 3H, CH_3*def*_), 2.04–2.02 (m, 1H), 1.90–1.88 (m, 1H), 1.62–1.58 (m, 2H), 1.55–1.50 (m, 3H), 1.45–1.42 (m, 45H, Boc-*t*Bu), 1.33–1.23 (m, 10H,), 0.95–0.89 (m, 36H, TBDMS-*t*Bu), 0.15–0.07 (m, 24H, TBDMS-SiMe_2_). ^13^C NMR (125 MHz, MeOD) *δ*: 169.86, 166.85, 156.86, 156.67, 156.10, 155.61, 145.52, 139.01, 131.72, 111.03, 95.37, 85.21, 79.29, 79.19, 78.98, 78.78, 78.13, 76.76, 73.39, 71.16, 67.39, 63.74, 56.19, 55.50, 51.57, 48.49, 40.71, 39.42, 35.30, 30.48, 29.84, 29.21, 29.04, 27.85, 27.79, 27.69, 27.52, 27.46, 26.78, 26.31, 25.52, 25.45, 25.27, 25.19, 25.09, 18.14, 18.10, 17.66, 17.53, 17.50, 10.71, −4.72, −5.28, −5.40, −5.53, −5.61, −5.66, −5.86, −6.09, −6.35. MALDI TOF-MS *m/e* calcd for C_83_H_157_N_7_O_22_Si_4_Na^+^, 1740.53; measured *m/e*, 1740.00 [M + Na]^+^.

#### 4.2.14. 5-O-(Dodecyl-2-(3-Hydroxy-2-Methyl-4-Oxopyridin-1(4*H*)-Yl)Acetamide)-1,3,2′,6′,3″-Penta-*N*-(Tert-Butoxycarbonyl)-4′,2″,4″,6″-Tetra-O-TBDMS-Tobramycin (**8c**)

Synthesis was conducted by following general procedure D using compounds **6c** (0.450 g, 0.279 mmol) and **7** (0.092 g, 0.419 mmol), Et_3_N (0.06 mL, 0.419 mmol), HATU (0.159 g, 0.419 mmol), and DCM (5 mL). The crude product was purified by flash chromatography (6% *v/v* MeOH/DCM) to produce **8c** (0.225 g, 50%) as a light-brown solid. ^1^H NMR (500 MHz, MeOD) *δ*: 7.53 (d, *J* = 7.1 Hz, 1H, H-5*_def_*), 6.38 (d, *J* = 7.1 Hz, 1H, H-6*_def_*), 5.44 (s, 1H, H-1′), 5.40 (s, 1H, H-1″), 4.73 (s, 2H, H-2′*_def_*), 4.18–4.15 (m, 1H), 4.02–3.89 (m, 2H), 3.75–3.41 (m, 12H), 3.36–3.33 (m, 2H), 3.21 (t, *J* = 7.1 Hz, 2H), 2.31 (s, 3H, CH_3*def*_), 2.05–2.00 (m, 1H), 1.92–1.87 (m, 1H), 1.62–1.51 (m, 5H), 1.45–1.42 (m, 45H, Boc-*t*Bu), 1.33–1.28 (m, 18H, CH_2_ linker), 0.95–0.89 (m, 36H, TBDMS-*t*Bu), 0.15–0.09 (m, 24H, TBDMS-SiMe_2_). ^13^C NMR (125 MHz, MeOD) *δ*: 169.87, 166.95, 156.84, 156.65, 156.09, 155.62, 145.55, 138.99, 131.71, 111.03, 95.38, 85.18, 79.27, 79.18, 78.98, 78.78, 78.14, 76.83, 73.85, 73.40, 72.49, 71.20, 67.39, 63.73, 60.10, 56.21, 55.49, 51.56, 40.72, 39.31, 35.28, 30.84, 30.50, 29.92, 29.50, 29.43, 29.37, 29.33, 29.06, 28.97, 27.86, 27.81, 27.70, 27.53, 27.48, 26.64, 26.32, 25.57, 25.49, 25.29, 25.26, 25.22, 25.11, 19.46, 18.15, 18.10, 17.69, 17.67, 17.55, 17.51, 13.06, 10.65, −4.60, −4.69, −5.26, −5.37, −5.50, −5.60, −5.64, −5.76, −5.84, −6.07, −6.32. MALDI TOF-MS *m/e* calcd for C_87_H_165_N_7_O_22_Si_4_Na^+^, 1796.63; measured *m/e*, 1796.05 [M + Na]^+^

#### 4.2.15. 5-O-(Dodecyl)-1,3,2′,6′,3″-Penta-*N*-Boc-4′,2″,4′′,6′′-Tetra-O-TBDMS-Tobramycin (**9**)

Compound **9** was prepared using a slightly revised procedure, as previously disclosed [21]. To a solution of compound **4** (1 g, 0.701 mmol) in toluene (8 mL), iodododecane (0.52 mL, 2.103 mmol), KOH (0.12 g, 2.103 mmol), and TBAHS (0.024 g, 0.0701 mmol) were added. The reaction mixture was stirred at RT for 16 h. After completion, the toluene was evaporated under reduced pressure. The crude residue was diluted with water (10 mL), extracted with ethyl acetate (50 mL × 3), washed with saturated brine, and dried over anhydrous Na_2_SO_4_. The organic extract was then concentrated under reduced pressure. Compound **9** (0.23 g, 22%) was obtained as a white solid after purification using flash chromatography (10% *v/v* ethyl acetate in hexanes). The NMR was consistent with previously published data [21]. ^1^H NMR (500 MHz, CDCl_3_) *δ*: 5.22 (s, 1H), 5.15 (s, 1H), 5.06 (s, 1H), 4.78 (s, 1H), 4.51 (s, 1H), 4.27–4.08 (m, 3H), 3.82–3.19 (m, 16H), 2.47 (s, 1H), 2.03–2.00 (m, 1H), 1.49–1.42 (m, 45H), 1.28–1.24 (m, 18H), 0.95–0.83 (m, 39H), 0.16–0.03 (m, 24H).

#### 4.2.16. General Procedure D: Removal of All the Protecting Groups for the Preparation of Compounds (**1a**–**c**)

A clean RBF was charged with compound **8a**, **8b**, **8c**, or **9** and 3M HCl solution in MeOH (5 mL), and stirred at RT for 2 h under an inert atmosphere. After completion, the reaction mixture was concentrated under reduced pressure at 23 °C. The residue was washed with diethyl ether (5 mL × 2) and decanted to remove non-polar impurities. Compounds **1a**, **1b**, or **1c** as HCl salts were obtained after purification using reverse-phase flash chromatography (100% deionized water).

#### 4.2.17. 5-O-(Butyl-2-(3-Hydroxy-2-Methyl-4-Oxopyridin-1(4*H*)-Yl)Acetamide)-Tobramycin∙5HCl (**1a**)

The residue synthesized according to general procedure D using compound **8a** (0.042 g, 0.0253 mmol) and 3M HCl in MeOH (5 mL) was purified by C-18 reverse-phase flash chromatography to yield **1a** (0.012 g, 68%) as a dark-orange HCl salt. ^1^H NMR (500 MHz, D_2_O) *δ*: 8.06 (d, *J* = 6.5 Hz, 1H, H-5*_def_*), 7.21 (d, *J* = 6.6 Hz, 1H, H-6*_def_*), 5.40 (s, 1H, H-1′), 5.22 (s, 3H, H-1″, H-2′*_def_*), 4.28–4.21 (m, 2H, H-5′, H-5″), 3.99–3.91 (m, 4H, H-6, H-5, H-4, H-4′), 3.89–3.73 (m, 7H, H-1, H-3, H-4″, H-3″, *O*-CH_2_ linker), 3.67–3.59 (m, 3H, H-2′, H-6″), 3.43 (m, 1H, H-6′), 3.33–3.31 (m, 3H, H-6′, *N*-CH_2_ linker), 2.57–2.47 (m, 4H, H-2, CH_3_*_def_*), 2.30–2.20 (m, 2H, H-3′), 2.07–1.99 (m, 1H, H-2), 1.70–1.56 (m, 4H, CH_2_ linker). ^13^C NMR (125 MHz, D_2_O) *δ*: 166.89 (CO *amidic*), 159.74, 142.97, 142.73, 140.19 (C5*_def_*), 111.10 (C6*_def_*), 101.15 (C1″), 92.67 (C1′), 92.05, 82.12, 81.67, 76.54 (C5″), 75.56 (C5′), 73.18, 72.90, 68.61, 64.87, 63.30, 59.35, 58.06 (C2′*_def_*), 54.82, 49.66, 48.48, 47.36, 39.84 (C6′), 38.61 (*N*-CH_2_ linker), 28.11 (C3′), 27.73 (C2), 26.91 (CH_2_ linker), 25.00 (CH_2_ linker), 12.50 (CH_3_*_def_*). MALDI TOF-MS *m/e* calcd for C_31_H_54_N_6_O_12_Na^+^, 725.79; measured *m/e*, 726.25 [M + Na]^+^.

#### 4.2.18. 5-O-(Octyl-2-(3-Hydroxy-2-Methyl-4-Oxopyridin-1(4*H*)-Yl)Acetamide)-Tobramycin∙5HCl (**1b**)

The residue synthesized according to general procedure D using compound **8b** (0.209 g, 0.122 mmol) and 3 M HCl in MeOH (5 mL) was purified by C-18 reverse-phase flash chromatography to yield **1b** (0.058 g, 62%) as a dark-orange HCl salt. ^1^H NMR (500 MHz, D_2_O) *δ*: 7.67 (s, 1H, H-5*_def_*), 6.61 (s, 1H, H-6*_def_*), 5.39 (d, *J* = 5.0 Hz, 1H, H-1′), 5.18 (d, *J* = 5.2 Hz, 1H, H-1″), 4.91 (s, 2H, H-2′*_def_*), 4.30–4.27 (m, 1H, H-5′), 4.19 (t, *J* = 9.8 Hz, 1H, H-5″), 3.97–3.77 (m, 9H, H-4, H-5, H-6, H-4′, H-2″, H-3″, H-4″, *O*-CH_2_ linker), 3.75–3.70 (m, 2H, H-2′, H-1), 3.67–3.55 (m, 3H, H-2, H-6″), 3.44–3.40 (m, 1H, H-6′), 3.34–3.31 (m, 1H, H-6′), 3.28 (t, *J* = 6.8 Hz, 2H, *N*-CH_2_ linker), 2.58–2.53 (m, 1H, H-2), 2.36 (s, 3H, CH_3*def*_), 2.31–2.21 (m, 2H, H-3′), 2.03–1.95 (m, 1H, H-2), 1.66–1.65 (m, 2H, CH_2_ linker), 1.58–1.54 (m, 2H, CH_2_ linker), 1.33–1.21 (m, 8H, CH_2_ linker). ^13^C NMR (125 MHz, D_2_O) *δ*: 169.46, 168.31(CO *amidic*), 144.64, 140.06 (C5*_def_*), 135.23, 112.53 (C6*_def_*), 101.38 (C1″), 92.77 (C1′), 81.87, 76.78 (C5″), 75.76 (C5′), 73.77, 73.19, 68.55, 64.77, 63.19, 59.24, 56.53 (C2′*_def_*), 54.79, 49.78, 48.45, 47.33, 39.47 (C6′), 38.52 (*N*-CH_2_ linker), 29.43, 28.83, 28.20, 28.10 (C3′), 27.81 (C2), 25.84 (CH_2_ linker), 25.17 (CH_2_ linker), 11.73 (CH_3*def*_). MALDI TOF-MS *m/e* calcd for C_35_H_62_N_6_O_12_Na^+^, 781.90; measured *m/e*, 782.42 [M + Na]^+^.

#### 4.2.19. 5-O-(Dodecyl-2-(3-Hydroxy-2-Methyl-4-Oxopyridin-1(4*H*)-Yl)Acetamide)-Tobramycin∙5HCl (**1c**)

The residue synthesized according to general procedure D using compound **8c** (0.450 g, 0.251 mmol) and 3M HCl in MeOH (5 mL) was purified by C-18 reverse-phase flash chromatography to yield **1c** (0.112 g, 55%) as a dark-orange HCl salt. ^1^H NMR (500 MHz, D_2_O) *δ*: 7.65 (d, *J* = 7.2 Hz, 1H, H-5*_def_*), 6.58 (d, *J* = 7.1 Hz, 1H, H-6*_def_*), 5.40 (d, *J* = 2.6 Hz, 1H, H-1′), 5.18 (d, *J* = 3.5 Hz, 1H, H-1″), 4.89 (s, 2H, H-2′*_def_*), 4.29–4.26 (m, 1H, H-5′), 4.16 (t, *J* = 9.8 Hz, 1H, H-5″), 3.96–3.89 (m, 5H, H-5, H-6, H-4′, H-2″), 3.85–3.78 (m, 3H, H-4″ *O*-CH_2_ linker), 3.74–3.72 (m, 2H, H-2′, H-3″), 3.64–3.52 (m, 4H, H-1, H-2, H-6″), 3.42–3.38 (m, 1H, H-6′), 3.34–3.31 (m, 1H, H-6′), 3.28 (t, *J* = 6.8 Hz, 2H, *N*-CH_2_ linker), 2.56–2.52 (m, 1H, H-2), 2.36 (s, 3H, CH_3*def*_), 2.27–2.22 (m, 2H, H-3′), 1.99–1.91 (m, 1H, H-2), 1.65–1.64 (m, 2H, CH_2_ linker), 1.55–1.53 (m, 2H, CH_2_ linker), 1.31–1.29 (m, 16H, CH_2_ linker). ^13^C NMR (125 MHz, D_2_O) *δ*: 169.73, 169.65, 168.31 (CO *amidic*), 144.70, 140.05 (C5*_def_*), 134.75, 112.54 (C6*_def_*), 101.37 (C1″), 92.75 (C1′), 81.87, 76.84 (C5″), 75.49 (C5′), 73.20, 68.54, 64.78, 63.23, 59.25, 56.45, 54.79, 49.83, 48.45, 47.33, 39.53 (C6′), 38.56 (*N*-CH_2_ linker), 29.46 (CH_2_ linker), 28.96, 28.82, 28.70, 28.61, 28.58, 28.17, 28.12, 25.84 (CH_2_ linker), 25.31 (CH_2_ linker), 11.71 (CH_3*def*_). MALDI TOF-MS *m/e* calcd for C_39_H_70_N_6_O_12_Na^+^, 838.01; measured *m/e*, 838.48 [M + Na]^+^.

#### 4.2.20. 5-O-(Dodecyl)-Tobramycin (**2**)

Synthesis was conducted according to general procedure D using compound **9** (0.23 g, 0.144 mmol) and 3M HCl in MeOH (5 mL). The reaction mixture was concentrated under reduced pressure and the residue was washed with 2% MeOH in ether (5 mL) and decanted. The crude product was purified by C-18 reverse-phase flash chromatography to obtain **2** (0.089 g, 97%) as a white solid HCl salt. ^1^H NMR (500 MHz, D_2_O) *δ*: 5.18 (d, *J* = 2.6 Hz, 1H, H-1′), 4.97 (d, *J* = 3.5 Hz, 1H, H-1″), 4.10–4.06 (m, 1H, H-5′), 3.99 (t, *J* = 9.8 Hz, 1H, H-5″), 3.75–3.66 (m, 5H, H-4′, H-2″), 3.64–3.56 (m, 4H, H-4″), 3.51 (m, 2H, H-1, H-2′), 3.45–3.41 (m, 1H, H-3), 3.40–3.35 (m, 2H, H-6″), 3.22–3.18 (m, 1H, H-6′), 3.12–3.09 (m, 1H, H-6′), 2.35–2.31 (m, 1H, H-2), 2.09–2.00 (m, 2H, H-3′), 1.83–1.75 (m, 1H, H-2), 1.43 (m, 2H, CH_2_ linker), 1.08 (m, 18H, CH_2_ linker), 0.67–0.63 (m, 3H, CH_3_ linker). ^13^C NMR (125 MHz, D_2_O) *δ*: 101.40 (C1″), 92.73 (C1′), 81.88, 81.84, 76.71 (C5″), 75.85 (C5′), 73.87, 73.19, 68.56, 64.81, 63.23, 59.26, 54.79, 49.80, 48.47, 47.31, 38.53 (C6′), 31.28 (CH_2_ linker), 29.47, 28.94, 28.88, 28.86, 28.80 (CH_2_ linker), 28.60, 28.11 (C3′), 27.74 (C2), 25.31 (CH_2_ linker), 22.13 (CH_2_ linker), 13.52 (CH_3_ linker). MALDI TOF-MS *m/e* calcd for C_30_H_61_N_5_O_9_Na, 658.44; measured *m/e*, 658.47 [M + Na]^+^.

#### 4.2.21. *N*-Dodecyl-2-(3-Hydroxy-4-Oxo-1,4-Dihydropyridin-1-Yl) Acetamide (**3**)

A clean, oven-dried RBF was charged with compound **7** (0.105 g, 0.522 mmol) and DCM (10 mL), which were stirred at RT. Then, HATU (0.198 g, 0.522 mmol) and Et_3_N (0.073 mL, 0.522 mmol) were added. After 5 min, dodecylamine (0.048 g, 0.261 mmol) was added to the mixture and the reaction mixture was continuously stirred for 1 h at RT. After completion, the reaction mixture was diluted with DCM (50 mL) and washed with water (30 mL). The organic layer was then washed with a saturated aqueous solution of sodium bicarbonate (30 mL), followed by brine (30 mL), dried over anhydrous Na_2_SO_4_, and concentrated under reduced pressure. The crude mixture was purified using flash chromatography (5% *v/v* MeOH/DCM) to produce **3** (0.137 g, 75%) as a white solid. ^1^H NMR (500 MHz, MeOD) *δ*: 7.53 (d, *J* = 7.5 Hz, 1H, H-5*_def_*), 6.38 (d, *J* = 6.7 Hz, 1H, H-6*_def_*), 4.74 (s, 2H, H-2′*_def_*), 3.22 (t, *J* = 7.0 Hz, 2H, *N*-CH_2_ linker), 2.30 (s, 3H, CH_3*def*_), 1.55–1.49 (m, 2H, CH_2_ linker), 1.33–1.28 (m, 18H, CH_2_ linker), 0.89 (t, *J* = 6.8 Hz, 3H, CH_3_ linker). ^13^C NMR (125 MHz, MeOD) *δ*: 169.87, 167.00, 145.53, 138.99, 131.79, 111.04, 55.48, 39.26, 31.64, 29.34, 29.31, 29.27, 29.25, 29.04, 28.95, 28.90, 26.54, 22.30, 13.00, 10.59. MALDI TOF-MS *m/e* calcd for C_8_H_9_NO_4_, 183.16; measured *m/e*, 184.21 [M + Na]^+^.

NMR spectra (^1^H and ^13^C) for all compounds is provided in Figures S2–S22 in Supplementary Materials. 

### 4.3. Microbiology

#### 4.3.1. Antibacterial Susceptibility Assay

This study was performed using conjugates **1a**–**c** and control compounds **2** and **3** against GNB using the broth microdilution method according to the guidelines of the Clinical and Laboratory Standards Institute (CLSI) [37]. The bacterial isolates used in this study were obtained from the American Type Culture Collection (ATCC) (reference strains), the Canadian National Intensive Care Unit (CAN-ICU) surveillance study [44], and the Canadian Ward (CANWARD) surveillance study [45]. Clinical isolates belonging to the CAN-ICU and CANWARD surveillance studies were recovered from patients suffering presumed infectious diseases entering or admitted to a participating medical center across Canada during the time of the study. The bacterial solution was prepared using overnight-grown bacterial culture diluted in saline to obtain 0.5 McFarland turbidity. Later, this solution was diluted 1:50 in CAMHB. The testing was conducted on a 96-well plate by diluting (2-fold) the hybrids in CAMHB and incubating with equal volumes of inoculum at 37 °C for 18 h. The MIC was determined as the lowest concentration of the hybrid required to inhibit the growth of bacteria [24]. A no-growth ‘well’ was noted visibly, as well as with the use of an EMax Plus microplate reader (Molecular Devices, Sunnyvale, CA, USA) [24]. Iron-depleted CAMHB was prepared under standard conditions, as previously described [46].

#### 4.3.2. Checkerboard Assay

The checkerboard assay was carried out in 96-well plates. The adjuvants under study were diluted along the ordinate, while the antibiotics were diluted along the abscissa. The plates were incubated with equal volumes of inoculum, prepared similarly as discussed above, at 37 °C for 18 h. The growth pattern was then observed using an EMax Plus microplate reader (Molecular Devices, Sunnyvale, CA, USA). The MICs were determined as the lowest concentrations of drugs responsible for inhibition of growth [30]. Successively, FICIs were calculated for the antibiotic combinations using the formula:

ΣFIC=FIC of agent A+FIC of agent B

$$\mathrm{FIC}\;\mathrm{of}\;\mathrm{agent}\;A=\frac{MIC\;of\;agent\;A\;in\;combination}{MIC\;of\;agent\;A\;alone}$$

$$\mathrm{FIC}\;\mathrm{of}\;\mathrm{agent}\;B=\frac{MIC\;of\;agent\;B\;in\;combination}{MIC\;of\;agent\;B\;alone}$$

FICIs of ≤0.5, 0.5 < x ≤4, and >4 correlated to synergistic, additive, and antagonistic interactions of the antibiotic combination, respectively [36]. ID-CAMHB was prepared from CAMHB as reported previously [46]. The determination of the MICs was performed in biological duplicates. If the values were not within 2-fold in agreement, then the assay was repeated.

### 4.4. Cell Viability Assay

#### Toxicity against HEK293 and HepG2 Cells

Human embryonic kidney (HEK293) and human hepatoma (HepG2) cells were grown in Dulbecco’s modified Eagle’s medium (DMEM) supplemented with 10% fetal bovine serum (FBS) in a humidified 5% atmospheric CO_2_ incubator at 37 °C. Equal numbers of cells in 50 µL media (5000-HEK293 and 8000-HepG2) were plated in designated wells in a 96-well plate. The wells with only media and no cells served as blanks. After 24 h of incubation, experimental wells with cells and corresponding blank wells were incubated with drugs in 50 µL to the desired concentration (0–200 µM) for 48 h. To assess the cell viability, PrestoBlue reagent from Invitrogen was added to the wells to a final concentration of 10% (*v/v*), and plates were incubated in the CO_2_ incubator for an additional 1 h. Subsequently, the fluorescence was measured with excitation and emission wavelengths of 560 and 590 nm, respectively, using the SpectraMax M2 plate reader from Molecular Devices. The cell viability was interpreted as previously stated [47]. The values from blank wells were subtracted from those from the corresponding wells with cells. Finally, the cell viability was calculated relative to that of the controls with vehicle. The data were plotted as line graphs, and the plots indicated the means ± standard deviations of two individual experiments, with five wells with cells dedicated to each concentration.

## 5. Conclusions

TOB-DEF conjugates form a new class of selective tetracycline antibiotic potentiators against *P. aeruginosa.* The potentiating effect of conjugate **1c** required a hydrophobic tether and the presence of the Fe^3+^ chelator DEF in order to achieve optimal tetracycline potentiation. Similar tether effects were previously observed with related amphiphilic tobramycin conjugates that display considerable cytotoxicity once the carbon tether reaches a length of C_8_ or higher [30,31,32,33]. In addition, the reasons why DEF was required remain unclear, as Fe^3+^-depleted conditions in the bacterial media had little consequence and mostly showed no improvement in the antibacterial activity or antibiotic potentiation. Conjugate **1c** (8.5 μM) is believed to perturb the LPS layer of *P. aeruginosa*’s OM, leading to a transient destabilization of the OM that increases the permeability of tetracycline antibiotics, as seen for other polybasic amphiphiles [30,41,42]. This conclusion is supported by the fact that tetracycline potentiation of **1c** was abolished in the presence of elevated Mg^2+^ concentrations (20 mM), as Mg^2+^ competes with the binding of **1c** to LPS. Collectively, our data suggest that the observed synergy of conjugate **1c** with tetracyclines is independent of the Fe^3+^-complexing properties of the molecule and is the result of enhanced OM permeability in *P. aeruginosa*. In addition, the reasons for the observed selectivity of tetracycline potentiation of **1c** when compared with PMBN remain unclear.

## Figures and Tables

**Table 1:** Interactions of compound **1c** (8.5 μM) with tetracyclines against clinical isolates of *P. aeruginosa* in ID-CAMHB and CAMHB.

Strain	Antibiotic	MIC (μg/mL) of Antibiotic			
		Alone	+ Compound 1c	Alone	+ Compound 1c
		ID-CAMHB		CAMHB	
PAO1	MIN	32	8	32	1
	DOX	16	0.25	64	2
	TIG	64	2	32	4
	ERV	8	2	8	8
PA259	MIN	32	16	128	2
	DOX	64	2	128	4
	TIG	64	2	64	1
	ERV	16	4	16	2
PA262	MIN	128	16	128	8
	DOX	256	32	256	16
	TIG	64	16	64	16
	ERV	16	8	16	16
PA264	MIN	64	4	64	2
	DOX	64	16	32	0.25
	TIG	64	4	64	1
	ERV	16	16	16	4

Synergistic combinations are highlighted in green.

**Table 2:** Comparative potentiation of tetracyclines by compound **1c** against *P. aeruginosa* in CAMHB and Mg^2+^-supplemented CAMHB.

Strain	Antibiotic	Concentration of 1c (μg/mL)	MIC (μg/mL) of Antibiotic			
			Alone	+ Compound 1c	Alone	+ Compound 1c
			CAMHB		Mg^2+^ Supplemented CAMHB	
PAO1	MIN	8	32	1	>512	>512
		16	32	1	>512	>512
		32	32	1	>512	>512
		64	32	0.5	>512	128
	DOX	8	64	2	512	512
		16	64	2	512	256
		32	64	1	512	256
		64	64	0.5	512	128
PA259	MIN	8	128	2	>512	>512
		16	128	2	>512	>512
		32	128	2	>512	>512
		64	128	1	>512	128
	DOX	8	128	4	512	512
		16	128	2	512	256
		32	128	2	512	256
		64	128	2	512	128

Synergistic combinations are highlighted in green.

## Data Availability

The data presented in this study are available within the article and the Supplementary Material.

## Supplementary Materials

The following supporting information can be downloaded at: https://www.mdpi.com/article/10.3390/antibiotics12081261/s1, Table S1: Antibacterial activity of compounds **1a**–**c**, **2**, and **3** against several strains of Gram-negative bacteria; Table S2: Combination studies of compounds **1a**–**c** and PMBN with different antibiotics against *P. aeruginosa* PAO1; Table S3: Resistance phenotype of *P. aeruginosa* clinical isolates; Table S4. Interactions of conjugate **1c** (8.5 μM) with minocycline (MIN), doxycycline (DOX), tigecycline (TIG), and eravacycline (ERV) against clinical isolates of *P. aeruginosa*; Table S5: Interaction of compound **2** (8.5 μM) and select antibiotics against *P. aeruginosa* PAO1 and PA259; Table S6: Interaction of compound **3** (8.5 μM) and select antibiotics against *P. aeruginosa* PAO1 and PA259; Table S7: Antibacterial activity of cefiderocol against *P. aeruginosa* in CAMHB and ID-CAMHB; Figure S1: Cytotoxicity data for compounds **1a**–**c**; Figure S2: ^1^H and ^13^C NMR spectra of compound **1a** in D_2_O; Figure S3: COSY and HSQC NMR spectra of compound **1a** in D_2_O; Figure S4: HMBC NMR spectrum of compound **1a** in D_2_O; Figure S5: ^1^H and ^13^C NMR spectra of compound **1b** in D_2_O; Figure S6: COSY and HSQC NMR spectra of compound **1b** in D_2_O; Figure S7: HMBC NMR spectrum of compound **1b** in D_2_O; Figure S8: ^1^H and ^13^C NMR spectra of compound **1c** in D_2_O; Figure S9: COSY and HSQC NMR spectra of compound **1c** in D_2_O; Figure S10: HMBC NMR spectrum of compound **1c** in D_2_O; Figure S11: ^1^H and ^13^C NMR spectra of compound **2** in D_2_O; Figure S12: COSY and HSQC NMR spectra of compound **2** in D_2_O; Figure S13: HMBC NMR spectrum of compound **2** in D_2_O; Figure S14: ^1^H and ^13^C NMR spectra of compound **3** in D_2_O; Figure S15: ^1^H NMR spectra of compound **5a** and **5b** in CDCl_3_; Figure S16: ^1^H NMR spectra of compound **5c** and **6a** in CDCl_3_; Figure S17: ^1^H NMR spectra of compound **6b** and **6c** in CDCl_3_; Figure S18: ^1^H and ^13^C NMR spectra of compound **7** in D_2_O; Figure S19: ^1^H and ^13^C NMR spectra of compound **8a** in CDCl_3_; Figure S20: ^1^H and ^13^C NMR spectra of compound **8b** in CDCl_3_; Figure S21: ^1^H and ^13^C NMR spectra of compound **8c** in CDCl_3_; Figure S22: ^1^H NMR spectrum of compound **9** in CDCl_3_; HPLC chromatograms of compounds **1a**–**c**.
