# Supplementary material for: Exploring Antibiotic-Potentiating Effects of Tobramycin–Deferiprone Conjugates in Pseudomonas aeruginosa
Source: Antibiotics (Basel). 2023 Jul 31;12(8):1261. doi: 10.3390/antibiotics12081261 (PMC10451322; doi:10.3390/antibiotics12081261)
Supplement: Supplementary file 1 [file antibiotics-12-01261-s001.zip › antibiotics-2529345-supplementary.pdf]

Supplementary Materilas

Exploring Antibiotic-Potentiating Effects of Tobramycin–Deferiprone Conjugates in *Pseudomonas aeruginosa*

Karan Gandhi [1], Shiv Dhiman [1], Rajat Arora [1], Danzel Marie Ramirez [1], Danyel Ramirez [1], Gilbert Arthur [2] and Frank Schweizer [1,3,*]

[1] Department of Chemistry, Faculty of Science, University of Manitoba, Winnipeg, MB R3T 2N2, Canada; gandhikaran2@gmail.com (K.G.); shiv.dhiman@umanitoba.ca (S.D.); arorar5@myumanitoba.ca (R.A.); ramirezd@myumanitoba.ca (D.M.R.); ramiredm@myumanitoba.ca (D.R.)

[2] Department of Biochemistry and Medical Genetics, University of Manitoba, Winnipeg, MB R3E 0J9, Canada; gilbert.arthur@umanitoba.ca

[3] Department of Medical Microbiology and Infectious Diseases, University of Manitoba, Winnipeg, MB R3R 0J9, Canada

* Correspondence: frank.schweizer@umanitoba.ca

Table of Contents

1. Table S1: Antibacterial activity (μg/mL) of compounds **1a-c**, **2**, and **3** against several strains of Gram-negative bacteria…………………………………………………………….....3
2. Table S2: Combination studies of compounds **1a-c** and PMBN with different antibiotics against *P. aeruginosa* PAO1……………..……………………………………………….....4
3. Table S3: Resistance phenotype of *P. aeruginosa* clinical isolates……………………..…4
4. Table S4: Interactions of conjugate **1c** (8.5 μM) with minocycline (MIN), doxycycline (DOX), tigecycline (TIG), and eravacycline (ERV) against clinical isolates of *P. aeruginosa*……..………………………………………………………………………….....5
5. Table S5: Interaction of compound **2** (8.5 μM) and select antibiotics against *P. aeruginosa* PAO1 and PA259…………………………………………………………………......…6
6. Table S6: Interaction of compound **3** (8.5 μM) and select antibiotics against *P. aeruginosa* PAO1 and PA259…………………………………………………………………......…6
7. Table S7: Antibacterial activity of Cefiderocol against *P. aeruginosa* in CAMHB and ID-CAMHB conditions. ……………………………………………………………………………7
8. Figure S1: Cytotoxicity data for compounds **1a-c**………………………….……….…......8
9. HPLC chromatograms of compound **1a-c**……………………………………………..9-14
10. Figures S2 – S22: NMR spectrum of compounds **1-9**………………………………..15-35

Table S1: Antibacterial activity of compounds **1a-c**, **2**, and **3** against several strains of Gram-negative bacteria

**Test organism**	**Minimum Inhibitory Concentration (μg/mL)**				
	**1a**	**1b**	**1c**	**2**	**3**
P. aeruginosa PAO1	>128	>128	>128	>128	>128
P. aeruginosa PA259	>128	>128	>128	>128	>128
P. aeruginosa PA262	>128	>128	>128	ND	ND
P. aeruginosa PA264	>128	>128	>128	ND	ND
P. aeruginosa PA083	>128	>128	>128	ND	ND
P. aeruginosa PA095	>128	>128	>128	ND	ND
E. coli ATCC 25922	>128	>128	>128	ND	ND
A. baumannii ATCC 17978	>128	>128	>128	ND	ND

ND = Not determined

Table S2: Combination studies of compounds **1a-c** (8.5 μM, (8 μg/mL)) and PMBN (7.0 μM, (8 μg/mL)) with different antibiotics against *P. aeruginosa* PAO1. MICs are reported in μg/mL. FICI = Fractional inhibitory concentration index. FICI of ≤0.5, 0.5<x≤4, and >4 indicate synergy, additive or no interaction, and antagonism, respectively. Synergistic combinations are highlighted in green.

**Antibiotics (MIC alone)**	**MIC of antibiotics (FICI) in the combination**			
	**+ 1a**	**+ 1b**	**+ 1c**	**+ PMBN**
Novobiocin (1024)	256 (0.25<x<0.31)	256 (0.25<x<0.31)	128 (0.12<x<0.18)	4 (0.003<x<0.066)
Rifampicin (32)	8 (0.25<x<0.31)	32 (1<x<1.002)	4 (0.12<x<0.14)	0.5 (0.015<x<0.078)
Levofloxacin (0.5)	0.5 (1<x<1.002)	0.25 (0.5<x<0.508)	0.25 (0.5<x<0.503)	NT
Minocycline (32)	8 (0.25<x<0.28)	8 (0.25<x<0.31)	1 (0.03<x<0.06)	0.25 (0.008<x<0.070)
Doxycycline (64)	4 (0.25<x<0.31)	1 (0.06<x<0.08)	2 (0.03<x<0.6)	0.25 (0.004<x<0.066)
Tigecycline (64)	16 (0.25<x<0.31)	8 (0.125<x<0.13)	2 (0.03<x<0.06)	NT
Eravacycline (8)	4 (0.5<x<0.56)	2 (0.25<x<0.31)	2 (0.25<x<0.28)	NT
Ceftazidime (2)	1 (0.5<x<0.53)	1 (0.5<x<0.53)	1 (0.5<x<0.501)	0.125 (0.062<x<0.125)
Aztreonam (4)	4 (1<x<1.004)	4 (1<x<1.1)	1 (0.25<x<0.28)	0.25 (0.062<x<0.125)
Meropenem (1)	1 (1<x<1.002)	1 (1<x<1.002)	1 (1<x<1.002)	NT
Imipenem (2)	2 (1<x<1.002)	2 (1<x<1.002)	1 (0.5<x<0.53)	NT

NT = not tested

Table S3: Resistance phenotype of *P. aeruginosa* clinical isolates.

***P. aeruginosa* isolates**	**NOV**	**RIF**	**LEV**	**MIN**	**DOX**	**TIG**	**ERV**	**CAZ**	**AZT**	**MER**	**IMI**
**PA259-96916**	1024	16	512	128	128	64	16	256	32	512	64
**PA262-101856**	1024		128	128	256	64	16	16	32	64	64
**PA264-104354**	1024	32	64	64	32	64	16	64	64	64	32
**PA095**	ND	ND	ND	32	16	16	16	16	ND	16	4

NOV: novobiocin; RIF: rifampicin; LEV: levofloxacin; MIN: minocycline; DOX: doxycycline; TIG: tigecycline; ERV: eravacycline; CAZ: ceftazidime; AZT: aztreonam; MER: meropenem; IMI: imipenem

Table S4: Interactions of conjugate **1c** (8.5 μM) with minocycline (MIN), doxycycline (DOX), tigecycline (TIG), and eravacycline (ERV) against clinical isolates of *P. aeruginosa*. MICs are reported in μg/mL. FICI of ≤0.5, 0.5<x≤4, and >4 indicate synergy, additive or no interaction, and antagonism, respectively. Synergistic combinations are highlighted in green.

P. aeruginosa **Isolates**	**Antibiotic**	**MIC (μg/mL) of antibiotic**		**Fold Potentiation**	**FICI**
		Alone	+ Compound **1c**		
PA259	MIN	128	2	64	0.01<x<0.07
	DOX	128	4	32	0.03<x<0.09
	TIG	64	1	64	0.02<x<0.04
	ERV	16	2	8	0.13<x<0.16
PA262	MIN	128	8	16	0.06<x<0.09
	DOX	256	16	16	0.06<x<0.08
	TIG	64	16	4	0.25<x<0.27
	ERV	16	16	1	0.5<x<0.50
PA264	MIN	64	2	32	0.03<x<0.06
	DOX	32	0.25	128	0.01<x<0.04
	TIG	64	1	64	0.02<x<0.08
	ERV	16	4	4	0.25<x<0.28
PA095	MIN	32	0.5	64	0.02<x<0.03
	DOX	16	0.125	128	0.01<x<0.04
	TIG	16	2	8	0.13<x<0.14
	ERV	16	0.5	32	0.03<x<0.05

Table S5: Interaction of compound **2** (8.5 μM) and select antibiotics against *P. aeruginosa* PAO1 and PA259. MICs are reported in μg/mL.

Antibiotic	*P. aeruginosa* isolates	$MIC_{adjuvant}$ [MIC_{combo}]	$MIC_{antibiotic}$ [MIC_{combo}]	FIC index	Interpretation	Abs. MIC	Fold potentiation
MIN	PAO1	>128 [2]	32 [2]	0.0625-0.078	Synergy	2	16
	PA259	>128 [4]	64 [16]	0.25-0.281	Synergy	16	4
DOX	PAO1	>128 [1]	64 [16]	0.25<x<0.258	Synergy	16	4
	PA259	>128 [2]	64 [8]	0.125<x<0.140	Synergy	8	8
TIG	PAO1	>128 [0.5]	64 [32]	0.5<x<0.504	Additive	32	2
	PA259	>128 [4]	64 [16]	0.25<x<0.281	Synergy	16	4
ERV	PAO1	>128 [4]	8 [4]	0.5<x<0.531	Additive	4	2
	PA259	>128 [8]	16 [2]	0.125<x<0.187	Synergy	2	8

MIN: minocycline; DOX: doxycycline; TIG: tigecycline; ERV: eravacycline

Table S6: Interaction of compound **3** with tetracyclines against *P. aeruginosa* PAO1 and PA259. MICs are reported in μg/mL.

Antibiotic	*P. aeruginosa* isolates	$MIC_{adjuvant}$ [MIC_{combo}]	$MIC_{antibiotic}$ [MIC_{combo}]	FIC Index	Interpretation	Absolute MIC	Fold Potentiation
MIN	PAO1	>128 [4]	32 [16]	0.5<x<0.531	Additive	16	2
	PA259	>128 [0.25]	128 [128]	1<x<1.002	Additive	128	1
DOX	PAO1	>128 [0.25]	64 [64]	1<x<1.002	Additive	64	1
	PA259	>128 [4]	64 [32]	0.5<x<0.531	Additive	32	2
TIG	PAO1	>128 [1]	64 [64]	1<x<1.008	Additive	64	1
	PA259	>128 [0.25]	64 [64]	1<x<1.002	Additive	64	1
ERV	PAO1	>128 [0.5]	8 [8]	1<x<1.003	Additive	8	1
	PA259	>128 [0.25]	16 [16]	1<x<1.002	Additive	16	1

MIN: minocycline; DOX: doxycycline; TIG: tigecycline; ERV: eravacycline

Table S7: Antibacterial activity of cefiderocol and compound **1c** against *P. aeruginosa* in CAMHB and ID-CAMHB.

***P. aeruginosa* isolates**	**CAMHB (µg/mL)**		**ID-CAMHB (µg/mL)**	
	Cefiderocol	**1c**	Cefiderocol	**1c**
**PAO1**	2	>128	0.25	>128
**PA259**	0.25	>128	0.25	>128
**PA262**	0.25	>128	0.25	>128
**PA264**	0.25	>128	0.25	>128

CAMHB = cation-adjusted Mueller-Hinton broth; ID-CAMHB = iron-depleted cation-adjusted Mueller-Hinton broth

Figure S1: Cytotoxicity data for compounds **1a-c** relative to control (vehicle) against HEK293 and HepG2 cell lines with doxorubicin as a positive control and polymyxin B (PMB) as a negative control. Results represent the mean ± standard deviation of two independent experiments with five wells for each concentration.

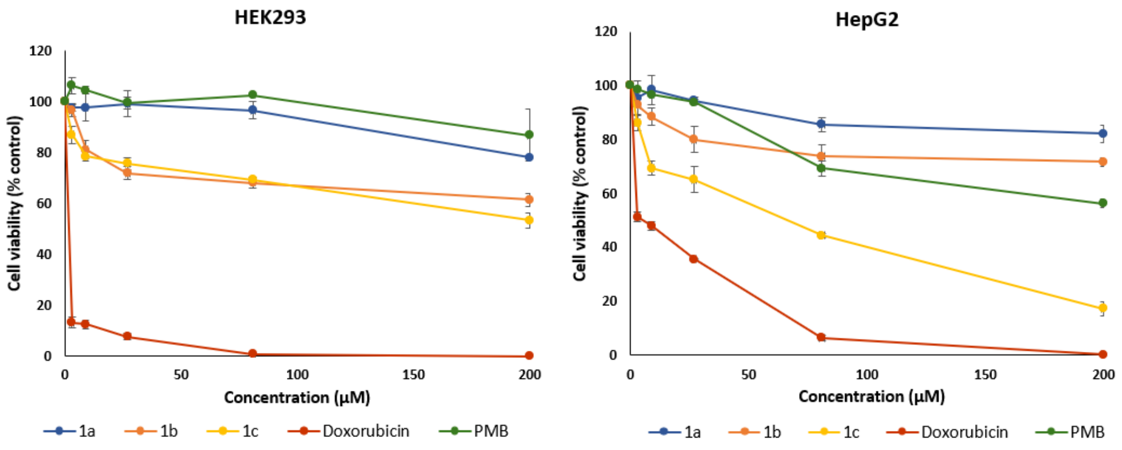

HPLC chromatograms of compound 1a-c

Method A: Synergi 2.5 μM Polar-RP 100 Å LC column (50 mm × 2 mm, Phenomenex)

Buffer A: 0.1% TFA in water; Buffer B: 0.1% TFA in acetonitrile

Flow rate: 0.15 ml/min; run time: 9 min; UV-Visible detection at 275 nm

Time duration (min)	% Buffer A	% Buffer B
0	99	1
1	99	1
2	95	5
3	95	5
4	75	25
5	75	25
6	50	50
7	50	50
8	99	1
9	99	1

Method B: Synergi 2.5 μM Polar-RP 100 Å LC column (50 mm × 2 mm, Phenomenex)

Buffer A: 0.1% TFA in water; Buffer B: 0.1% TFA in acetonitrile

Flow rate: 0.15 ml/min; run time: 20 min; UV-Visible detection at 275 nm

Time duration (min)	% Buffer A	% Buffer B
0	95	5
3	95	5
4	90	10
6	90	10
7	85	15
9	85	15
10	75	25
13	75	25
14	50	50
15	50	50
18	95	5
20	95	5

Method C: Synergi 2.5 μM Polar-RP 100 Å LC column (50 mm × 2 mm, Phenomenex)

Buffer A: 0.1% TFA in water; Buffer B: 0.1% TFA in acetonitrile

Flow rate: 0.3 ml/min; run time: 20 min; UV-Visible detection at 275 nm

Time duration (min)	% Buffer A	% Buffer B
0	90	10
3	90	10
4	85	15
6	85	15
7	60	20
9	60	20
10	70	30
13	70	30
14	50	50
15	50	50
18	90	10
20	90	10

Chromatogram and Results			
Sample name:	Compound **1a**	Run time (min):	8.93
Vial number:	R:A3	Injection volume (μl):	3.00
Instrument method:	Method A	Channel name:	UV_VIS_1
Injection date/time:	25/Jun/22 16:36	Wavelength (nm):	275 nm

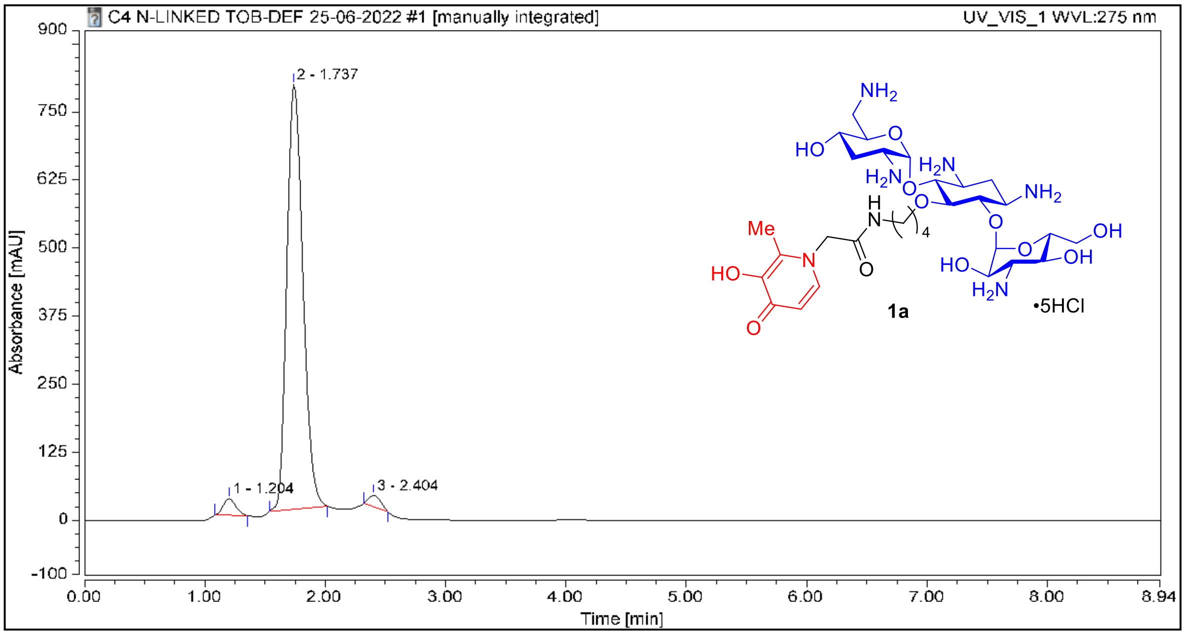

Sr. No.	Retention time (min)	Area (mAU*min)	Height (mAU)	Relative Area (%)
1	1.204	3.651	30.341	2.87
2	1.737	120.955	779.312	95.22
3	2.404	2.427	20.892	1.91

Chromatogram and Results			
Sample name:	Compound **1b**	Run time (min):	20.00
Vial number:	R:A2	Injection volume (μl):	3.00
Instrument method:	Method B	Channel name:	UV_VIS_1
Injection date/time:	25/Jun/22 15:27	Wavelength (nm):	275 nm

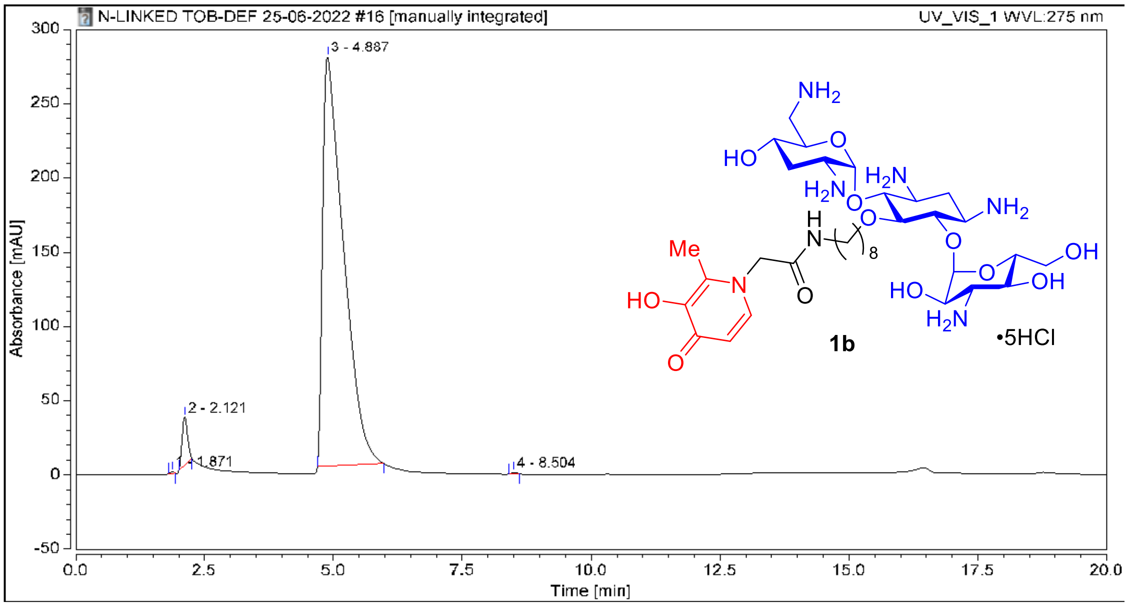

Sr. No.	Retention time (min)	Area (mAU*min)	Height (mAU)	Relative Area (%)
1	1.871	0.081	0.998	0.06
2	2.121	4.060	31.946	3.06
3	4.887	128.319	275.522	96.80
4	8.504	0.106	0.928	0.08

Chromatogram and Results			
Sample name:	Compound **1c**	Run time (min):	20.00
Vial number:	R:A2	Injection volume (µl):	3.00
Instrument method:	Method B	Channel name:	UV_VIS_1
Injection date/time:	25/Jun/22 15:27	Wavelength (nm):	275 nm

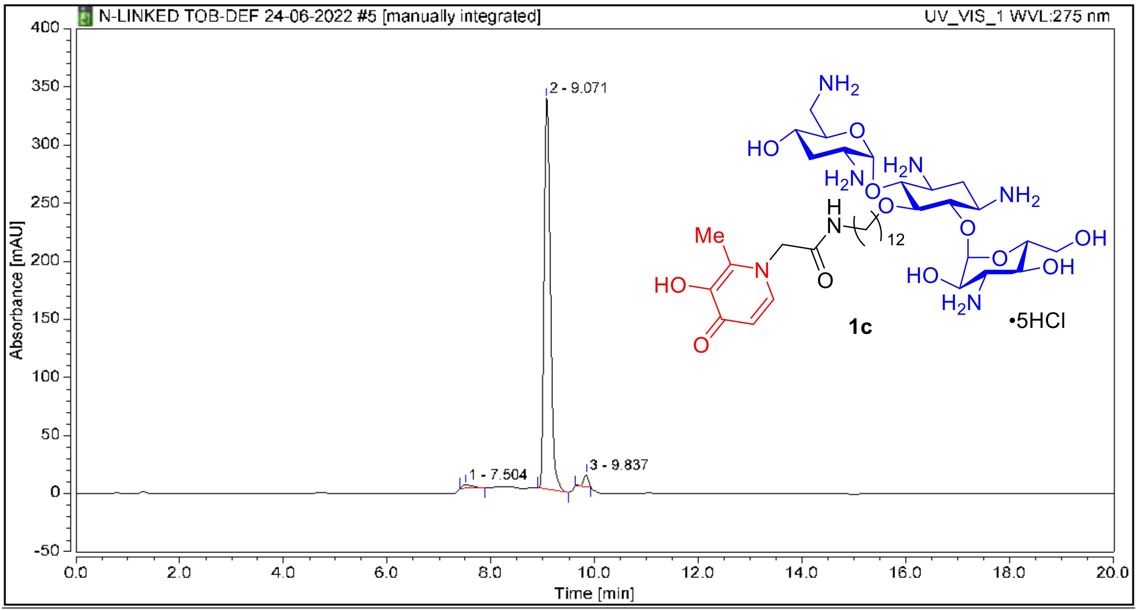

Sr. No.	Retention time (min)	Area (mAU*min)	Height (mAU)	Relative Area (%)
1	7.504	0.680	2.786	1.39
2	9.071	47.056	336.270	96.29
3	9.837	1.134	10.057	2.32

NMR Spectra of compounds **1 – 9**

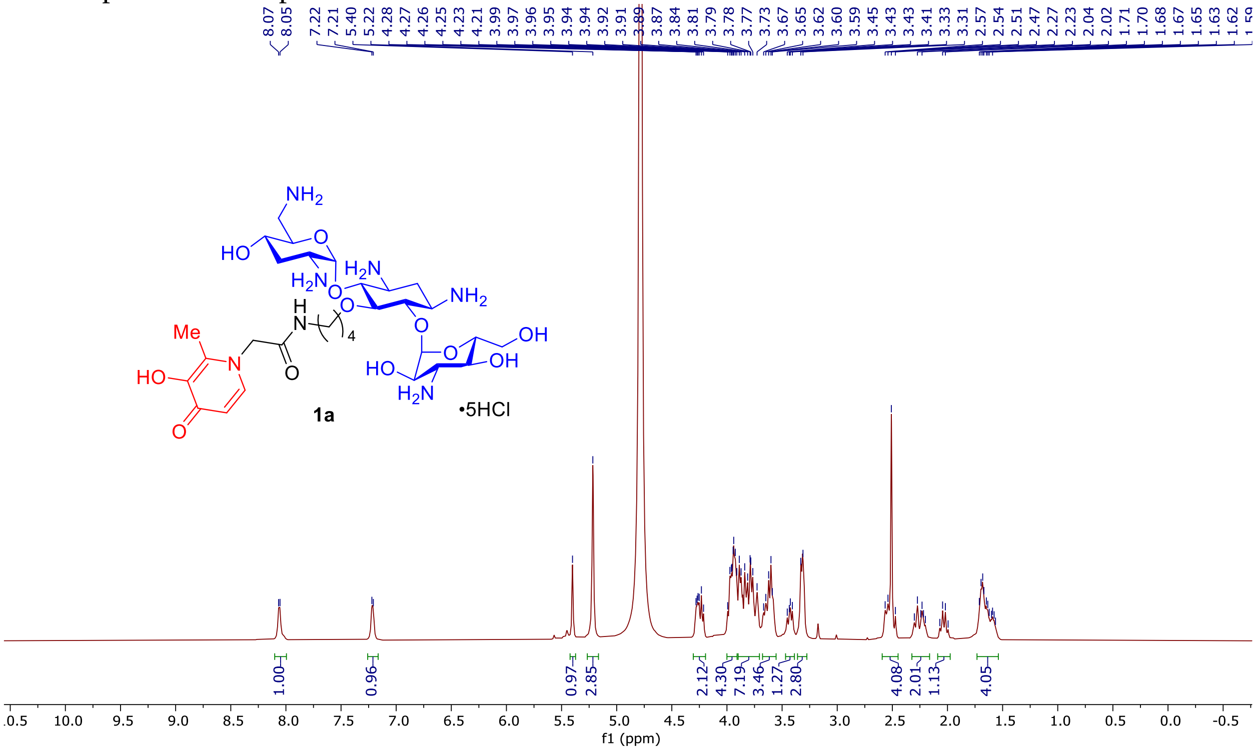

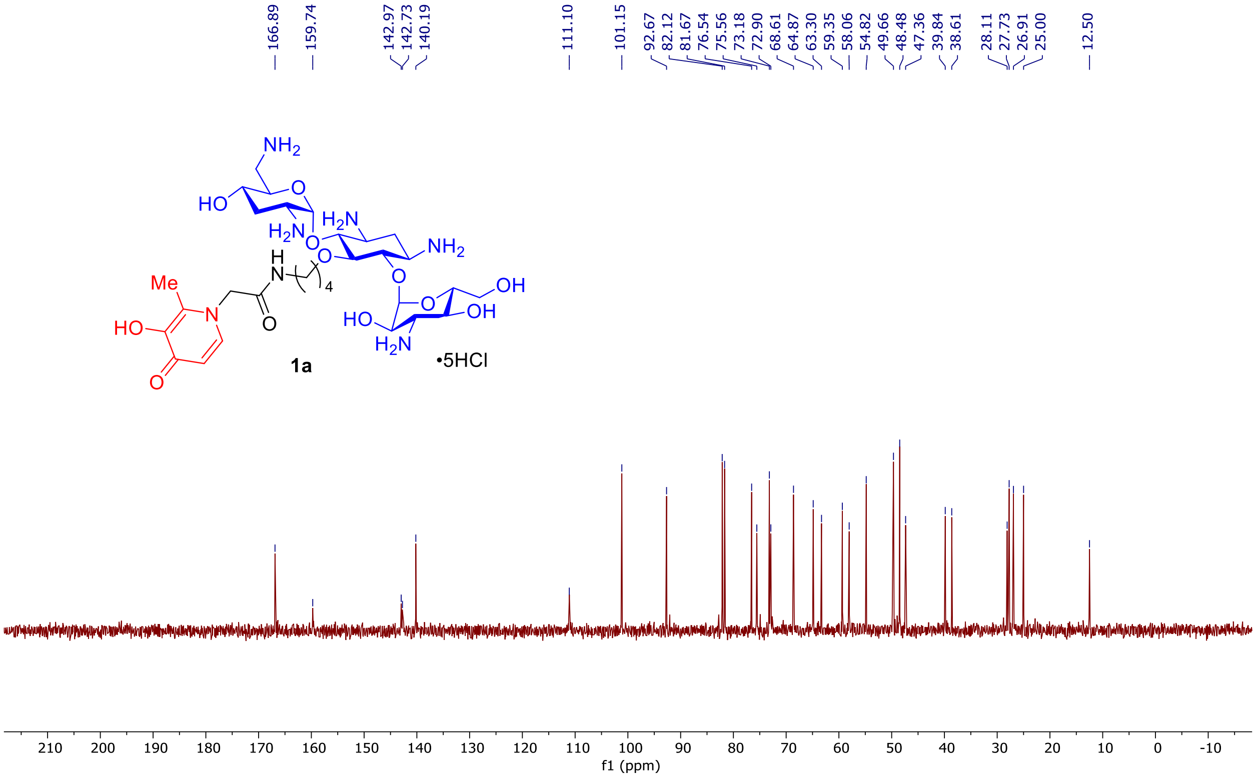

Figure S2: ^{1}H and ^{13}C NMR spectra of compound **1a** in D_2O

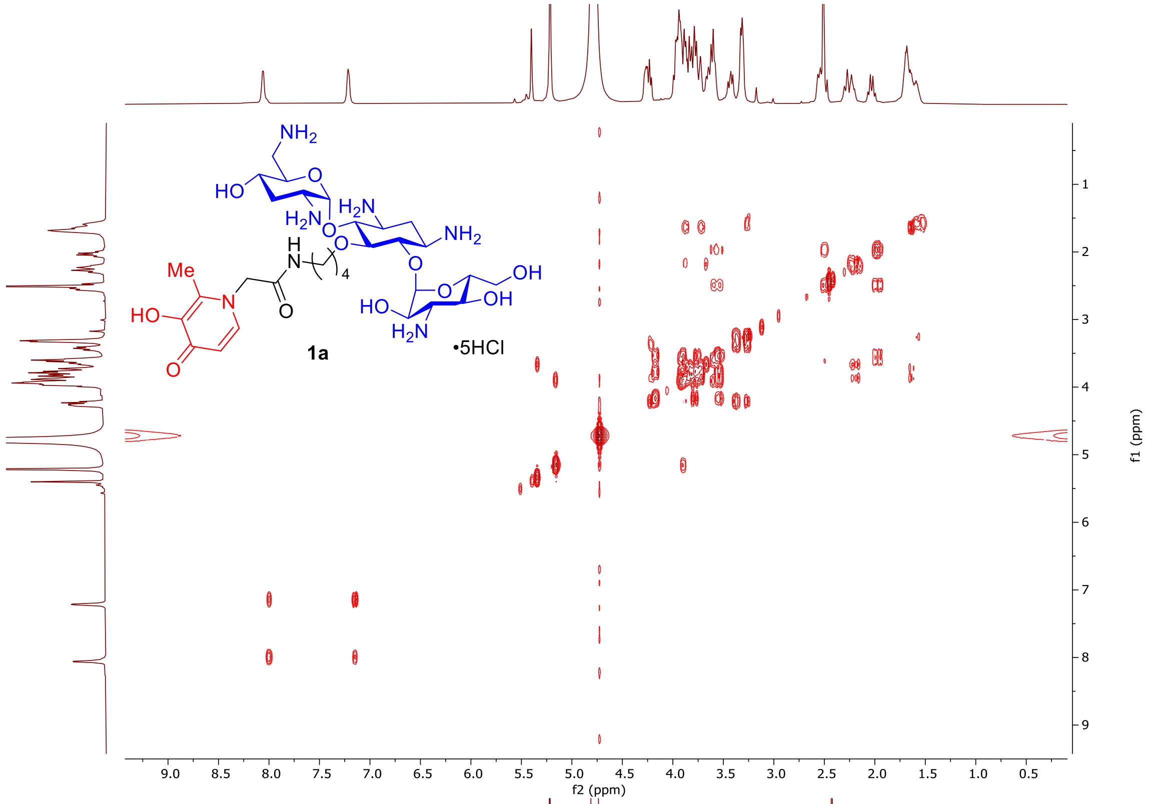

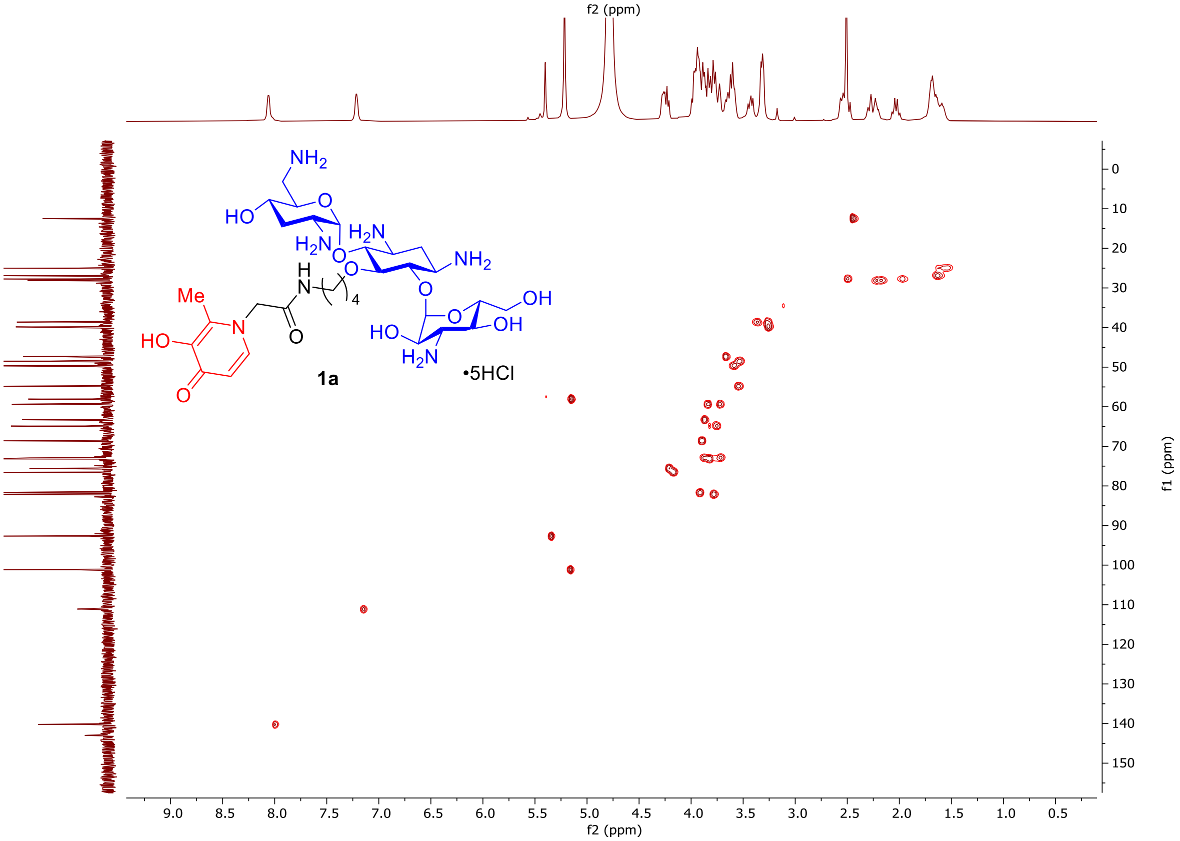

Figure S3: COSY and HSQC NMR spectra of compound **1a** in D_2O

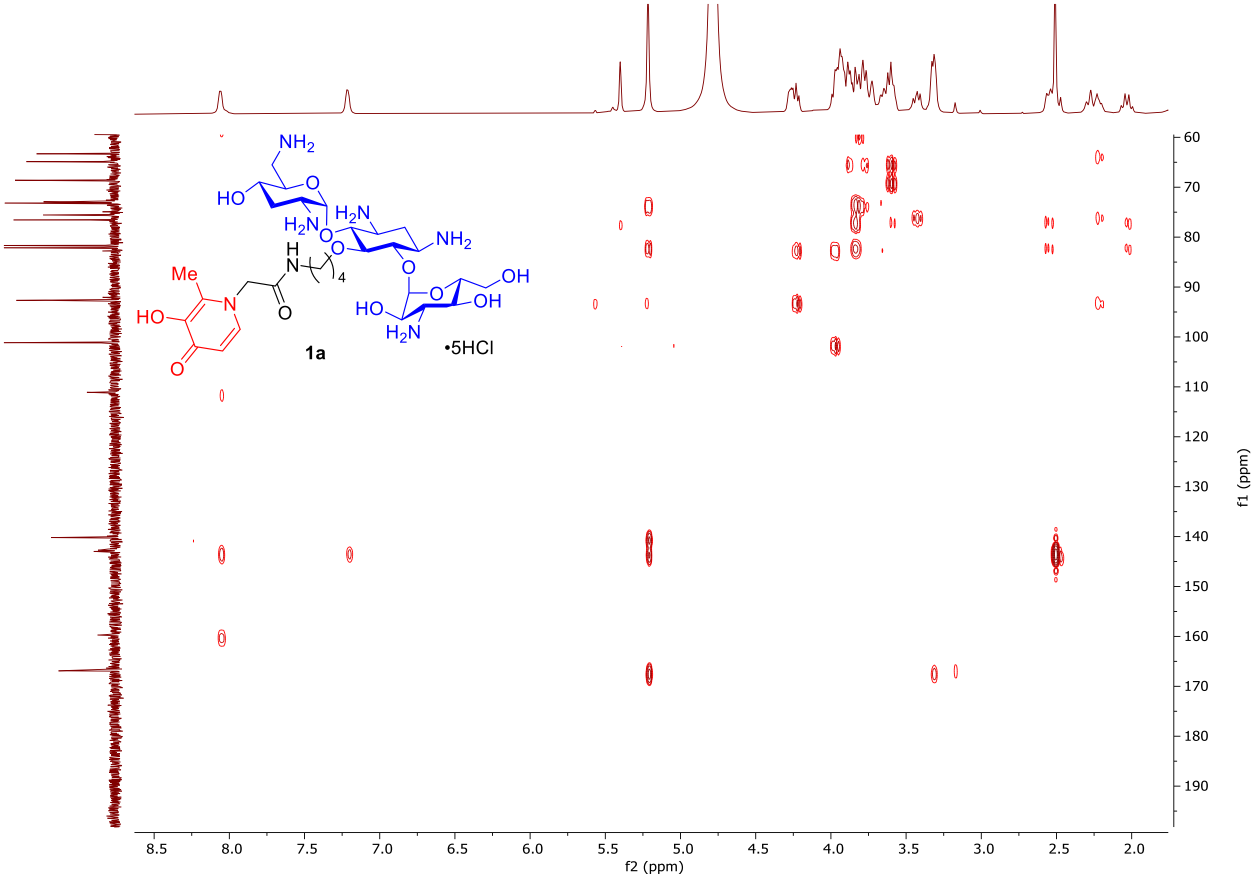

Figure S4: HMBC NMR spectrum of compound **1a** in D_2O

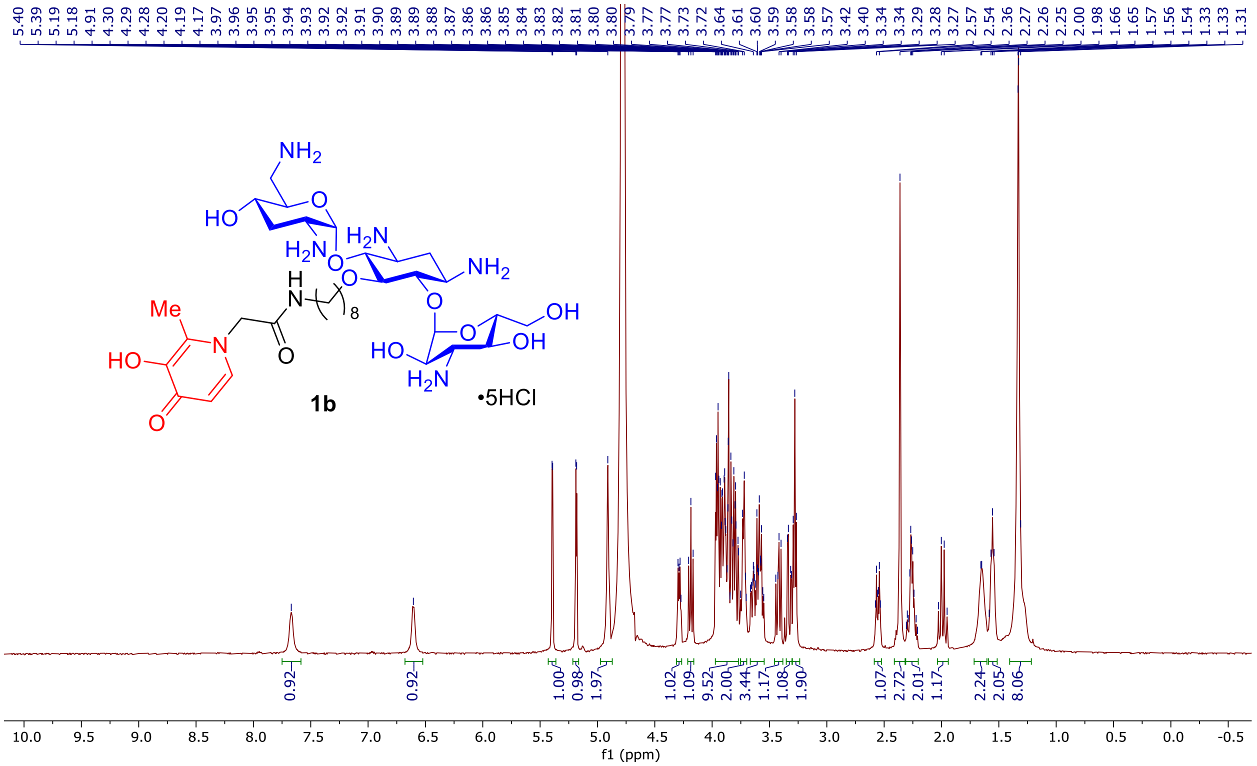

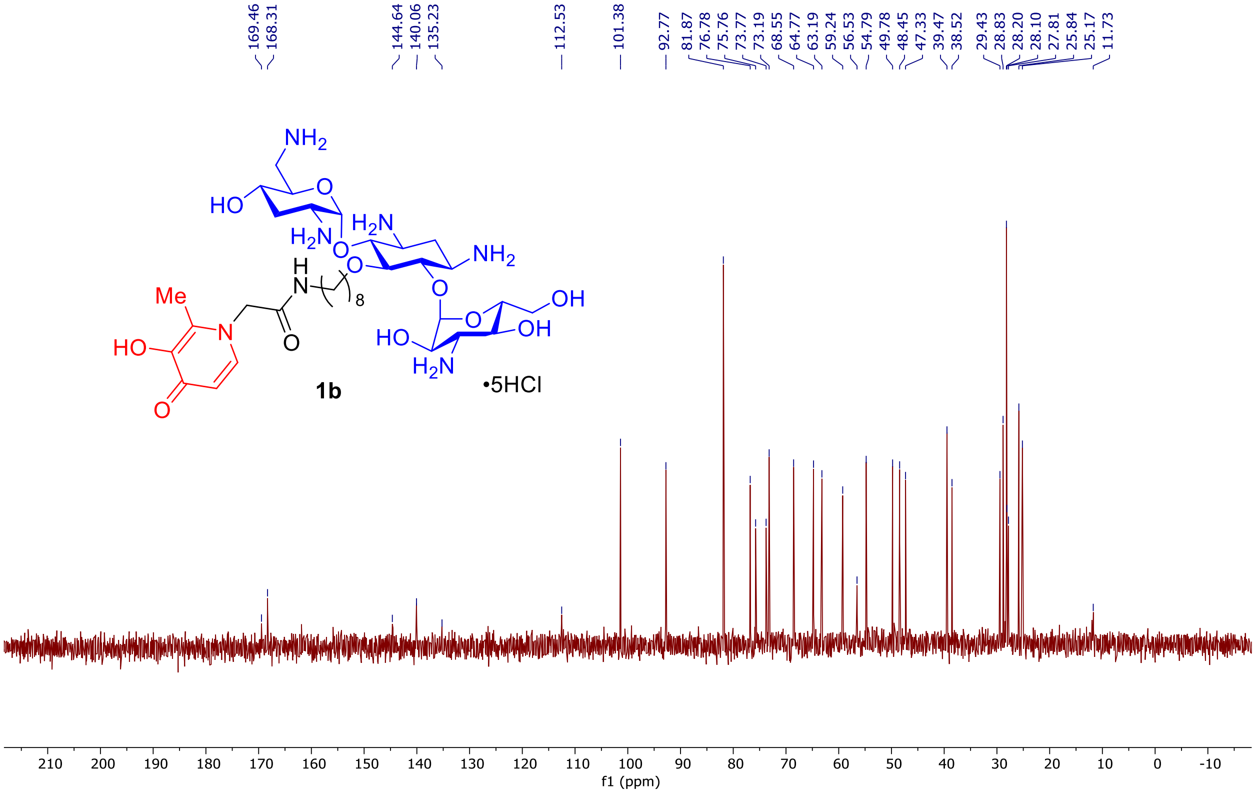

Figure S5: ^{1}H and ^{13}C NMR spectra of compound **1b** in D_2O

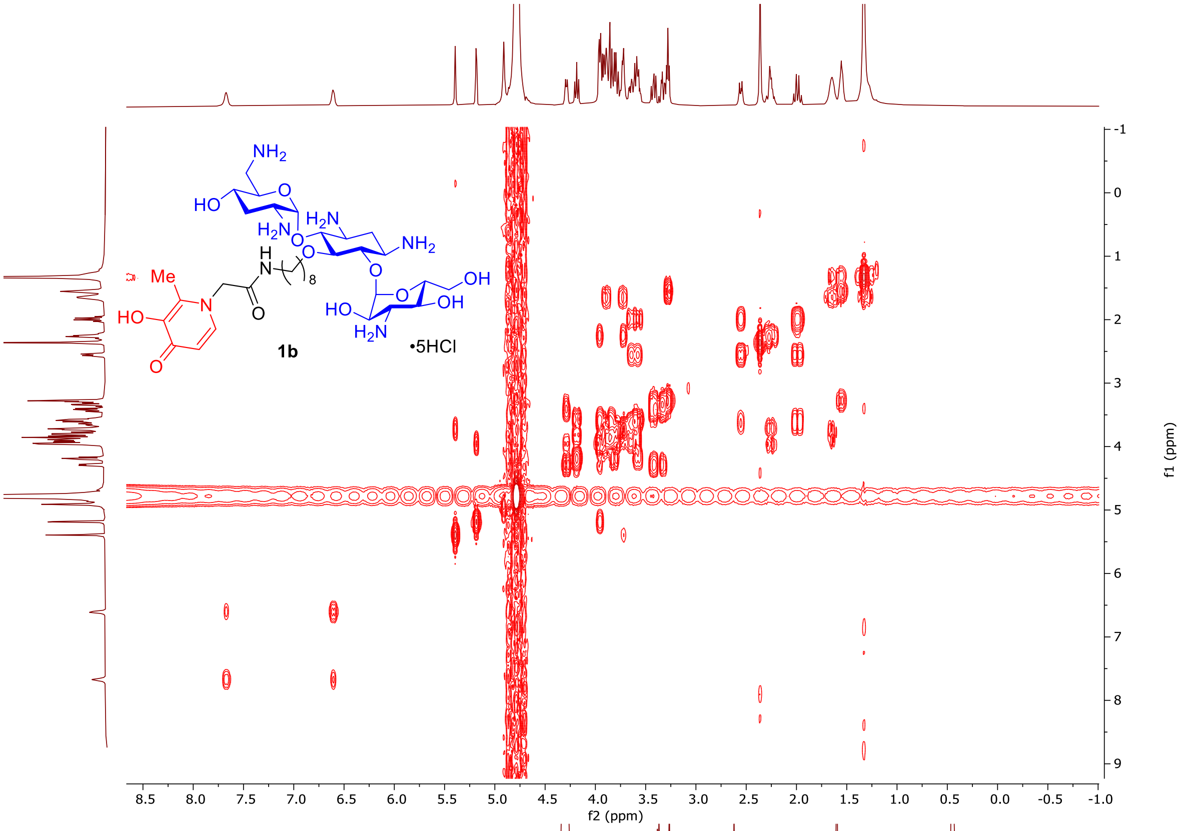

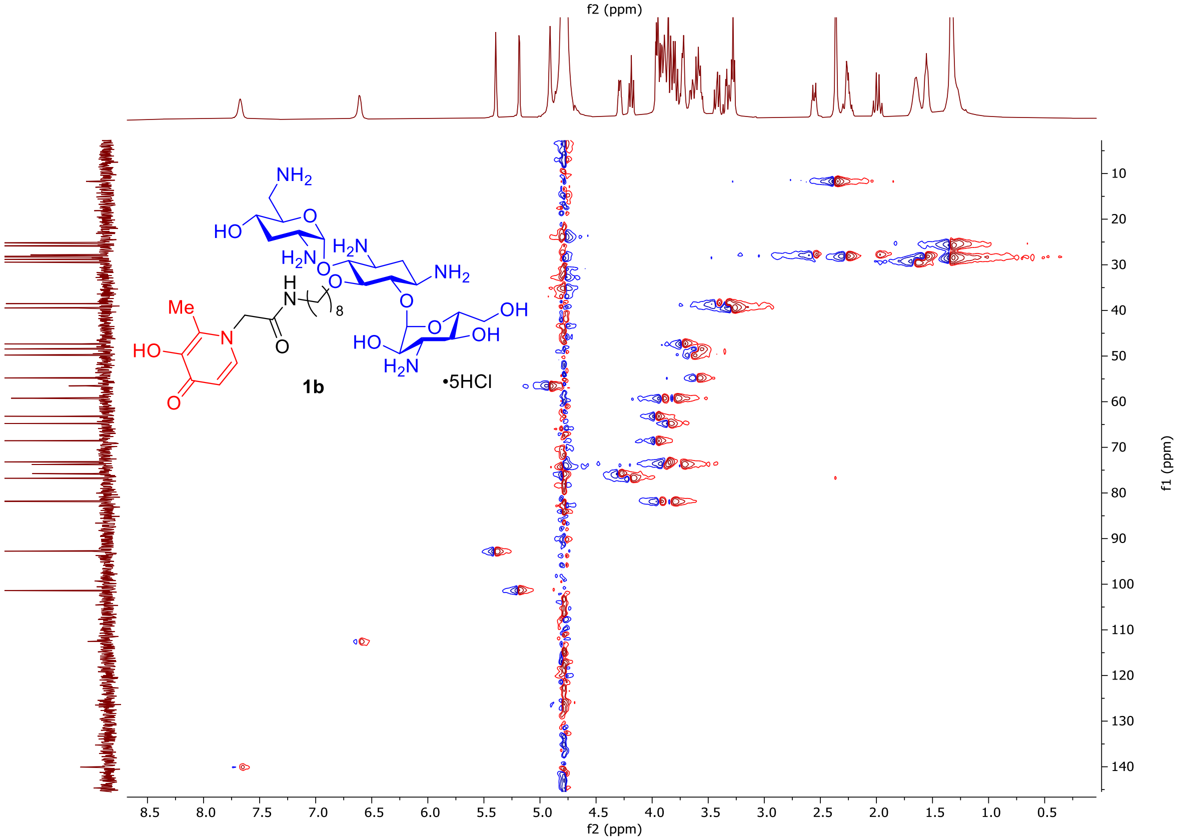

Figure S6: COSY and HSQC NMR spectra of compound **1b** in D_2O

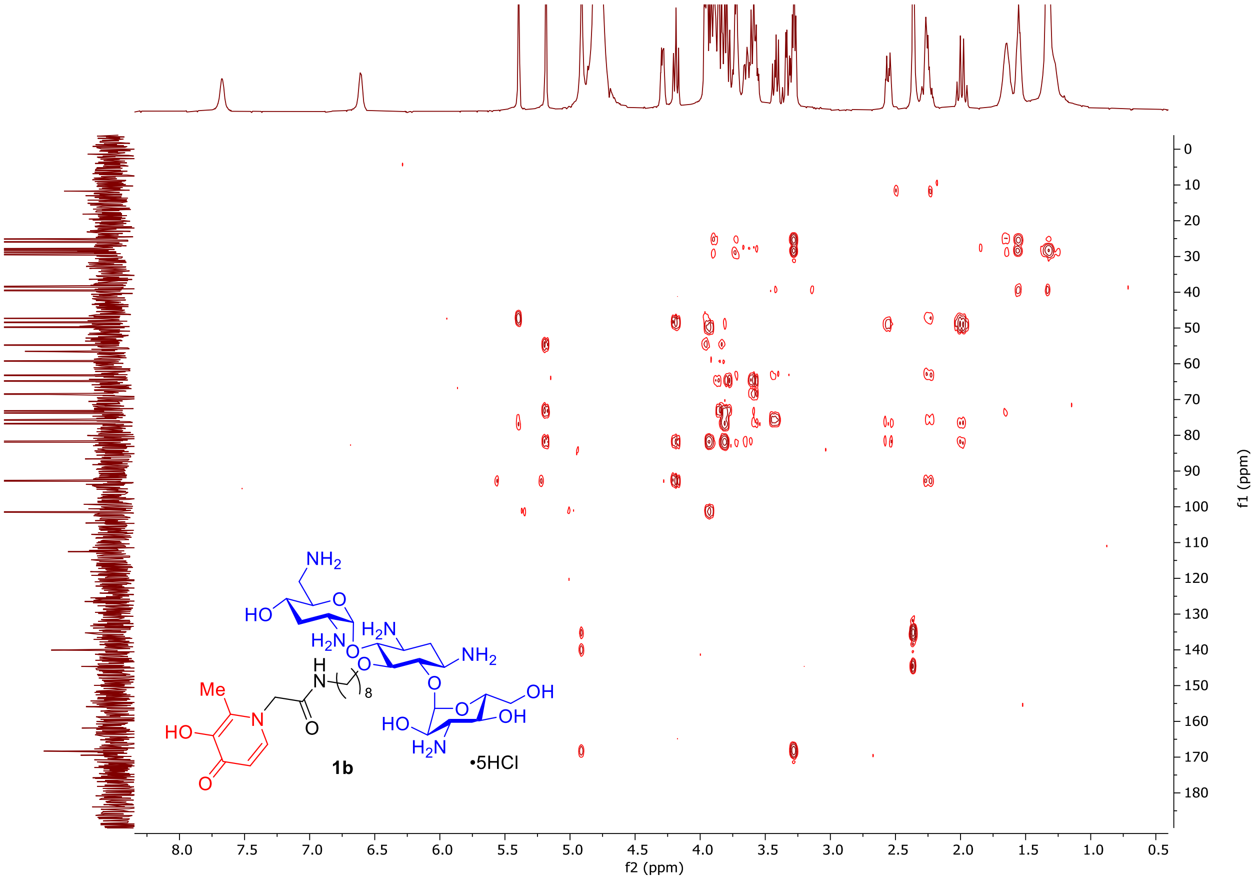

Figure S7: HMBC NMR spectrum of compound **1b** in D_2O

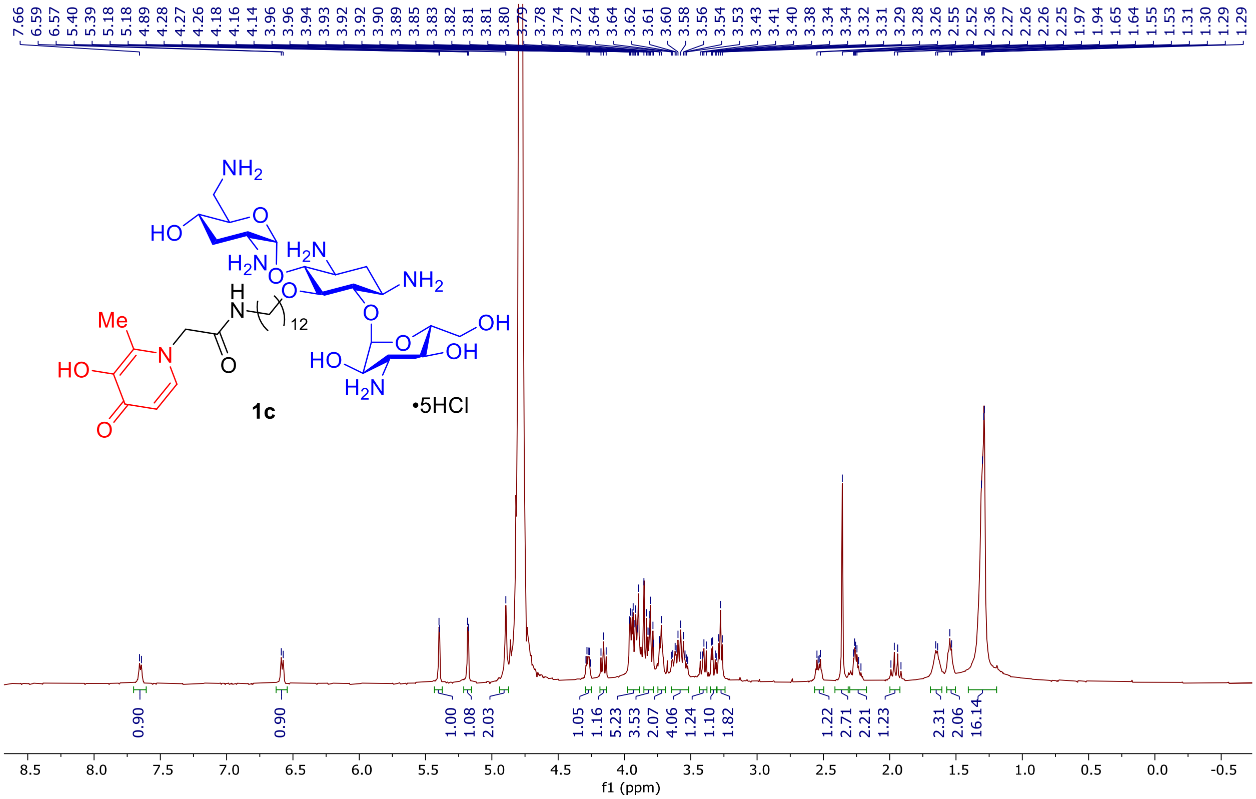

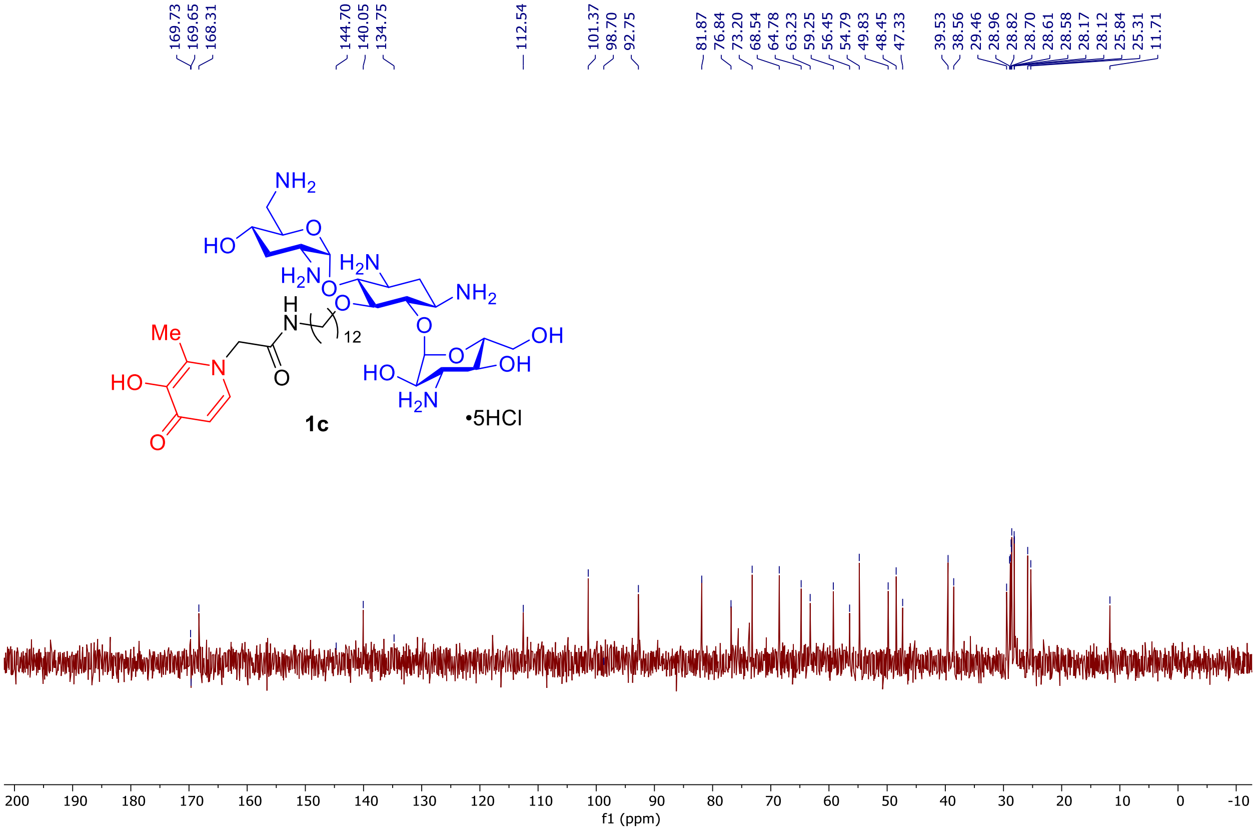

Figure S8: ^{1}H and ^{13}C NMR spectra of compound **1c** in D_2O

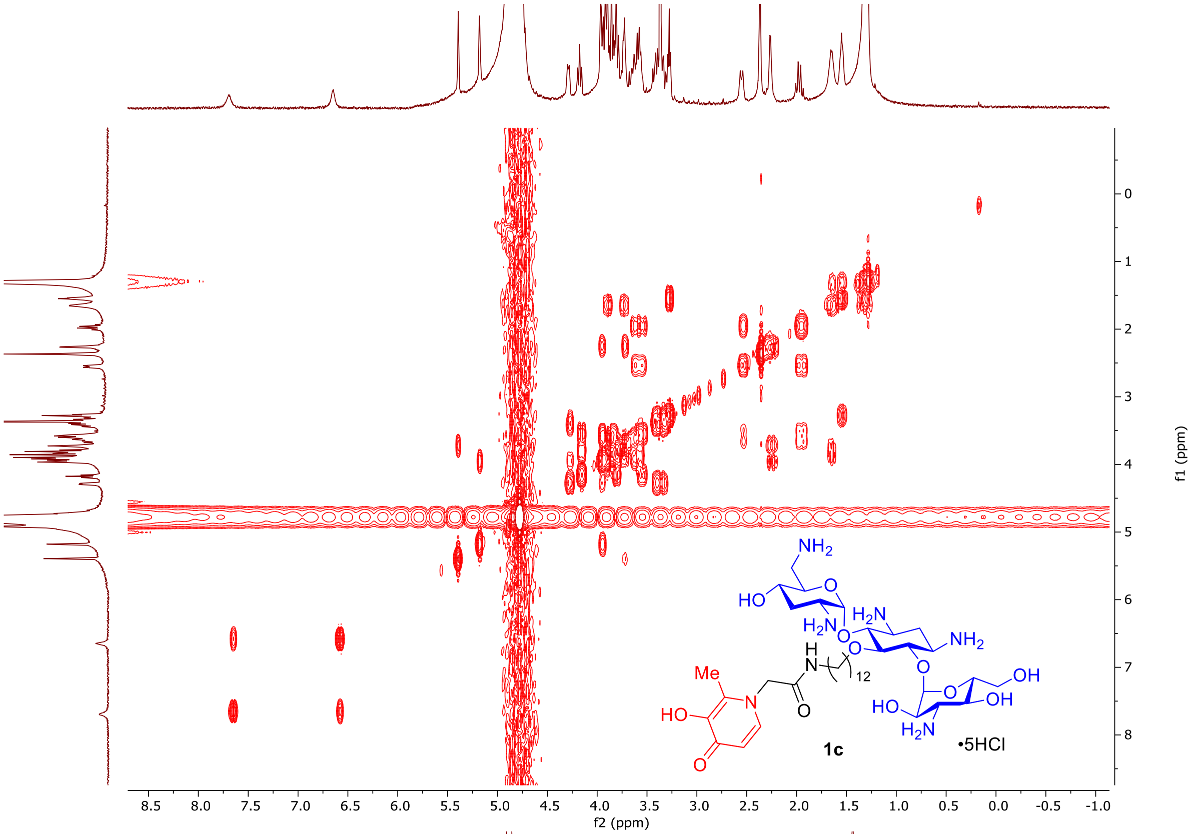

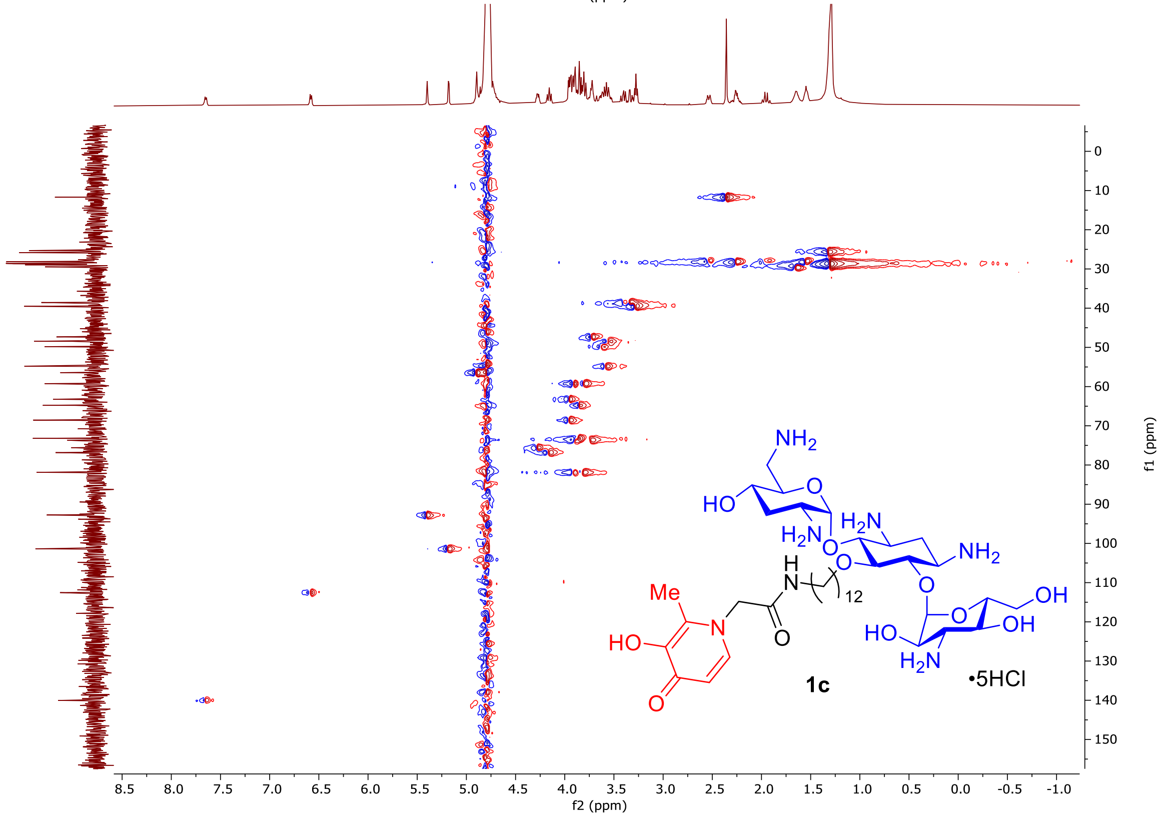

Figure S9: COSY and HSQC NMR spectra of compound **1c** in D_2O

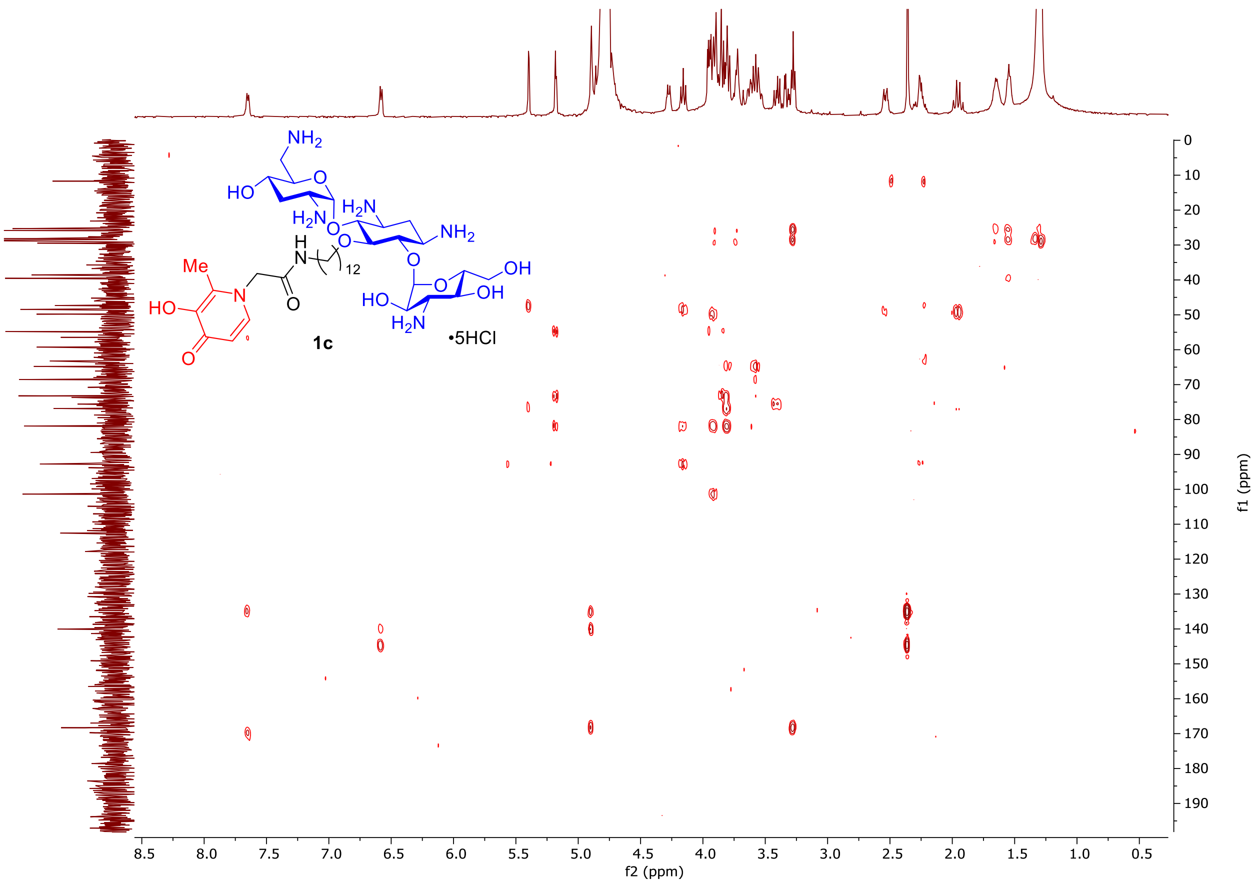

Figure S10: HMBC NMR spectrum of compound **1c** in D_2O

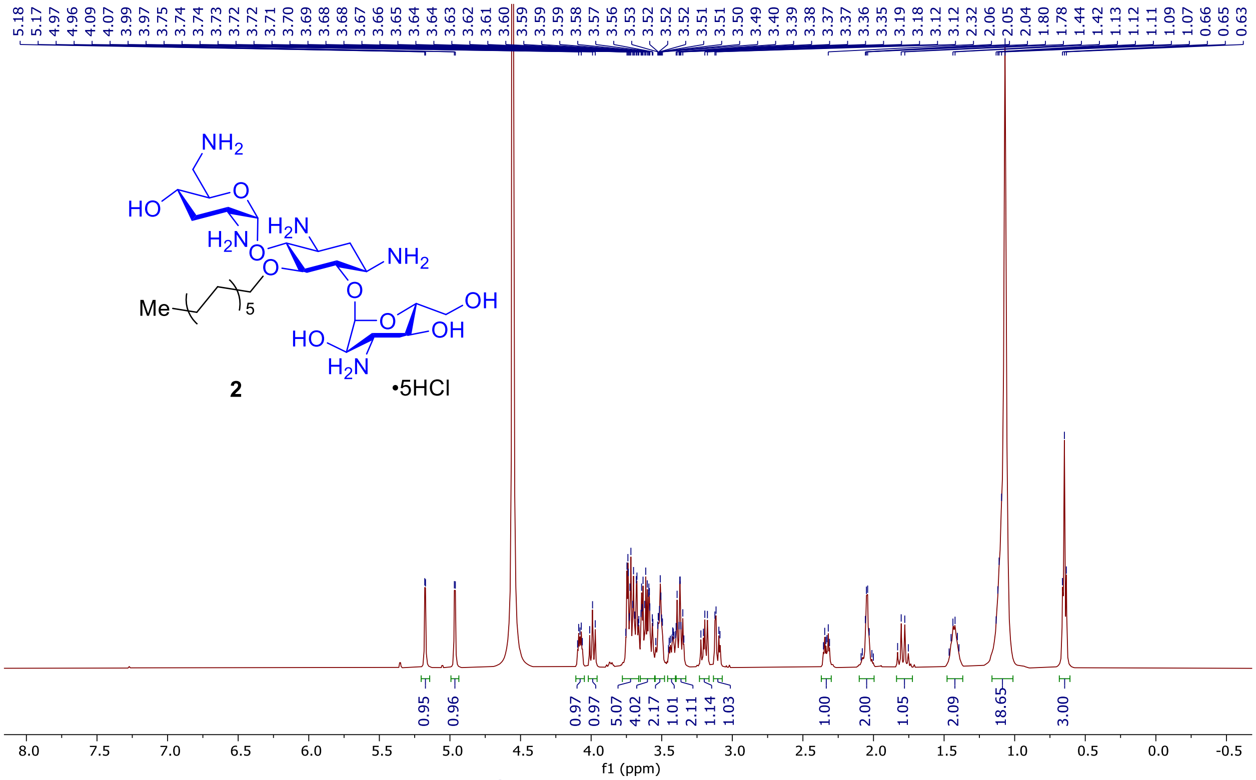

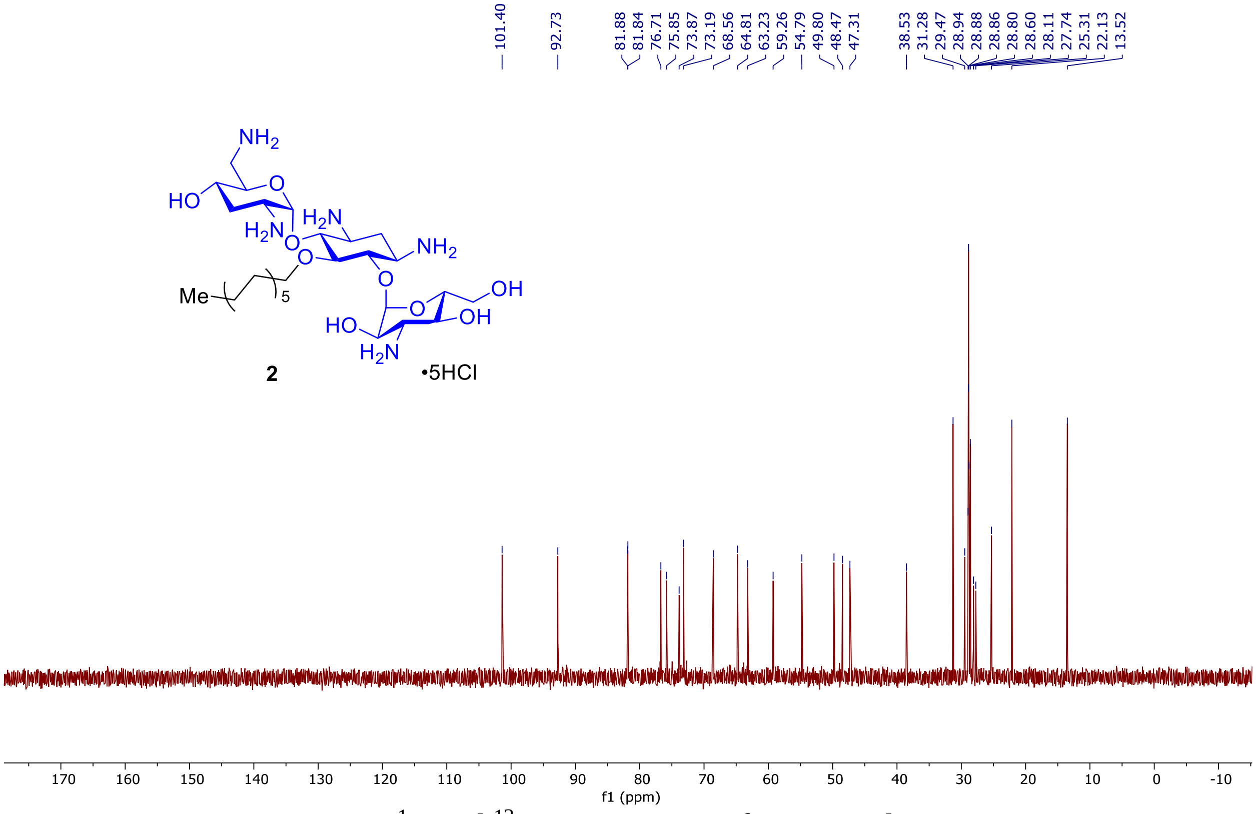

Figure S11: ^{1}H and ^{13}C NMR spectra of compound **2** in D_2O

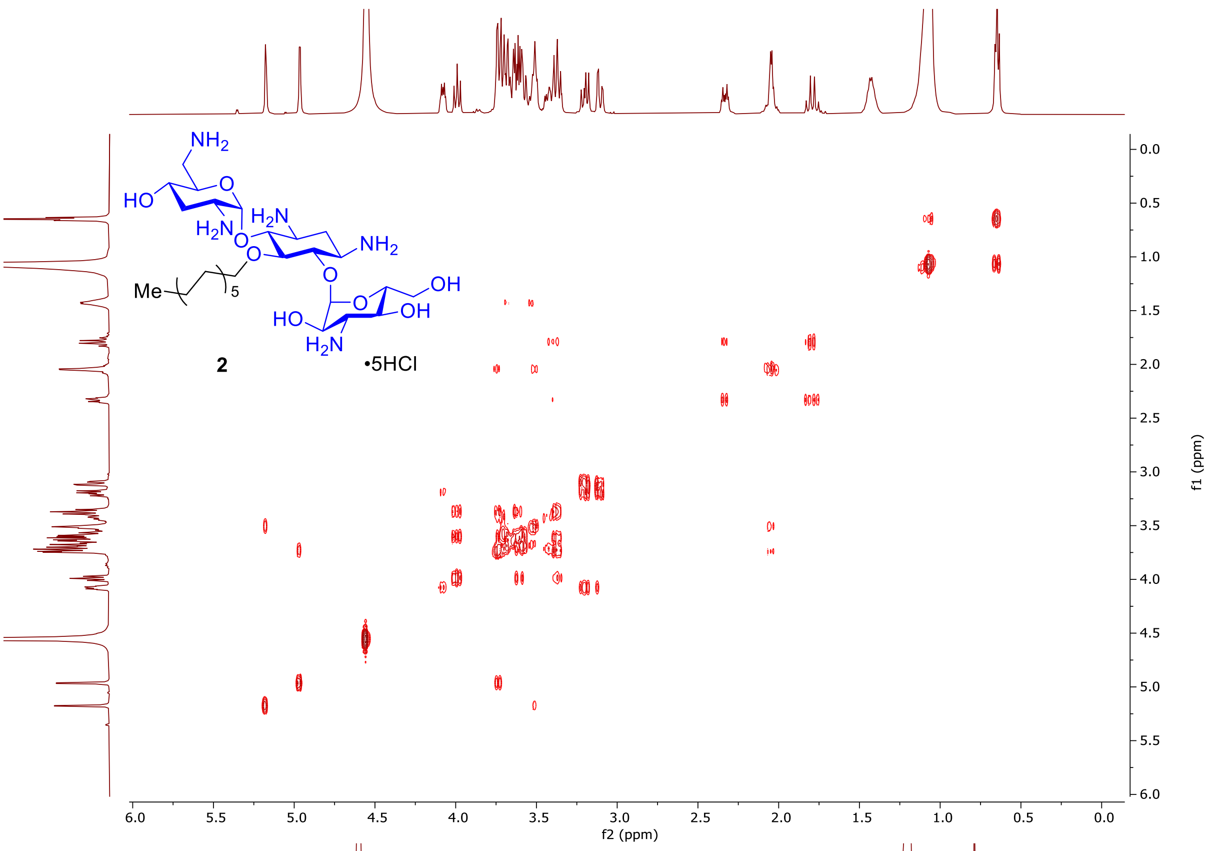

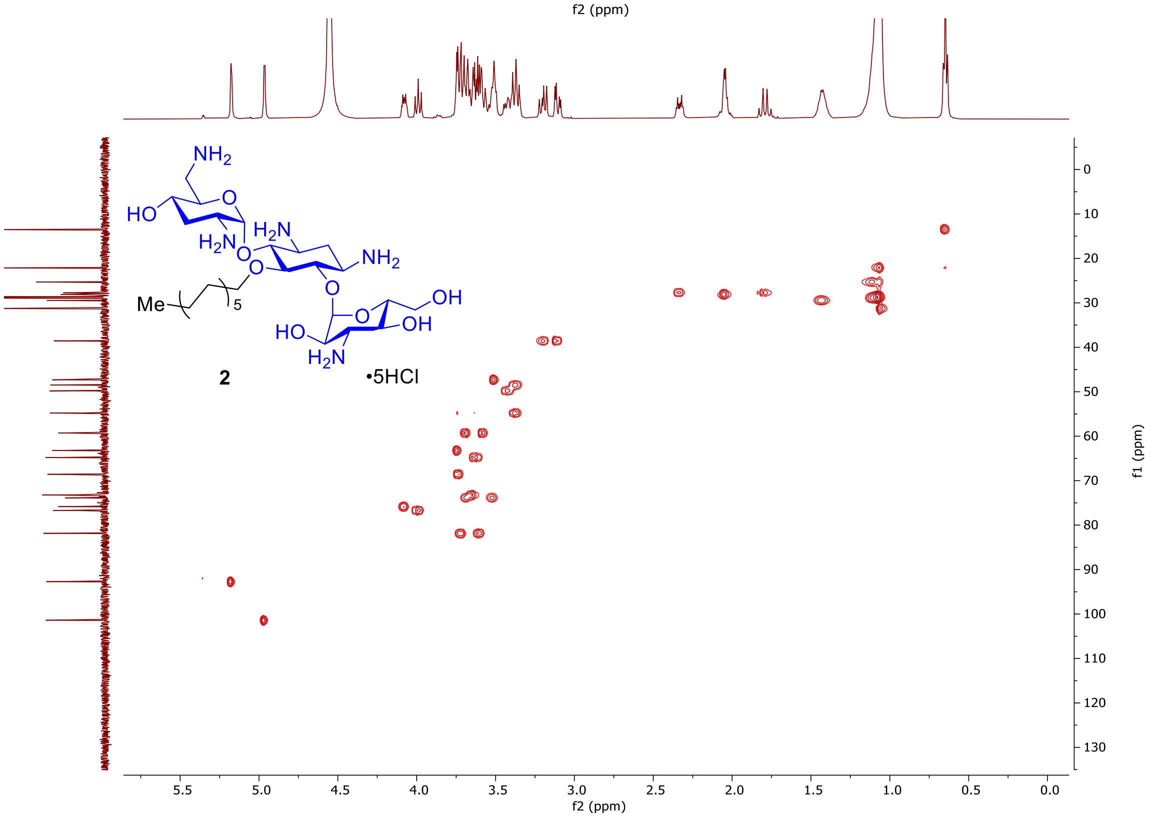

Figure S12: COSY and HSQC NMR spectra of compound **2** in D_2O

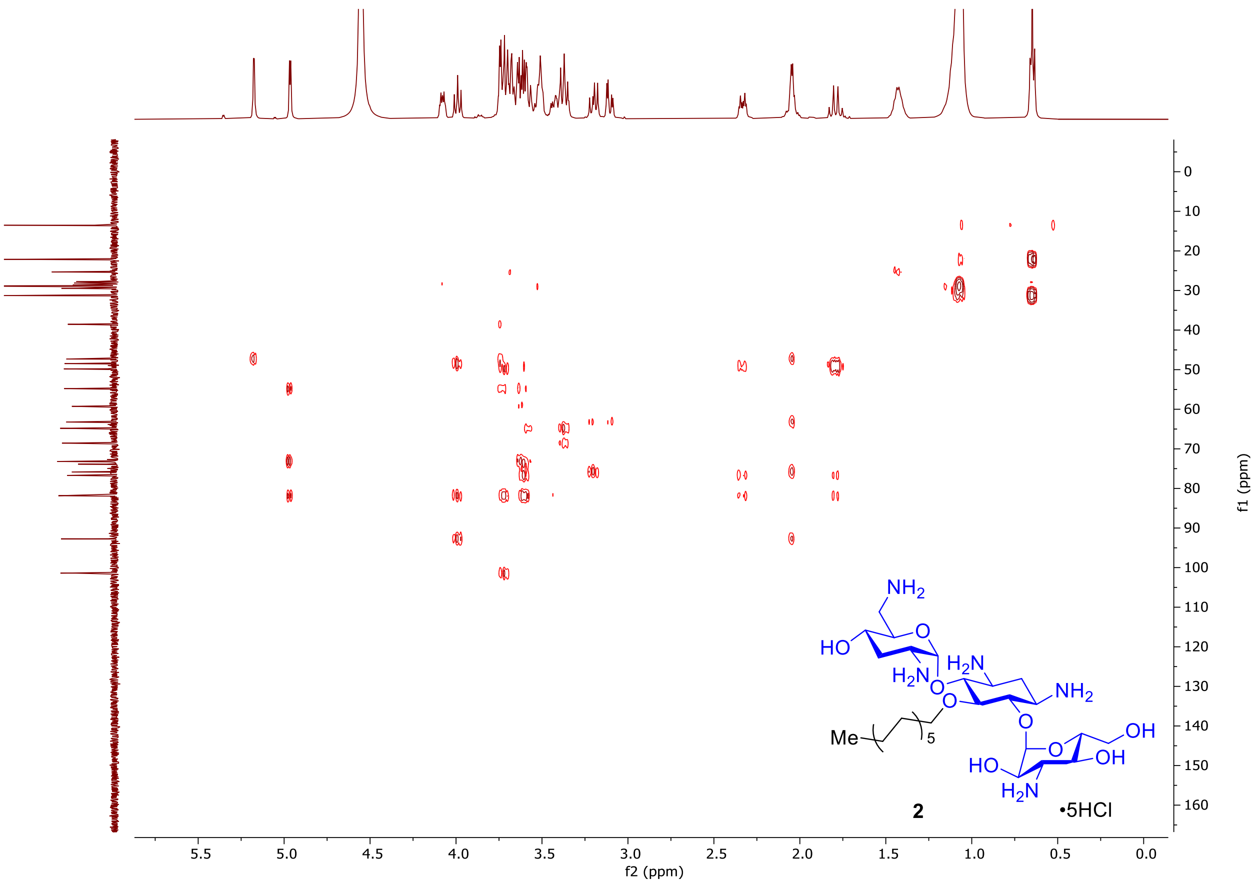

Figure S13: HMBC NMR spectrum of compound **2** in D_2O

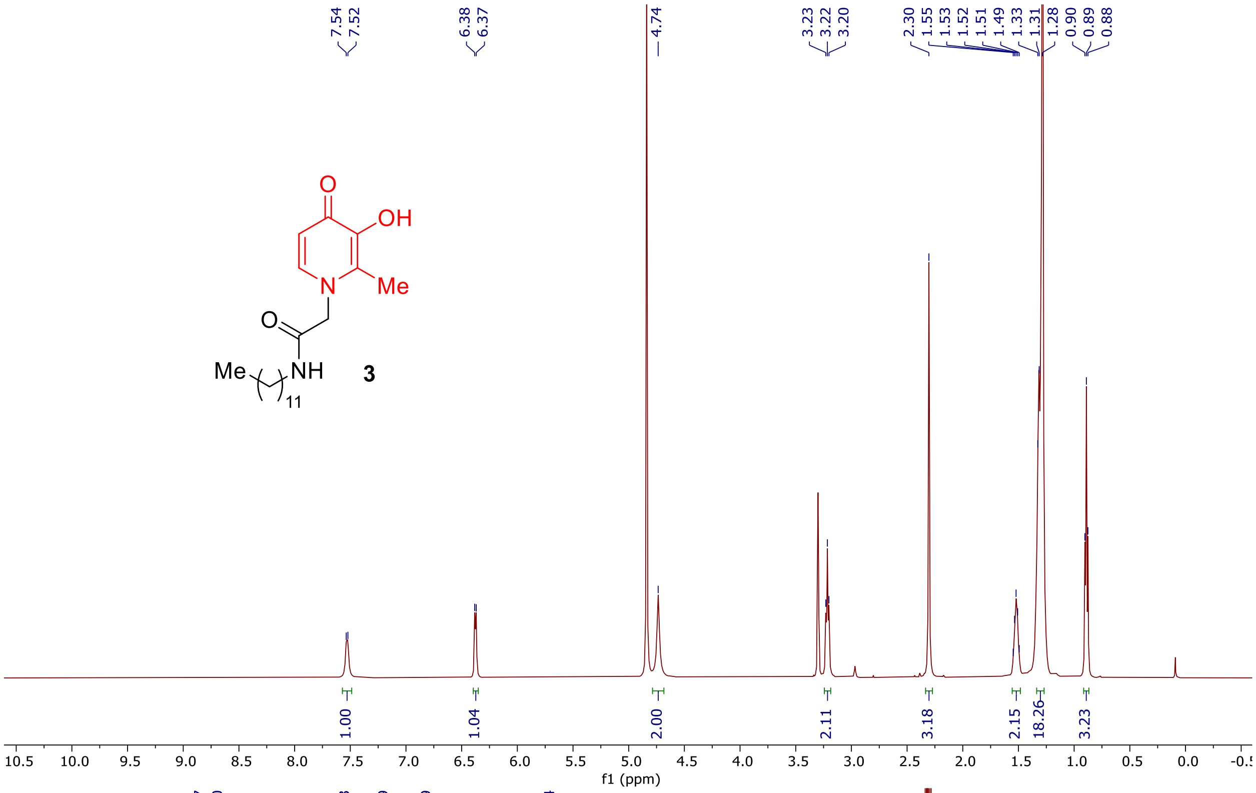

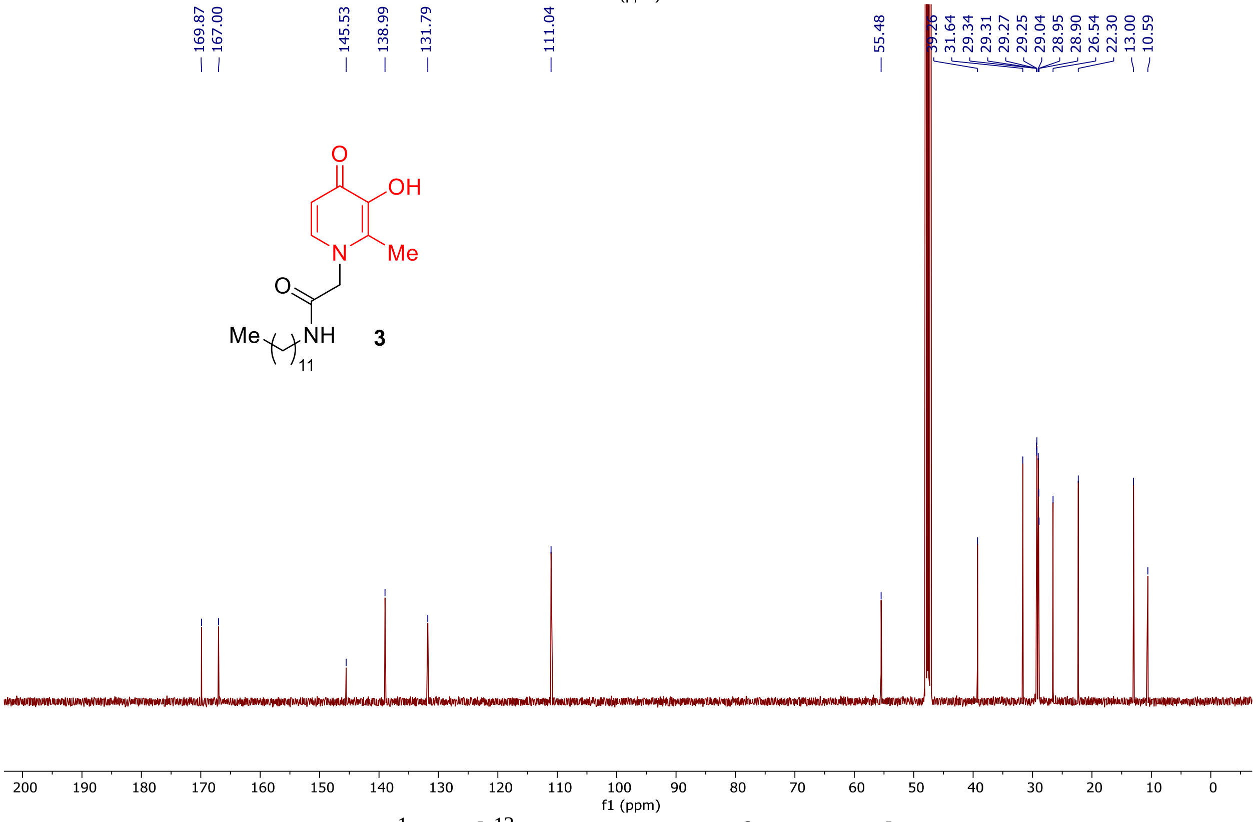

Figure S14: ^{1}H and ^{13}C NMR spectra of compound **3** in D_2O

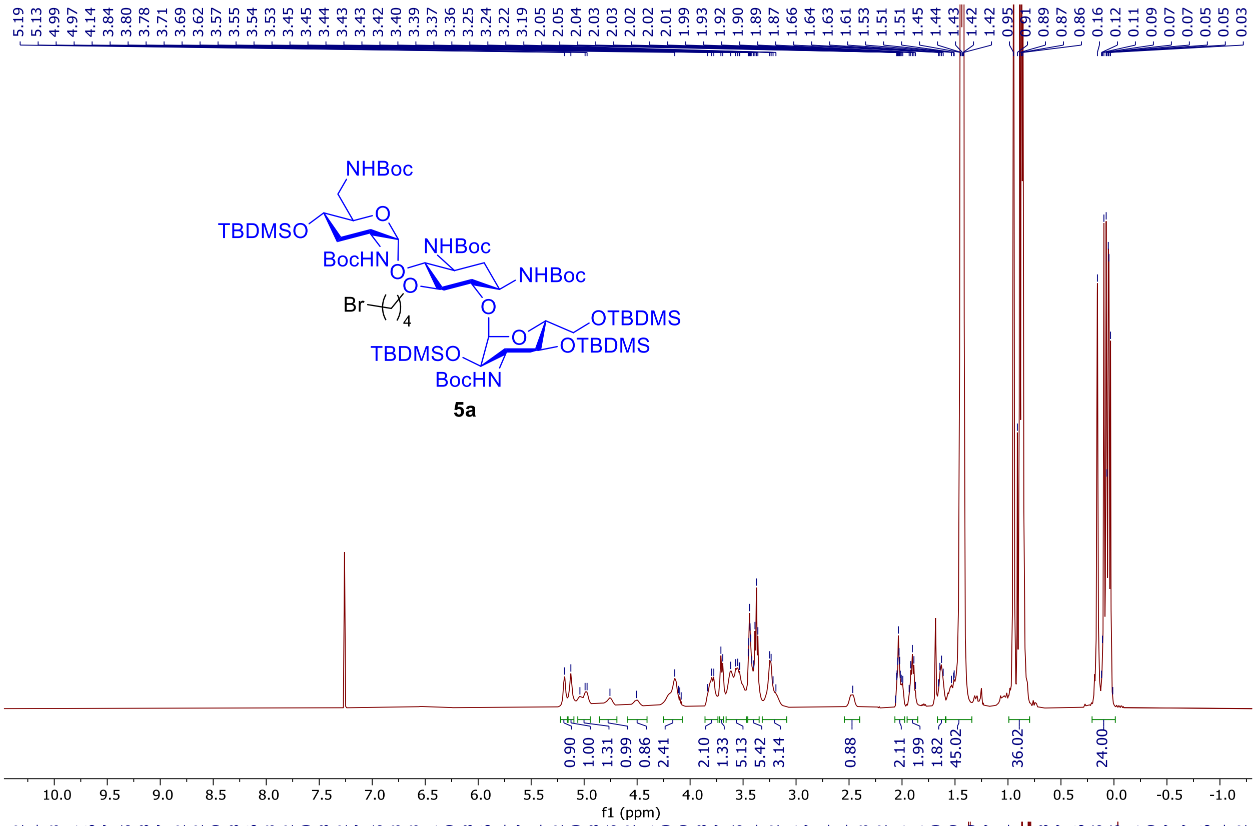

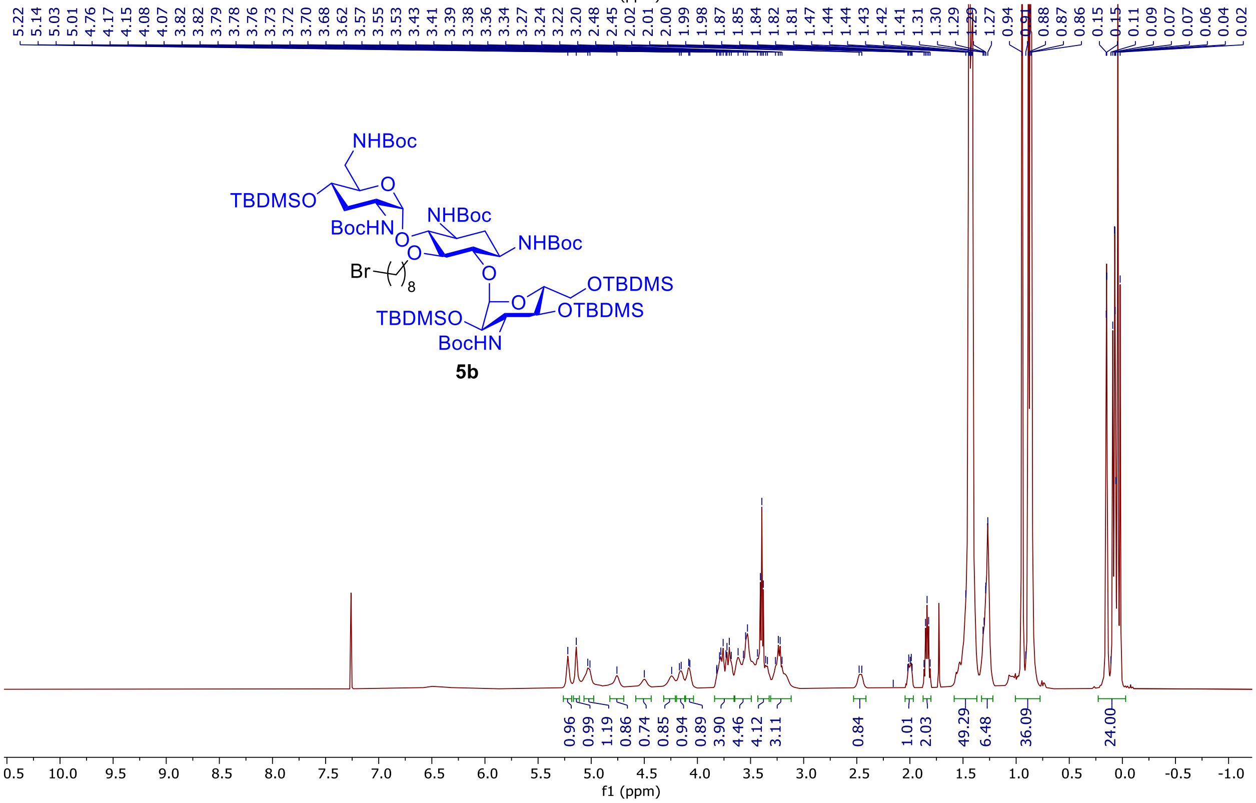

Figure 15. ^{1}H NMR spectra of compound **5a** and **5b** in $CDCl_3$

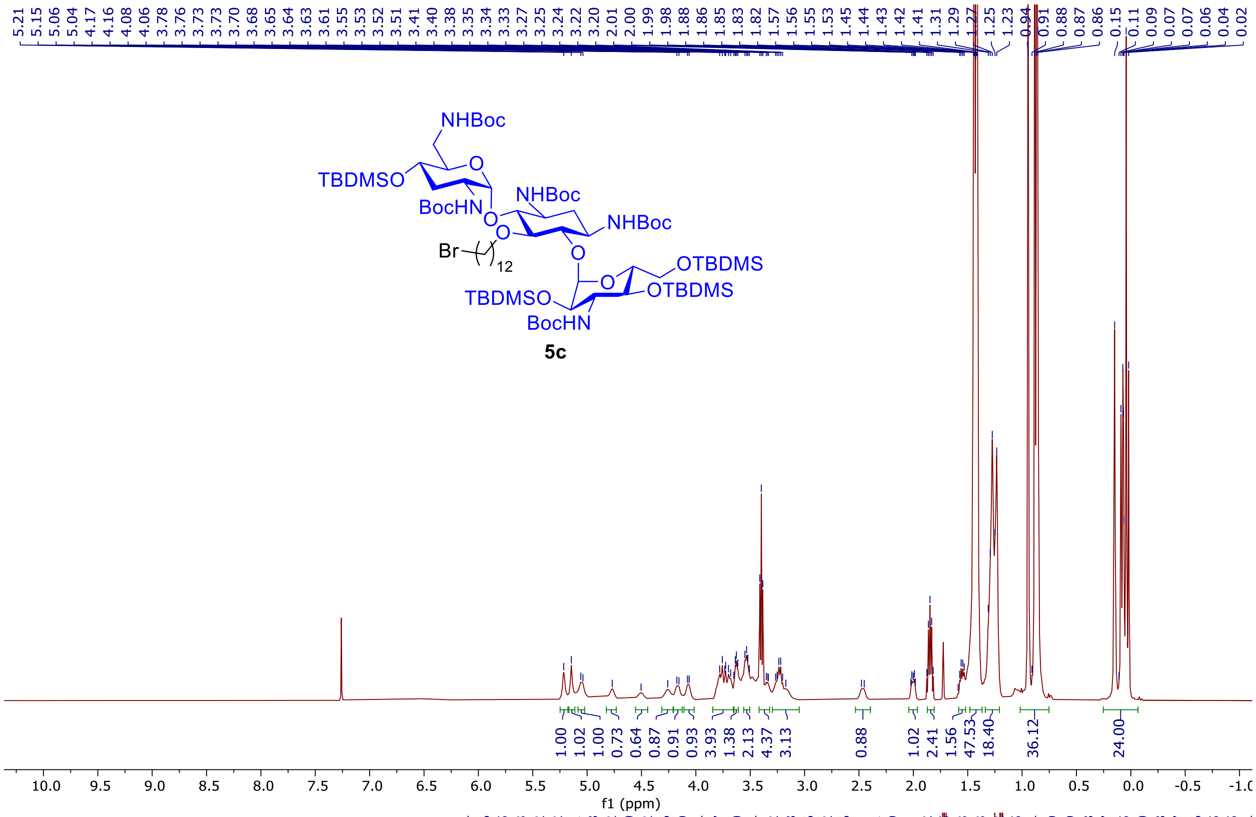

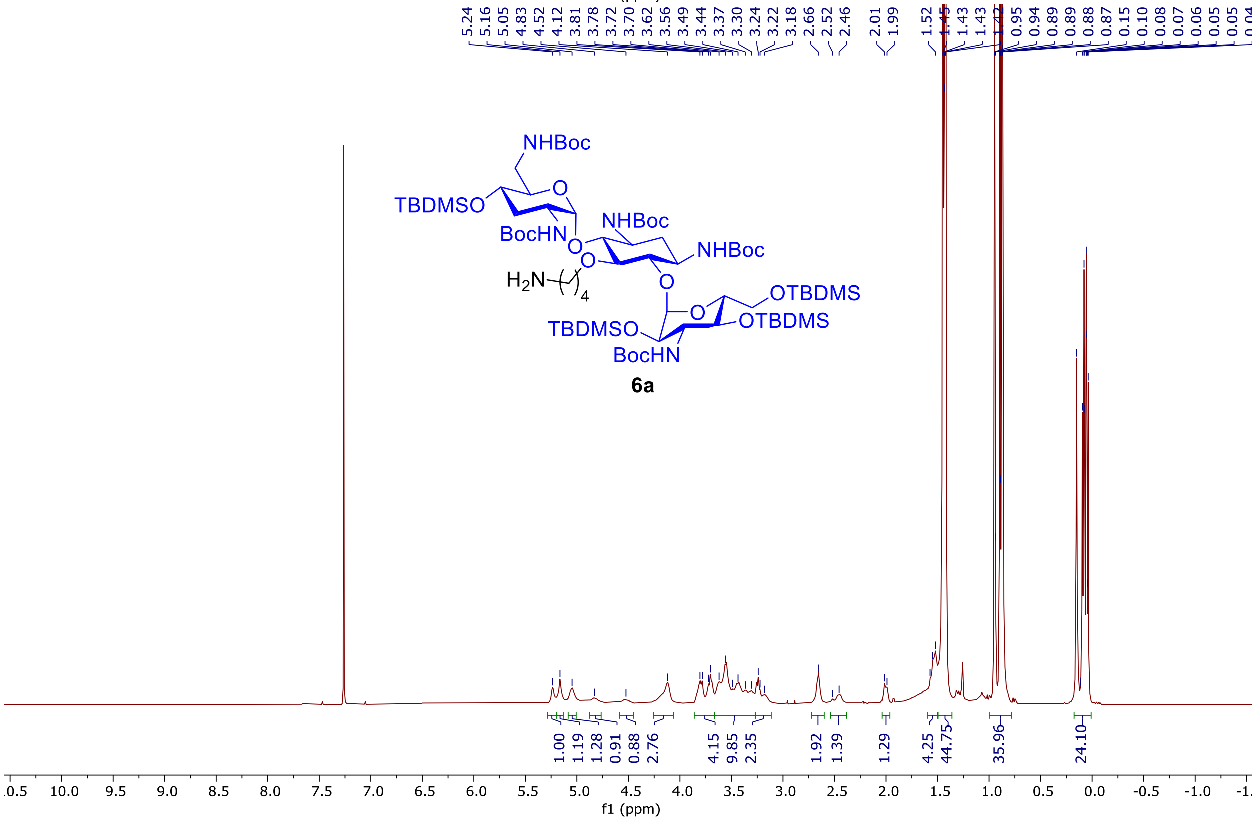

Figure 16. ^{1}H NMR spectra of compound **5c** and **6a** in $CDCl_3$

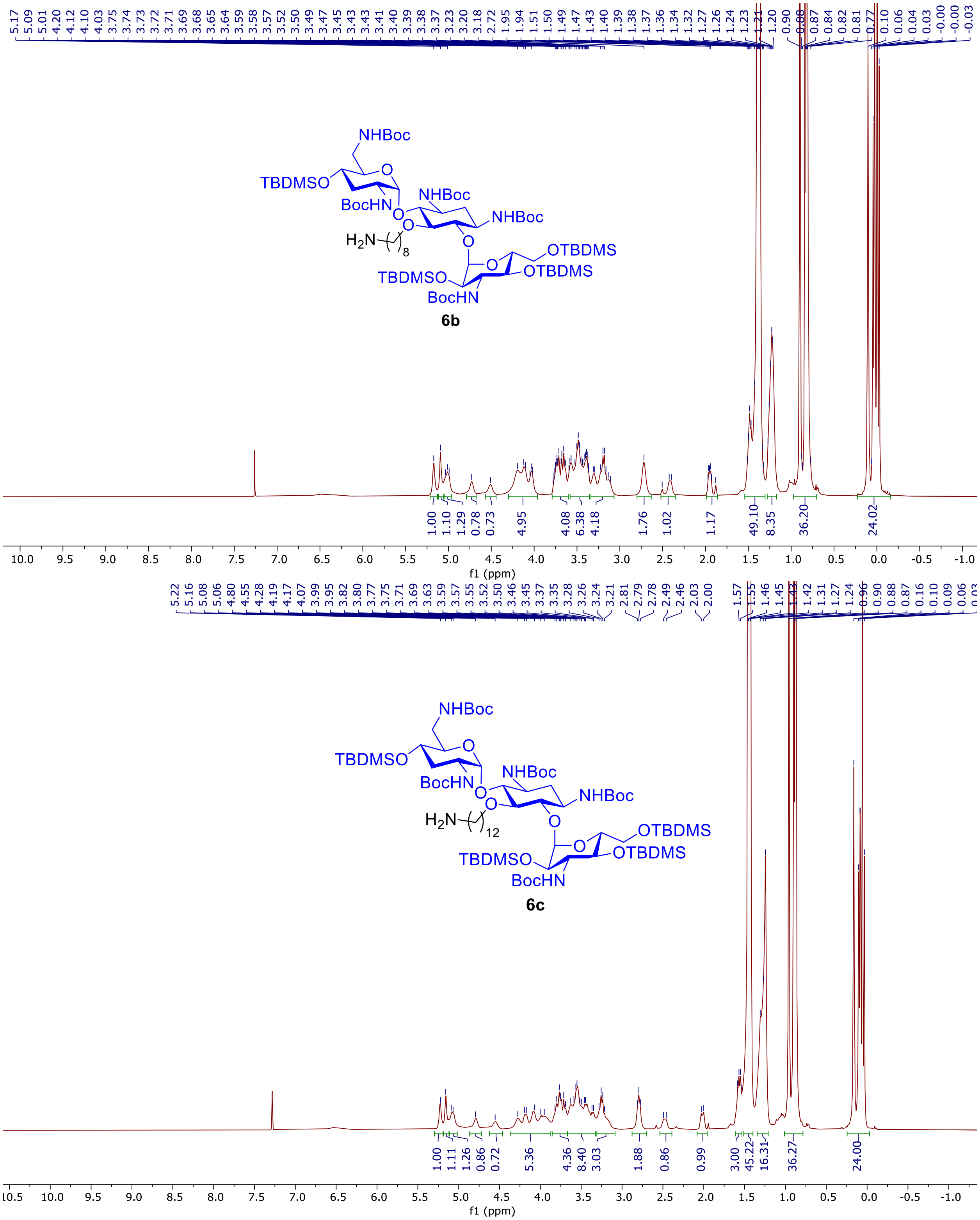

Figure S17: ^{1}H NMR spectra of compound **6b** and **6c** in $CDCl_3$

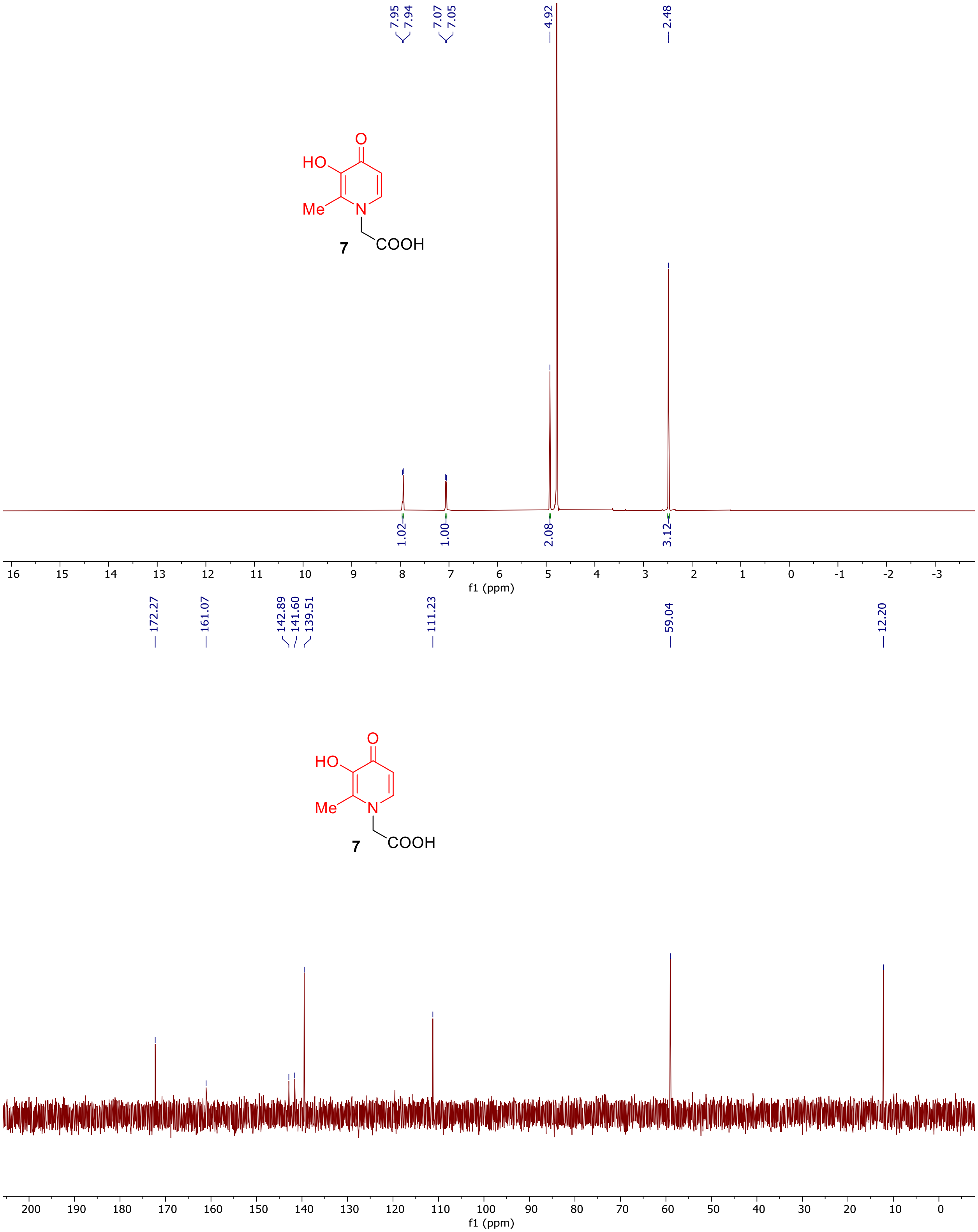

Figure S18: ^{1}H and ^{13}C NMR spectra of compound **7** in D_2O

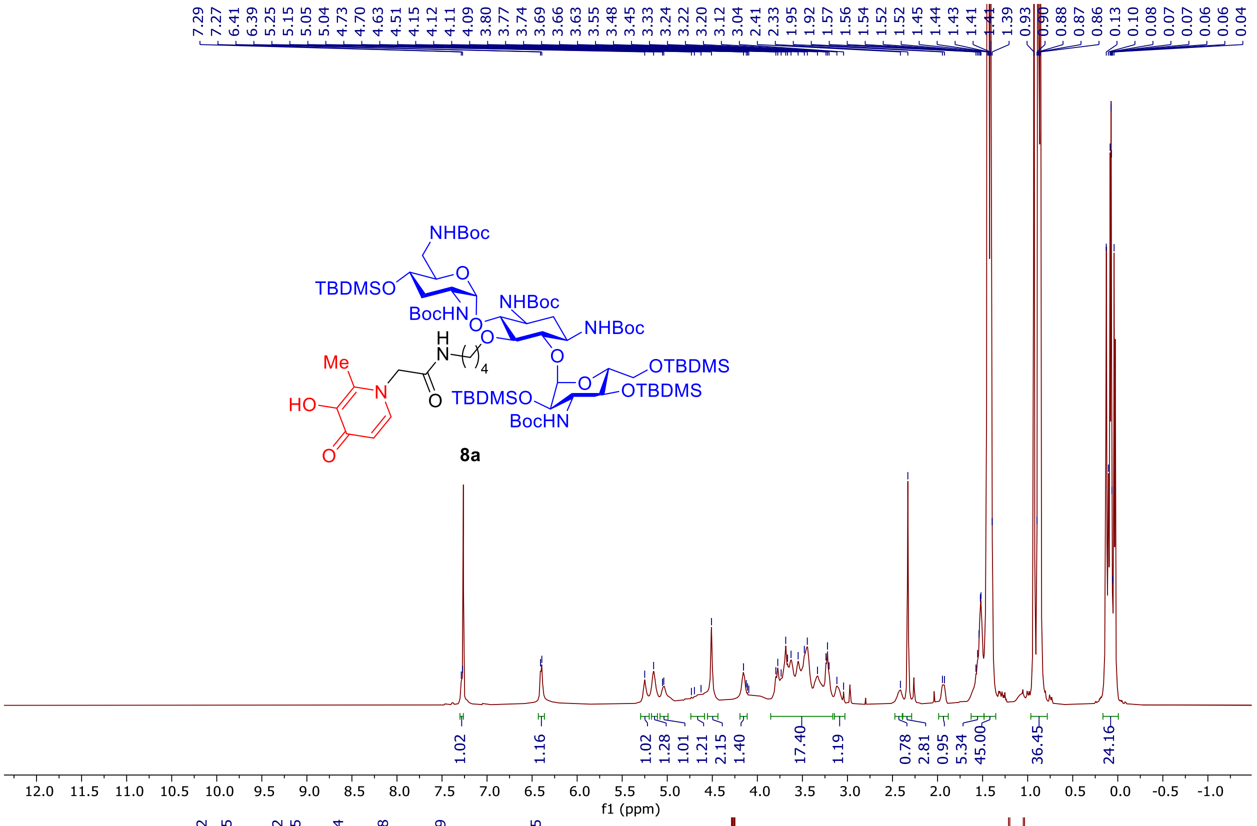

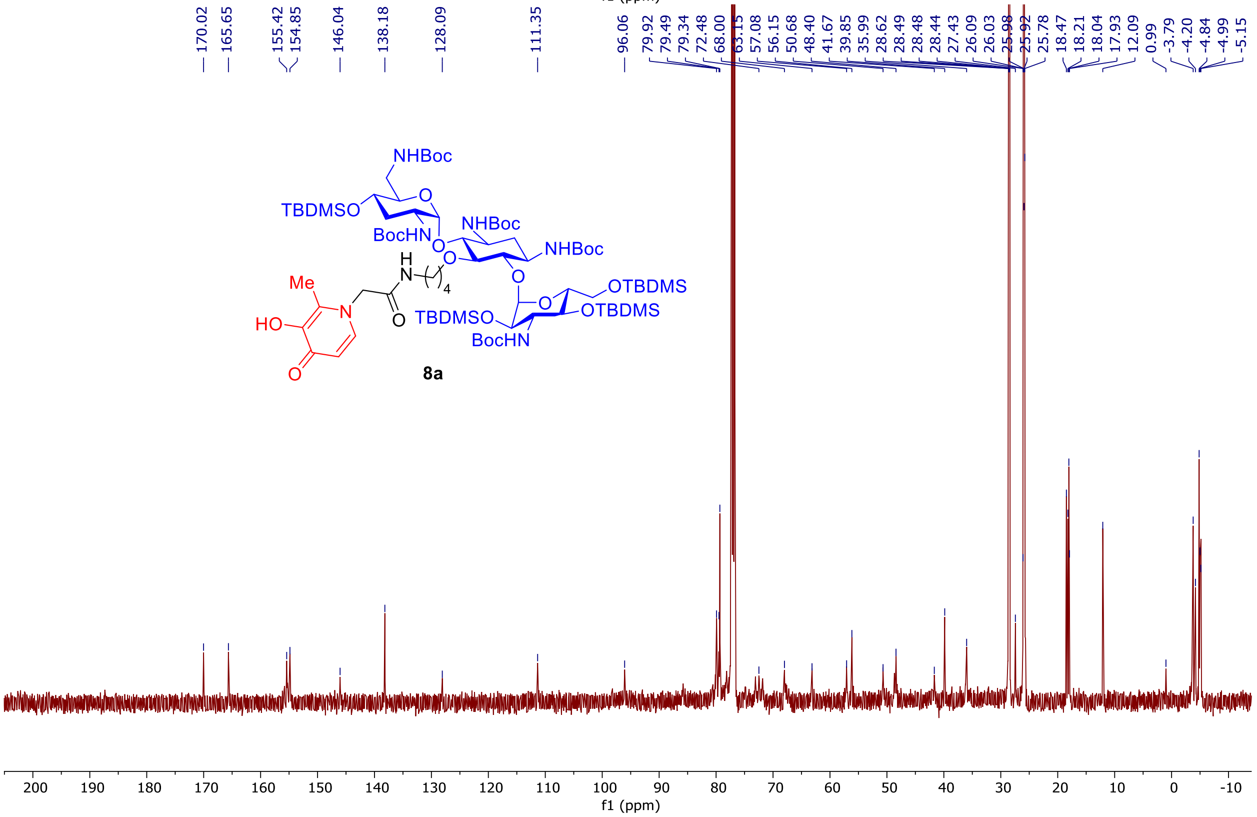

Figure S19: ^{1}H and ^{13}C NMR spectra of compound **8a** in $CDCl_3$

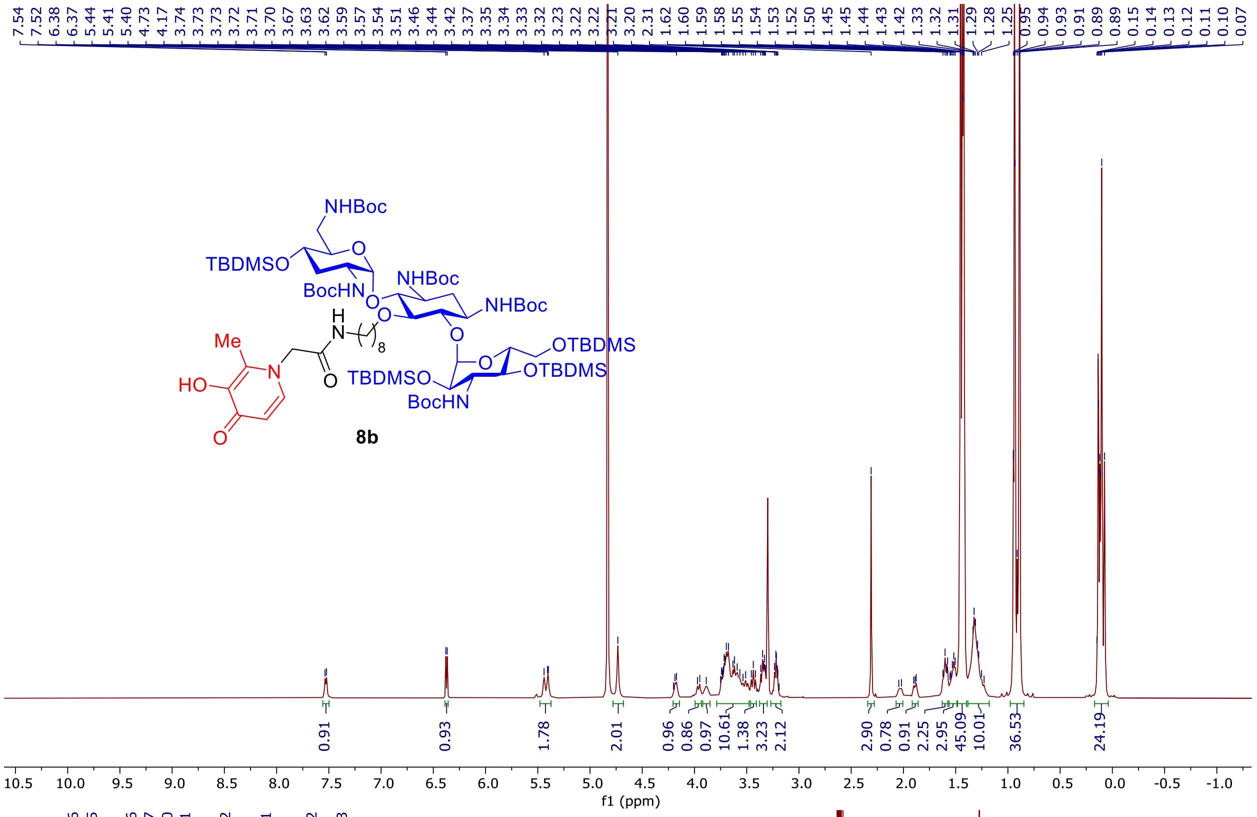

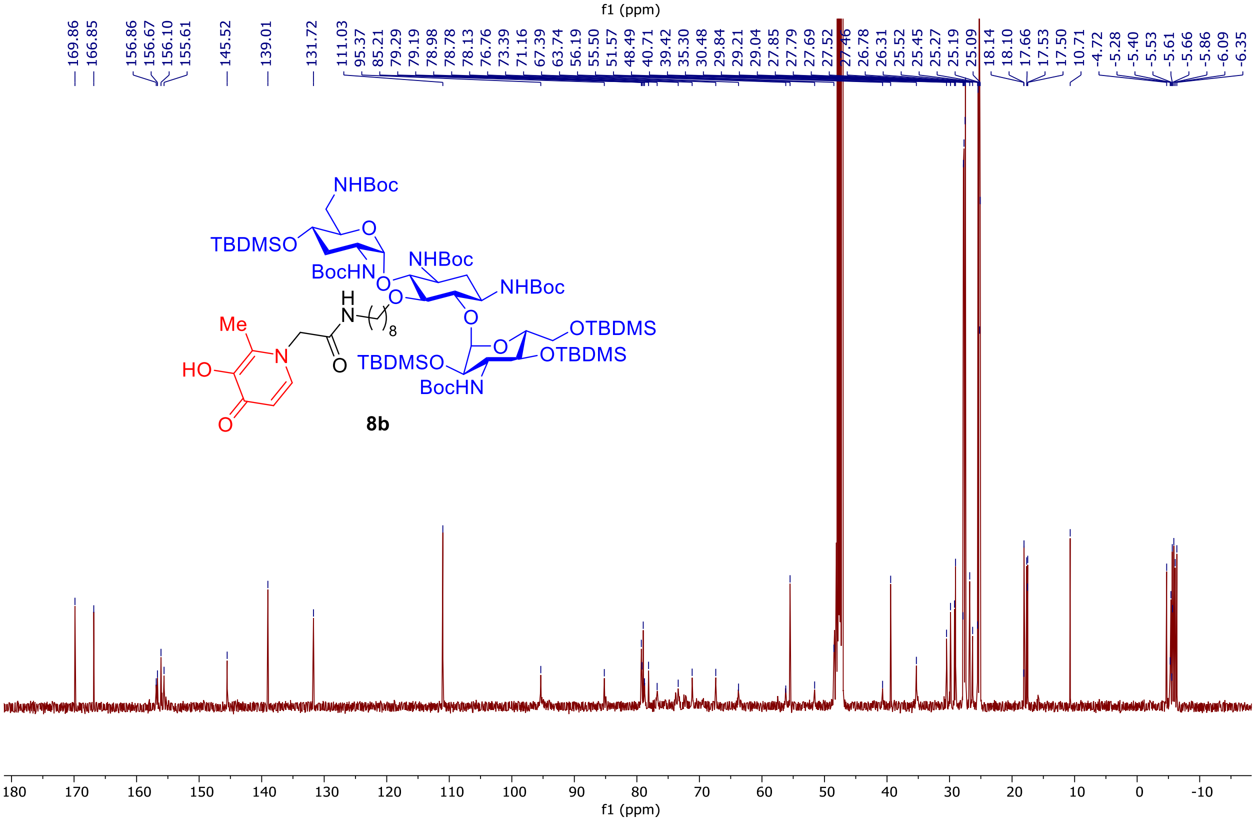

Figure S20: ^{1}H and ^{13}C NMR spectra of compound **8b** in $CDCl_3$

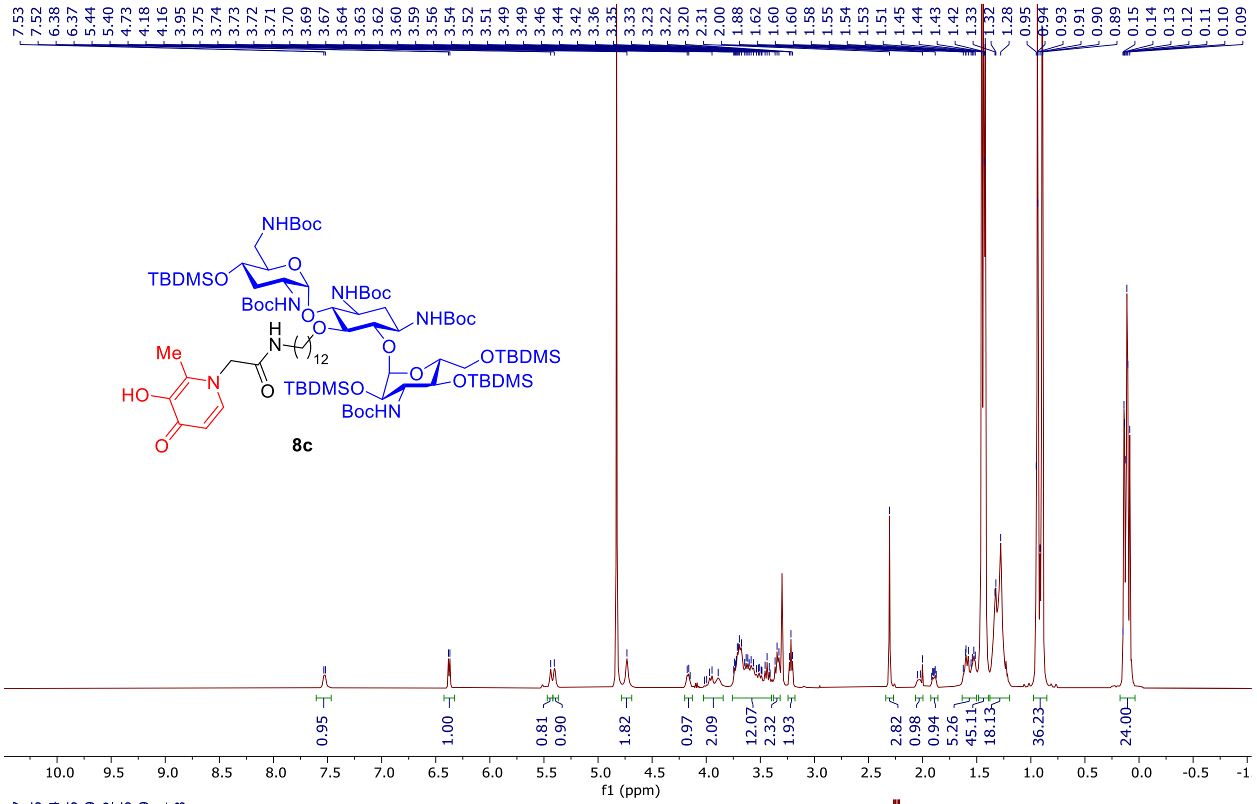

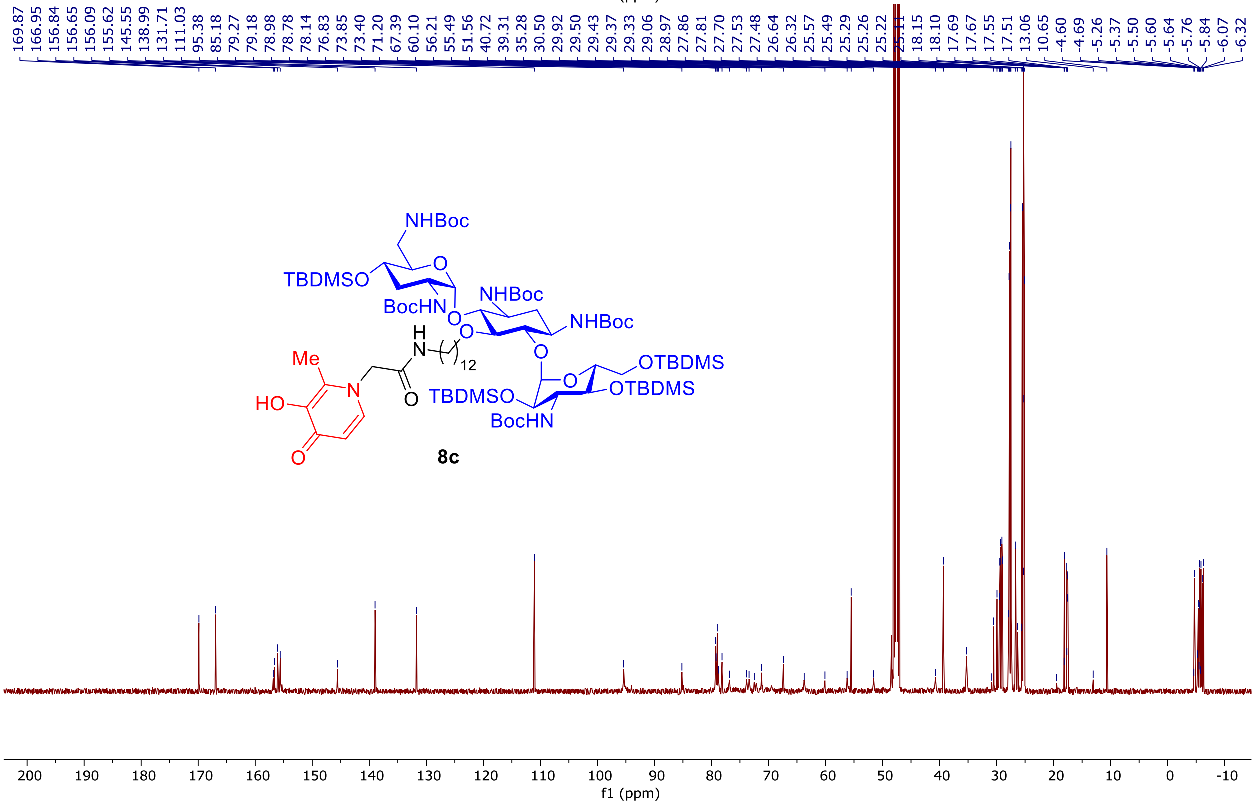

Figure S21: ^{1}H and ^{13}C NMR spectra of compound **8c** in $CDCl_3$

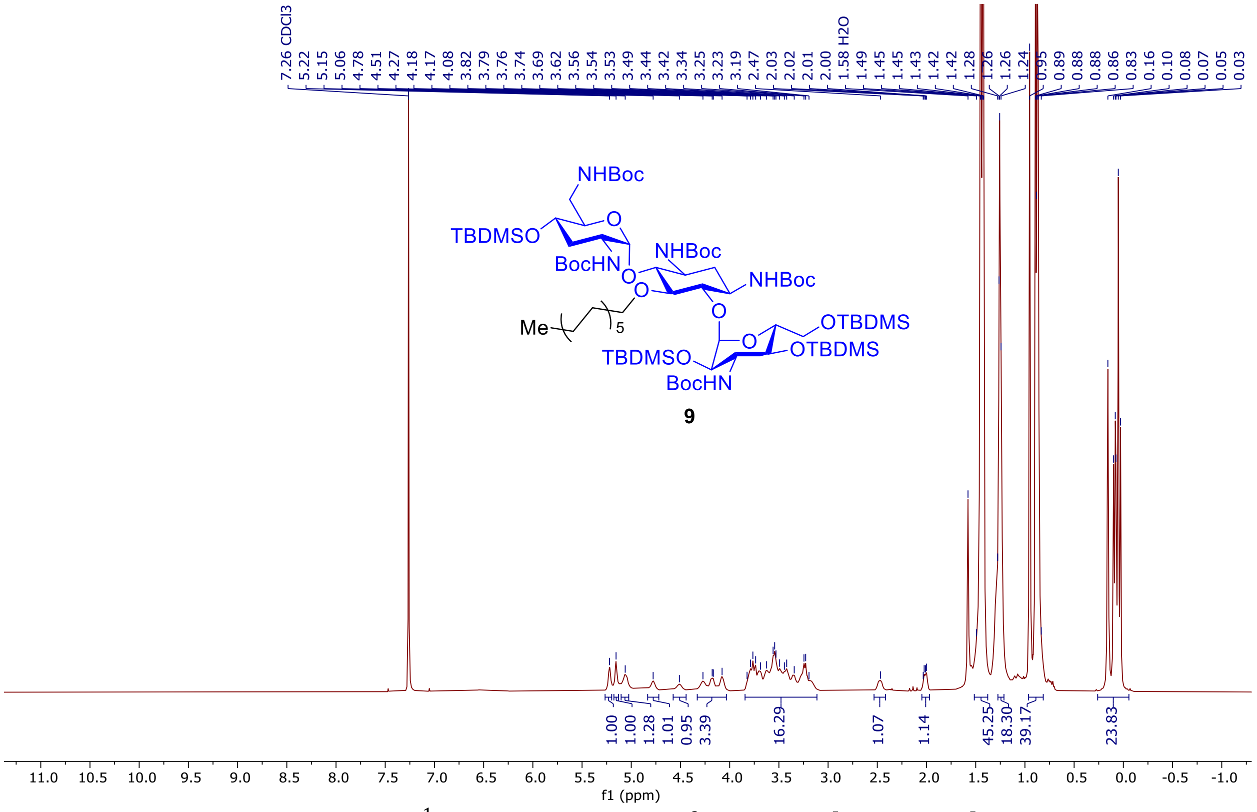

Figure S22: ^{1}H NMR spectrum of compound **9** in $CDCl_3$
